# Marine-derived *Acremonium* strain prioritization using untargeted metabolomics approach for the identification of cytotoxic cyclic depsipeptides

**DOI:** 10.1186/s12896-025-01065-2

**Published:** 2025-11-24

**Authors:** Sailesh Maharjan, Johan Isaksson, Teppo Rämä, Kine Østnes Hansen, Jeanette Hammer Andersen, Espen Holst Hansen

**Affiliations:** 1 Marbio, Faculty of Biosciences, Fisheries, and Economics, Norwegian College of Fishery Science (NFH), UiT-The Arctic University of Norway, Tromsø, 9037 Norway; 2 Department of Pharmacy (IFA), Faculty of Health Sciences, UiT-The Arctic University of Norway, Tromsø, 9037 Norway; 3 Department of Chemistry (IK), Faculty of Science and Technology, UiT-The Arctic University of Norway, Tromsø, 9037 Norway

**Keywords:** Cyclic depsipeptides, Cytotoxic, *Acremonium*, Antileukemic activity, Fungi, Ascomycota, Metabolomics

## Abstract

**Background:**

The Arctic environment, characterized by extreme conditions, hosts a largely untapped reservoir of fungal communities that have adapted to these harsh conditions by producing specialized bioactive secondary metabolites. Among these, *Acremonium* species also remain underexplored, despite their potential to produce structurally diverse and biologically active secondary metabolites. This is largely due to difficulties in sampling in remote Arctic regions and limited research focus on fungi from such environments. This study aimed to use an integrated workflow combining metabolomics, chemometrics, and bioactivity screening to prioritize *Acremonium* strains for the identification of bioactive secondary metabolites. We applied this workflow to investigate six *Acremonium* strains associated with driftwood from the Arctic: *A. ellipsoideum* (F1, F2), *A. synnematoferum* (F3, F4, F5), and *A. multiramosum* (F6), aiming to identify cytotoxic secondary metabolites.

**Results:**

The integrated metabolomics and chemometrics approach revealed unique chemical fingerprints for *A. ellipsoideum* (F1) and *A. synnematoferum* (F5) among the six strains. By further combining bioactivity screening results, strain F5 was prioritized for further detailed study. Five compounds were isolated from F5 and structurally elucidated as cyclic depsipeptides: destruxin-A4 chlorohydrin (**1**), trichomide D (**2**), destruxin-A5 (**3**), homodestruxin (**4**), and homodestcardin (**5**). All compounds exhibited cytotoxic effects against the tested cell lines but did not exhibit activity in the targeted bioassays against the kinase FLT3 or the phosphatase PTP1B. Cytotoxic destruxin-type compounds likely play a significant ecological role, as they have been reported to exhibit entomopathogenic, nematocidal, and phytotoxic effects.

**Conclusion:**

The identification of five known cytotoxic destruxin-type depsipeptides from *A. synnematoferum* as a new source expands the chemical diversity known for this genus and underscores their potential for the development of cytotoxic agents. These findings highlight not only the value of Arctic fungi as a reservoir of bioactive compounds but also the necessity of deciphering the ecological roles of cytotoxic metabolites produced by these organisms as they adapt to extreme environments. Furthermore, this study highlights the effectiveness of multi-informative-driven strain prioritization in uncovering bioactive metabolites from new fungal sources, emphasizing the significance of exploring Arctic fungal diversity for its potential to enhance chemical diversity, contribute to drug development, and broaden our understanding of ecological roles.

**Supplementary Information:**

The online version contains supplementary material available at 10.1186/s12896-025-01065-2.

## Background

The Arctic region, characterized by strong seasonal climate variations, offers a distinctive reservoir of fungal diversity [1–3]. Fungal inhabitants of this harsh environment are often referred to as extremophiles, as they must withstand a range of environmental stressors such as low temperatures and seasonally high UV radiation [4–6]. Many of these fungi have evolved to produce specialized secondary metabolites necessary in environmental adaptation, and Arctic fungi have therefore attracted increasing interest as a potential source of new bioactive molecules with pharmaceutical applications [7]. Among them, *Acremonium* species are of significant interest due to their proven history as notable producers of a wide array of bioactive secondary metabolites such as steroids, terpenoids, polyketides, non-aromatic polyketides, alkaloids, and peptides [8].

The genus name *Acremonium* has been applied to classify hundreds of filamentous fungi since its introduction by Link (1809) [9]. Mycobank currently lists 251 names with the genus *Acremonium* (http://www.MycoBank.org). Acremonium-like fungi are widely distributed across a broad range of environments worldwide [10, 11]. They are recognized as a highly ubiquitous and taxonomically intricate group of ascomycetes, possibly due to their high phenotypic plasticity and ability to thrive on diverse substrates [12]. *Acremonium* and acremonium-like species are asexual, simply structured filamentous fungi. Morphologically, they are characterized by hyaline, septate hyphae giving rise to narrow, tapered, unbranched or sparsely branched phialides with single-celled conidia in slimy heads [12–14]. A recent and comprehensive revision has shown that acremonium-like fungi are highly polyphyletic, with species belonging to three orders within the class *Sordariomycetes*. The genus *Acremonium sensu stricto* was shown to be placed within the order *Hypocreales* and the family *Bionectriaceae*, currently comprising 19 species with available sequence data [12]. As of September 2023, a total of 627 compounds had been reported from acremonium-like fungi, 115 of which were newly reported between Dec 2016 and Sep 2023 [8, 15]. This shows their continued value in natural product discovery. Despite the high number of metabolites that have already been isolated, many *Acremonium sensu lato* species have remained chemically unexplored, especially those isolated from the Arctic region, which are still underrepresented in the literature [16–18].

Mass spectrometry (MS) is a common and effective analytical tool used to study metabolites produced by fungi. It provides detailed molecular information, such as molecular weights, fragmentation patterns, isotopic distributions, and elemental compositions, enabling identification and dereplication of potentially bioactive compounds at an early stage of drug discovery (prior to isolation). High-resolution MS (HRMS) instruments such as QTof and Orbitrap are highly sensitive and can detect extremely low-abundance molecules, even at trace levels. This makes data analysis challenging, especially when analyzing complex mixtures of metabolites in crude extracts. When combined with chemometrics, MS becomes even more powerful, allowing the extraction of meaningful patterns and insights from complex datasets. The integration of metabolomics with chemometrics enhances the interpretation of MS data by identifying relationships, clustering similar samples, and prioritizing compounds of interest. It is useful in metabolomics, where larger datasets are generated and subtle differences between samples need to be identified. The objective of this project was to apply mass spectrometry-based metabolomics in combination with chemometrics to broadly investigate secondary metabolites of driftwood-associated fungi, aiming to distinguish phylogenetically closely related *Acremonium* species and prioritize strains for isolation and characterization of bioactive metabolites.

## Materials and methods

### General

An Acquity I-class UHPLC (Waters, Milford, MA, USA) coupled to Vion^®^ IMS QTof mass spectrometer (Waters, Milford, MA, USA) was used for (UHPLC-HRMS^E^) high-resolution mass spectrometry for metabolomic profiling of the extracts and fractions, and to analyze the pure compounds controlled by UNIFI 1.9.4 Scientific Information System. Mass data were acquired in HDMS^E^ mode, a data-independent acquisition (DIA) technique that combines ion mobility separation (IMS) with MS^E^ (all-ions fragmentation) to provide separation based on size, shape, and charge of analytes. The column used to separate the compounds was an Acquity UPLC^®^ BEH C18 column (100 mm × 2.1 mm, 1.7 μm), maintained at 40 °C. Mobile phase A (H_2_O + 0.1% formic acid) and B (MeCN + 0.1% formic acid) were maintained at a flow rate of 0.45 mL/min with a gradient program as follows: 10%−100% B over 12 min, hold at 100% B for 1.5 min, re-equilibrate at 10% B for 1 min. The mass spectrometer was operated with the following parameters: ionization mode ESI^+^; capillary voltage 0.8 kV; cone voltage 30 V; source temperature 100 °C; desolvation temperature 350 °C; collision gas N_2_. Mass data was acquired in HDMS^E^ mode, covering a mass range of 150−2000 Da at a scan rate of 0.2 scans per second. The low collision energy was set to 5 eV, and the high-energy ramp was 20–60 eV. MS data was calibrated through the constant infusion of a leucine enkephalin lock mass solution (100 pg/µL) at a rate of 5 µL/min.

Compound isolation guided by mass detection was performed using a preparative HPLC-MS system (Waters Auto Purification LCMS system equipped with a 2996 Photodiode Array Detector and a 3100-Mass spectrometer operated by MassLynx version 4.1). In the first isolation step, a Sunfire C18 OBD prep column (250 mm x 10 mm, 5 μm) was utilized, while in the second round of isolation step, either an XSelect CSH Phenyl-Hexyl Prep column (250 mm x 10 mm, 5 μm) or an XSelect CSH Fluoro-Phenyl prep column (250 mm x 10 mm, 5 μm) was utilized. Milli-Q water used in the experiments was sourced from an in-house Milli-Q system.

### Fungal materials

Six fungal materials used in this study: *A. ellipsoideum* (F1, F2), *A. synnematoferum* (F3, F4, F5), and *A. multiramosum* (F6) were isolated from driftwood collected in the intertidal zone of Troms County, Norway, in 2010. Details regarding their isolation, morphological description, and distribution in the phylogenetic tree were provided in Rämä et al. (2014) [19] and Hou et al. (2013) [12]. The fungal strains were cryopreserved in 20% sterile filtered glycerol at −80 °C at Marbio, UiT- the Arctic University of Norway. In this study, the cryostocks of fungi were thawed and plated onto Petri dishes containing malt extract agar (D2MAA) medium prepared in artificial sea water [4 g/L malt extract (Sigma-Aldrich), 40 g/L Instant Ocean Sea salts (Aquarium System), 15 g/L agar (A1296, Sigma-Aldrich), and Milli-Q^®^ H_2_O)]. Once the mycelium had grown over the plate, small pieces of agar containing fungal mycelium were excised and subcultured into a fresh Petri dish for further studies. To rule out the possibility of contamination, the identities of the subcultures were confirmed using internal transcribed spacer (ITS) sequencing as described previously [20].

### Metabolomics sample preparation

**Fig. 1:**
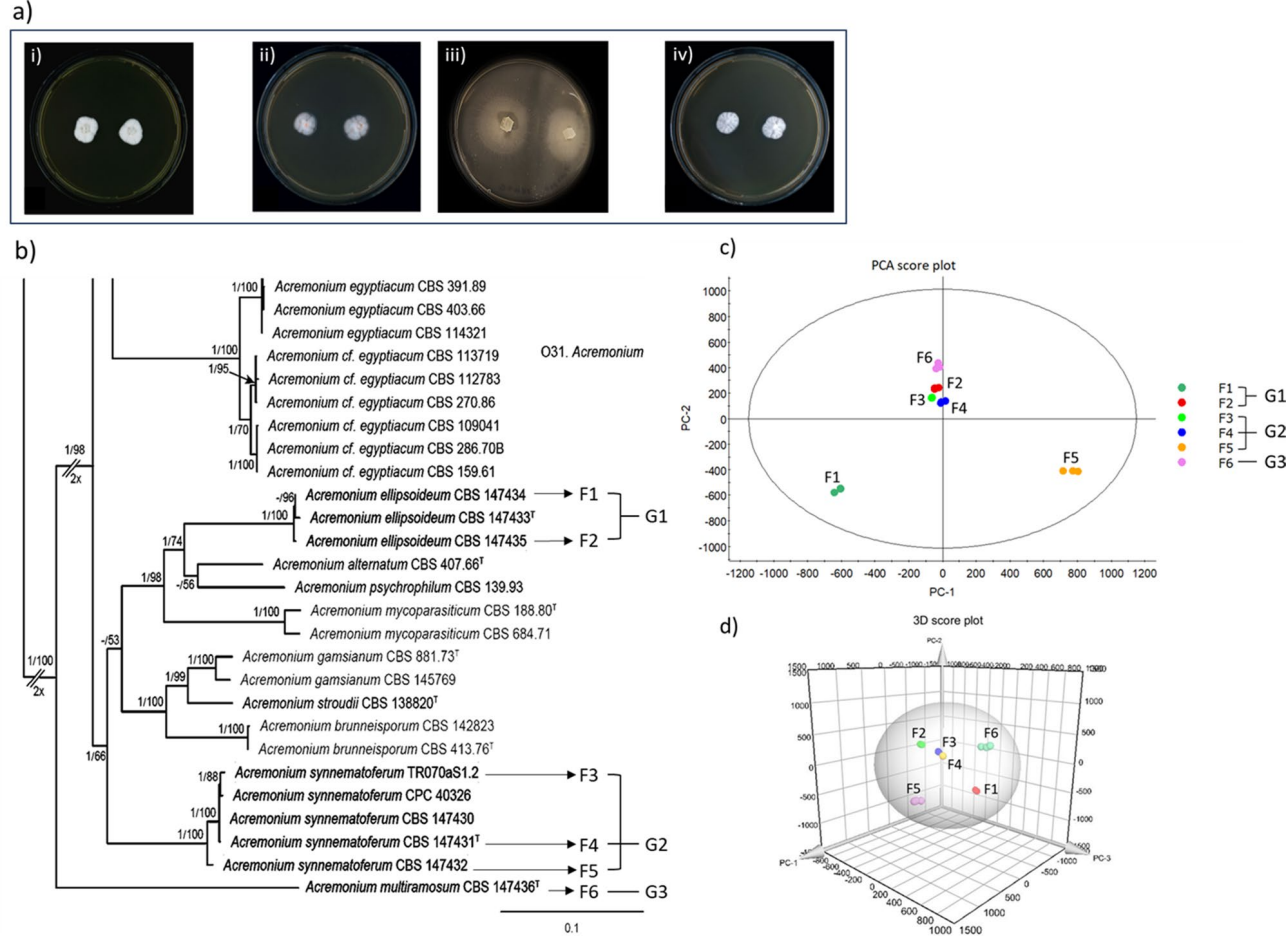
(**a**) Colonies of *Acremonium* strains: (i) F1 = CBS 147,433^T^ on malt extract agar medium (MA); (ii) F4 on MA; (iii) F5 on D2MAA; (iv) F6 on MA. (**b**) Phylogenetic tree showing six *Acremonium* strains, with their strain IDs and respective extract codes (F1-F6), included in this study. The images of colonies (except F5) and phylogenetic tree are sourced from Hou et al. (2023) [12]. (**c**) 2D (PCA) score plot of F1-F6. (**d**) 3D score plots of F1-F6. The extracts are grouped into three groups. F1 and F2 are grouped as G1 (*A. ellipsoideum*), F3-F5 as G2 (*A. synnematoferum*), and F6 as G3 (*A. multiramosum*)

Six freshly subcultured fungal strains on solid medium were excised into approximately 50 mm squares and transferred into individual culture flasks, each containing 250 mL of D2MA medium (composed of 4 g/L malt extract, 40 g/L Instant Ocean Sea salts, and Milli-Q^®^ H_2_O). After incubation for 35 days at 16 °C, the cultures were filtered through cheese cloths. The filtrated culture broths were then extracted using ethyl acetate with the solvent partitioning method. The ethyl acetate extracts were dried to produce six crude extracts (F1-F6) from the respective fungi, as shown in Fig. 1a, b. The extracts were dissolved in DMSO and subsequently diluted in methanol to prepare samples for metabolomics analysis. Samples were injected into UHPLC-HRMS^E^ in triplicate (8 µL) to facilitate chromatogram alignment during processing.

### Metabolomics data acquisition and processing for strain prioritization

The raw continuum HDMS^E^ data as UNIFI export package (*.uep) files were imported and processed using Progenesis QI (version 27.26.1020) software (Nonlinear Dynamics, Milford, MA, USA) with the following steps: profiling, peak alignment, lock mass calibration, experimental grouping, peak picking, deconvolution, identification, and statistical analysis. The samples were aligned based on an automatically selected reference alignment. Peak picking parameters were set to default values (automatic sensitivity threshold; no minimum chromatographic peak width), except for retention time, which was restricted to 1–12 min. Peak picking was updated by automatic peak processing, followed by setting up an experimental design. The compound ions generated were searched in the NPAtlas database for identification without any prior filtration step. The processed data were then exported to EZInfo 3.0 software (Waters Corp., Milford, USA) for multivariate statistical analyses. The data was scaled with Pareto normalization to perform an unsupervised principal component analysis (PCA) to visualize general clustering between all the sample runs. A supervised model, partial least-squares discriminant analysis (PLS-DA), was also utilized to visualize clustering between experimental groups. Finally, supervised orthogonal partial least-squares discriminant analysis (OPLS-DA) score plot and S-plot were used to validate the PCA model and identify marker compounds that correlated with the fungal extracts.

### Feature-based molecular networking analysis

The prioritized extract F5 was first processed using Progenesis QI. Peak picking parameters were set as follows: sensitivity threshold 20,000; minimum chromatographic peak width 0.1 min, retention time limits 2–10 min, fragment sensitivity 10% base peak, adducts included (M + H). The data were filtered using a precursor tolerance of 0.02 Da. Theoretical fragmentation was also prepared using a fragment tolerance of 0.02 Da and filtered by 90% isotope similarity. In total, 88 masses were detected, out of which 26 were identified.

The feature quantification table (.csv) and MS/MS spectral summary (.msp) were exported using the functions “export compound measurement” and “export fragment database”, respectively. These files were then uploaded to the GNPS platform (https://gnps.ucsd.edu) for feature-based molecular networking (FBMN) analysis [21]. Parameters for the molecular network generation were set as follows: both precursor mass and MS/MS fragment ion tolerance were set at 0.02 Da, minimum pairs cosine score 0.69, minimum matched fragment ions 3, minimum cluster size 2. The spectral library search was performed with a minimum matched peaks at 2, a score threshold at 0.5, and a filtered precursor window and peaks at 50 Da. The dereplicator was used to annotate MS/MS spectra [22]. The generated networks were visualized using Cytoscape (version 3.10.2) software [23].

### Bioactivity screening of extracts for strain prioritization

The six fungal extracts were screened for their bioactivity to prioritize the strains for upscaling fermentation and isolation of compounds. They were tested at 100 µg/mL for cytotoxic activity against THP-1 (acute monocytic leukemia), MCF7 (human breast adenocarcinoma) and A2058 (human melanoma) cell lines using a colorimetric viability assay using cell proliferation reagent (CellTiter 96^®^ Aqueous One Solution) that contains a tetrazolium compound [3-(4,5-dimethylthiazol-2-yl)-5-(3-carboxymethoxyphenyl)-2-(4-sulfophenyl)-2H-tetrazolium, inner salt; MTS] as previously described [24, 25].

### Upscale fermentation and extraction

The seed medium D2MA in 56 × 50 mL Falcon tubes (20 mL medium/flask) was inoculated with the fungal strain F5 by transferring a small piece of agar with fungal mycelium into each tube. The seed cultures were incubated at 16 °C for three weeks without shaking. Subsequently, 56 × 1000 mL culture flasks (containing 500 mL D2MA medium per flask) were inoculated with seed cultures. A large-scale fermentation (28 L in total) was then incubated at 16 °C for 60 days under static conditions, with occasional shaking (once per week). Finally, the fermentation broth was extracted with Diaion HP-20 (Supelco, 13607) resin and methanol to obtain crude extract. The extract was dried under reduced pressure in a rotary evaporator at 40 °C to obtain a dry crude extract.

### Compounds isolation

The extract was pre-fractionated using a Biotage SP4 Flash Chromatography System equipped with a self-packed Biotage SNAP 10 g cartridge column containing Diaion^®^ HP20SS (Supelco, 13615) resin as the stationary phase. Fractionation was performed with a stepwise gradient of H_2_O-MeOH (5:95, 25:75, 50:50, 75:25, 0:100; 6 min per step), followed by MeOH-Acetone (50:50 over 4 min, 0:100 over 10 min) at a flow rate of 12 mL/min, yielding eight fractions (Fr.1 to Fr.8). Fr. 5 was further fractionated using a preparative HPLC-MS with a Sunfire^®^ C18 OBD™ prep column (250 × 10 mm, 5 μm), using the following binary gradient with mobile phase A and B at a flow rate of 6 mL/min: initial isocratic composition of 53:47 (A: B) for 2 min, increasing linearly to 34:66 over 18 min, followed by an isocratic hold at 0:100 for 3 min, gradient returned to starting conditions 53:47 and held isocratic again for 2 min. In total, five subfractions (Fr.5 A−Fr.5E) were obtained. Subsequently, a second purification step was performed using chromatography on other columns as mentioned below, using the same mobile phase system at a flow rate of 6 mL/min.

Fr.5 A was further purified using XSelect^™^ CSH^™^ Phenyl-hexyl (PH) prep column (250 mm × 10 mm, 100 Å, 5 μm) using a step gradient elution of mobile phase A−B [66:34 (0–2 min), linearity to 58:42 (2–14 min), 0:100 (14.1–16 min), 66:34 (16–16.1 min) and held for 2 min] with a flow rate of 6 mL/min to yield 0.6 mg of **1**. Fr.5B was also further purified using the same PH column using a step gradient elution of mobile phase A−B [42% B (0–2 min), 43% B (2–11 min), 100% B (11.1–13 min), 42% B (13–13.1 min) and held for 3 min] with the same flow rate of 6 mL/min to afford 2 mg of **2**. Fr.5 C was obtained as a pure compound (2 mg) after the first round of isolation of **3** without the need for a purification step. Fr.5D was purified using PH column with a gradient elution of mobile phase A−B [57:43 (0–15 min), 0:100 (15.1–17 min), 57:43 (17.0–17.1 min), and held for 2 min] to afford 1.1 mg of **4**. Fr.5E was purified using an FP column with an isocratic elution of A: B (55:45) for 13 min, followed by a washing step 0:100 (13.1–15 min), and 55:45 (15.1–17) to afford 1 mg of **5**.

### Structure elucidation

The structures of the isolated compounds were elucidated through 1D and 2D NMR experiments. NMR spectra were recorded in DMSO-*d*_6_ at 25 °C using a Bruker AVANCE III HD spectrometer operating at 600 MHz, equipped with a cryogenically enhanced TCI probe. The resonance frequencies for ^1^H and ^13^C nuclei were 600 MHz and 150 MHz, respectively. Chemical shifts (δ) are reported in parts per million (ppm) using the residual solvent signal as reference.

### Cytotoxic activity of compounds **1** − **5**

Compounds **1** − **5** were tested for their cytotoxic effects against several cancer cell lines, including MOLM-13 (acute myeloid leukemia), MV-4-11 (biphenotypic B myelomonocytic leukemia), THP-1 (acute monocytic leukemia), A2058 (human melanoma), MCF7 (human breast adenocarcinoma), and non-cancerous MRC-5 (normal lung fibroblast) using an MTS based CellTiter 96^®^ AQ_ueous_ One Solution Assay [24, 25]. In short, the cells were cultured in appropriate media supplemented with 10% fetal bovine serum (FBS): MOLM-13, MV-4-11 cells in RPMI medium, THP-1 cells in RPMI medium with ultra-low endotoxin FBS, MCF7 and MRC-5 in MEM Eagle medium along with other amino acid supplements, and A2058 cells in DMEM medium. After harvesting, they were seeded into 96-well plates. The cells were then treated with the test compounds at various concentrations and incubated for 72 h. Following the incubation period, an MTS assay was conducted, and the absorbance of the solution was measured using a microplate reader spectrophotometer (Tecan spark plate reader) at a wavelength of 490 nm. The half-maximum inhibitory concentration (IC_50_) values were calculated using the *drc_3.0–1* package in R (Supplementary page S60-S61) [26, 27]. DMSO (10%) was used as the positive control, and cell culture media as the negative control. All samples were tested in triplicate.

### FLT3 kinase and PTP1B inhibitory activities of compounds **1** − **5**

FMS-like tyrosine kinase 3 wild type (FLT3 WT, PV3182, 2180555 F), Eu-anti-His antibody (PV5596), and fluorescent tracer 236 (PV5592) were purchased from ThermoFisher Scientific. Anti-GST-Eu-tagged monoclonal antibodies (anti-GST AB, 61GSTKLA) were purchased from Cisbio. The isolated compounds were evaluated for FLT3 WT kinase inhibitory effects using the FLT3 LanthaScreen^®^ activity assay protocol [28], with slight modifications and optimizations as previously described [29]. Briefly, compounds were dissolved in 20% DMSO, then diluted further in assay buffer A, and dispensed in triplicate into 384-well plates. Compounds were tested across 12 concentrations (0.006–12.6 µM), while quizartinib used as the positive control, was tested at concentrations ranging from 0.012 to 25.2 µM. FLT3-WT (30 nM) was mixed with anti-His antibody (6 nM). Tracer 236 (15 nM) was added, and the plate was incubated for 1 h at room temperature. Fluorescence measurements were taken using the EnVision 2104 multilabel reader (PerkinElmer, US), with excitation set to 340 nm, and emission read at both 615 nm (8.5 nm bandwidth) and 665 nm (7.5 nm bandwidth) over 200 ms, with a 100 ms delay between excitation and emission measurements. The emission ratio was calculated by dividing the signal at 665 nm by the signal at 615 nm for each compound and concentration. Emission ratios were normalized and plotted against compound concentration.

Human recombinant protein tyrosine phosphatase 1B (PTP1B) inhibitory activity of compounds **1**‒**5** was evaluated by measuring the enzyme activity using a 6,8-difluoro-4-methylumbelliferyl phosphate (DiFMUP) as a substrate. The assay was performed according to the method described by Montalibet et al. (2005) [30], with slight modifications of conditions as described in Hanssen et al. (2012) [31]. Briefly, PTP inhibitor IV (Merck-Calbiochem, 540211) was used as the positive control and assay buffer was used as the negative control. Compounds **1**‒**5** were tested at 100 µM concentration. Finally, the hydrolysis of DiFMUP by the PTP1B enzyme was measured by recording fluorescence at an excitation wavelength of 360 nm and an emission wavelength of 465 nm.

## Results

### UHPLC-HRMS-based metabolomics and chemometrics

The HDMS^E^ profile data from the six *Acremonium* fungal extracts (F1‒F6), processed using the Progenesis QI platform, generated a total of 16,825 compound ions (features) based on the selected threshold values. These features were used as variables for further chemometric analysis. PCA was applied to the Progenesis processed MS data to examine intrinsic variation in metabolite profiles among the extracts. This is an unsupervised multivariate data analysis that aims to reduce the dimensionality of data to reveal clusters, groups, and/or outliers among the observations. The PCA score plot (Fig. 1c) showed that the extracts F2, F3, F4, and F6 clustered together, whereas F1 and F5 clustered separately, indicating metabolite uniqueness of F1 and F5. To obtain further insight into the chemical similarity of clusters F2, F3, F4, and F6, a 3D score plot (PC-1 vs. PC-2 vs. PC-3) was also generated for visual depth analysis, and we found that the extracts F3 and F4 clustered together, while F2 and F6 were separated from each other (Fig. 1d). This result demonstrated differences between the F2, F6, and F3/F4 clusters.

**Fig. 2:**
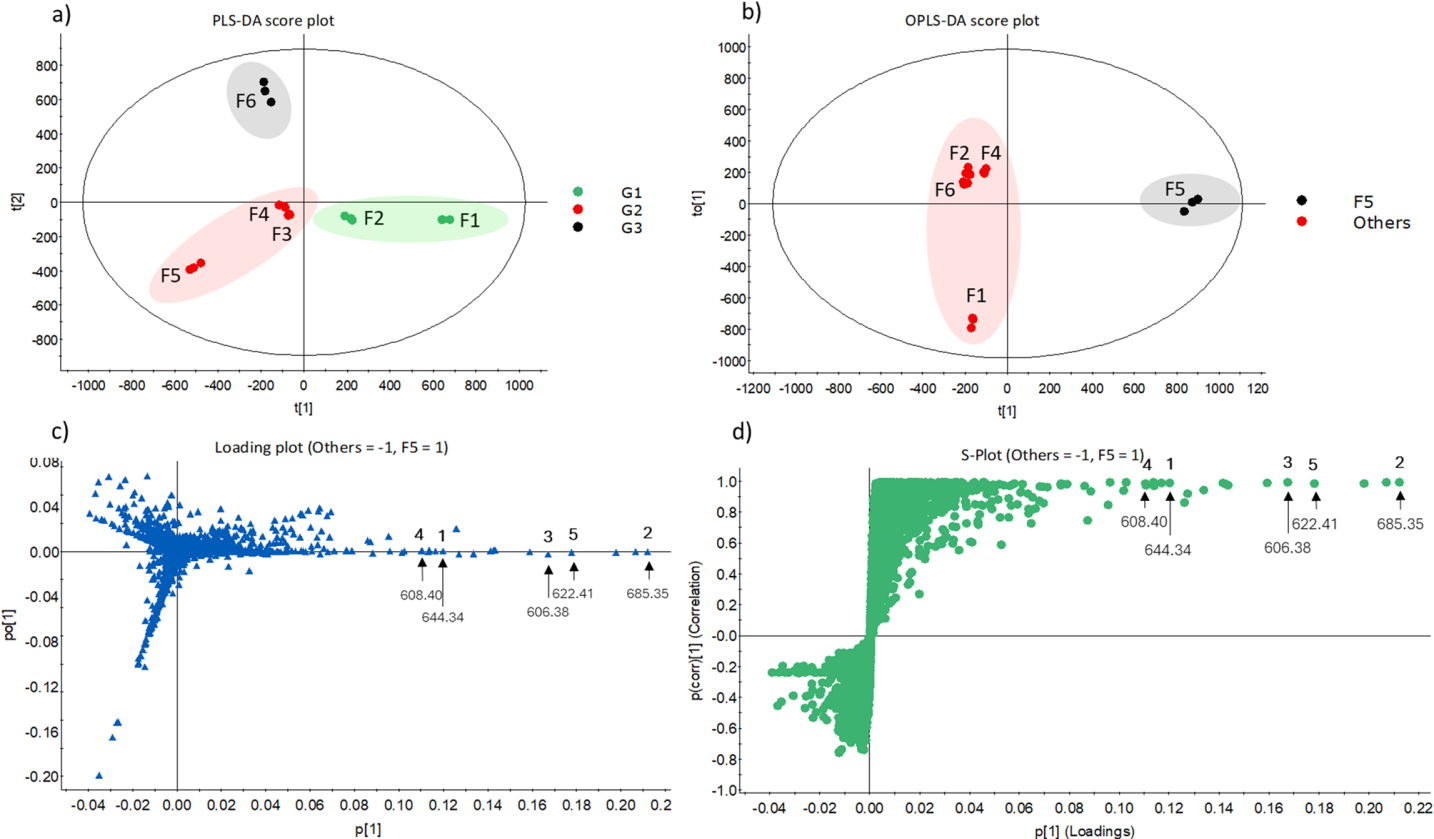
(**a**) PLS-DA score plot of six *Acremonium* fungal extracts (F1-F6) that are grouped into three experimental groups represented with different colours, i.e., G1 (green): *A. ellipsoideum*, G2 (red): *A. synnematoferum*, and G3 (black): *A. multiramosum*. (**b**) OPLS-DA score plot showing maximum separation of F5 and other fungal extracts. (**c**) Loading plot and (**d**) S-plot highlighting maker compounds that are unique to F5, which contribute to the group differences between F5 and other fungal extracts

To further discriminate among the three closely related taxonomic clades representing three species of fungi (G1: *A. ellipsoideum*, G2: *A. synnematoferum*, G3: *A. multiramosum*), the PLS-DA score plot was evaluated (Fig. 2a). It showed a dispersal of all three clades without any overlap between them, which indicates that these three taxonomic clades differ in their metabolite profiles. Notably, F3 and F4 of the G2 clade clustered very closely together and were close to F2 of the G1 clade, indicating that these extracts to a greater extent had similar metabolite profiles. In contrast, F6 of G3 was separated from G2 (particularly F3/F4), implying the presence of unique metabolites contributing to this variation (Fig. 2a). Therefore, among the extracts of the G2 clade (*A. synnematoferum*), F5 had a distinct metabolite profile. The quality of the PLS-DA model was evaluated using statistical model diagnostics R^2^Y (goodness of fit) and Q^2^ (predictability) parameters. In this case, the R^2^Y (cum) and Q^2^ (cum) were 0.77 and 0.69, respectively, demonstrating that the generated model had good reliability and predictability (Supplementary Fig. S3). To uncover highly discriminative marker compounds responsible for the differences among them, the data were grouped into two groups (F5 and others (F1-F4 and F6)), which were then compared using OPLS-DA (Fig. 2b) along with loading and S plots (Fig. 2c and d, respectively). In an S-plot, the X-axis denotes the contribution of marker compounds to the differences in grouping, whereas the Y-axis denotes the confidence of this contribution. The marker compounds in the upper right corner are characteristics of F5 and were significantly different from other extracts and are highlighted in Fig. 2c and d.

### Feature-based molecular networking analysis of F5

**Fig. 3:**
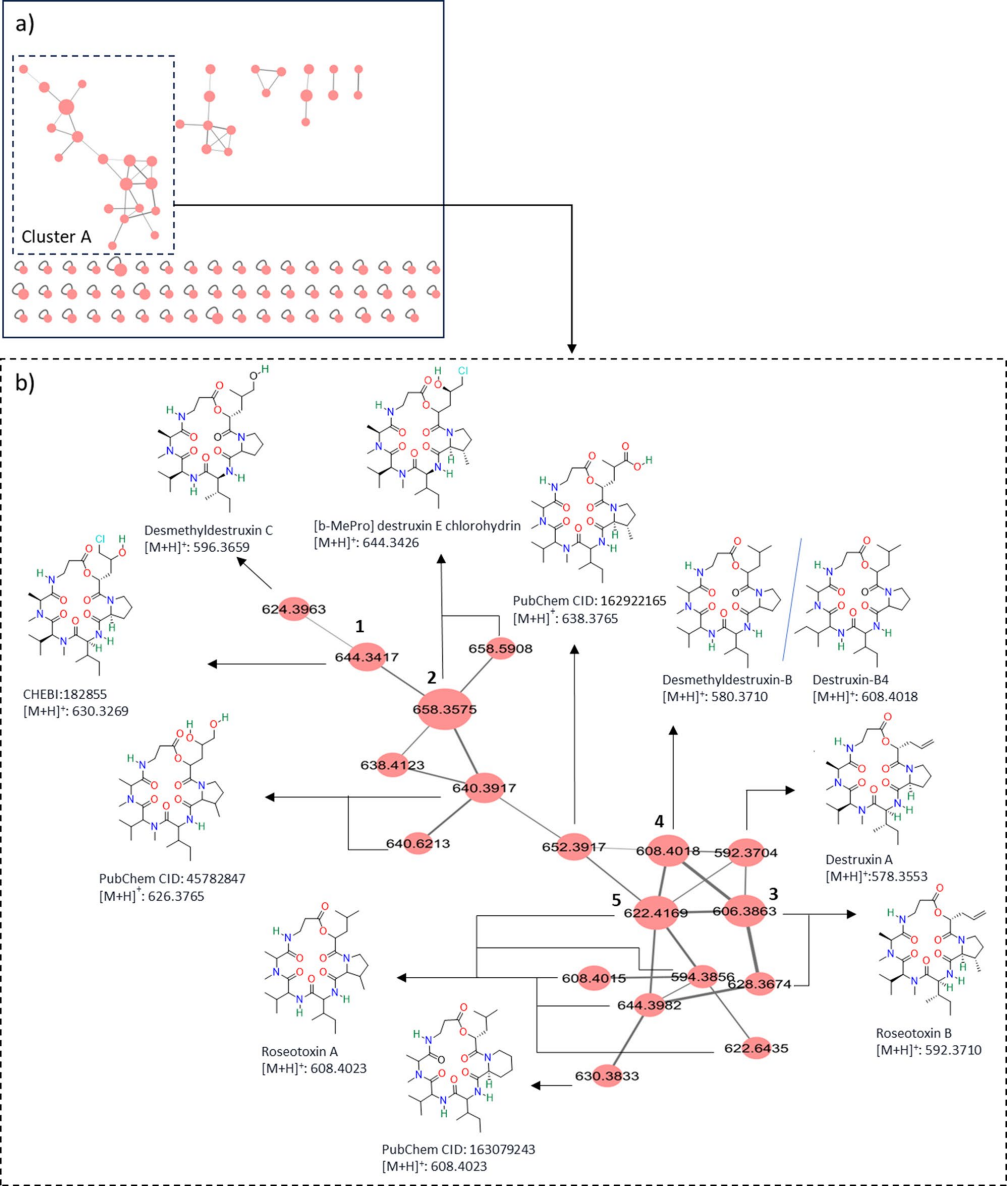
(**a**) Feature-based molecular networking of F5 highlighting cluster A that was identified as a peptide cluster. (**b**) Cluster A shows nodes that were annotated with their respective analog hits. Compounds **1**‒**5** were targeted for purification and subsequently isolated

To visualize molecular structural relationships and further annotate the marker compounds of F5, FBMN was used as an analysis method. The untargeted metabolomics (HDMS^E^) data of the F5 extract acquired by a data-independent acquisition were processed with Progenesis QI for metabolite feature detection. The resulting feature table was submitted to GNPS via FBMN workflow to generate a network. The molecular networking analysis of the F5 extract revealed that the marker features (compounds) with the highest priority identified in the discriminant analysis, which were shown in the S-plot, were grouped in cluster A, implying their structural similarity (Fig. 3). Cluster A consisted of 18 nodes, out of which only two nodes, with *m/z* 608.4018 and *m/z* 608.4015, were annotated as destruxin B4 and roseotoxin A, respectively. In addition, the GNPS library search identified several spectral matches to related analogs corresponding to the nodes within cluster A, as shown in Fig. 3b. An analog library hit refers to a spectral match between an experimental MS/MS spectrum and a similar, but not identical, spectrum in the GNPS spectral library. Since GNPS spectral search identified several peptides as analog hits, it provided strong confidence that these compounds belong to the peptide class of compounds.

### Bioactivity screening of fungal extracts

**Fig. 4:**
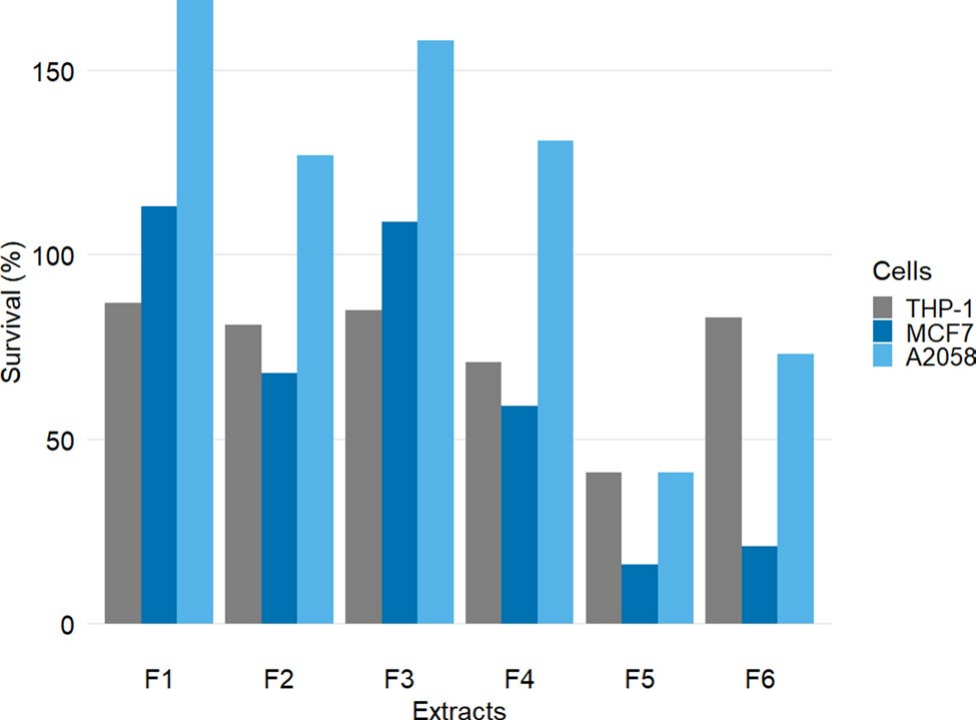
Cytotoxic activity of six extracts of *Acremonium* species against THP-1 (human monocyte), MCF7 (human breast carcinoma), and A2058 (human melanoma) cell lines tested at 100 µg/mL. The survival percentage was calculated from the average of three technical replicates

The results of the testing of the six fungal extracts for cytotoxic activity against three human cell lines, THP-1 (monocytic cell line), MCF7 (breast carcinoma), and A2058 (melanoma cell line), are shown in Fig. 4. Extracts F5 and F6 showed significant cytotoxic activity against the MCF7 cell line, with F5 being the only extract with significant activity against the THP-1 and A2058 cell lines. Notably, F5 demonstrated significant activity against all three cell lines. However, it demonstrated stronger cytotoxic activity against MCF7 cells (16% survival) compared to THP-1 and A2058 cells (41% survival). Therefore, the fungus F5 was prioritized for upscale fermentation to isolate and identify bioactive components.

### Isolation and identification of compounds

**Fig. 5:**
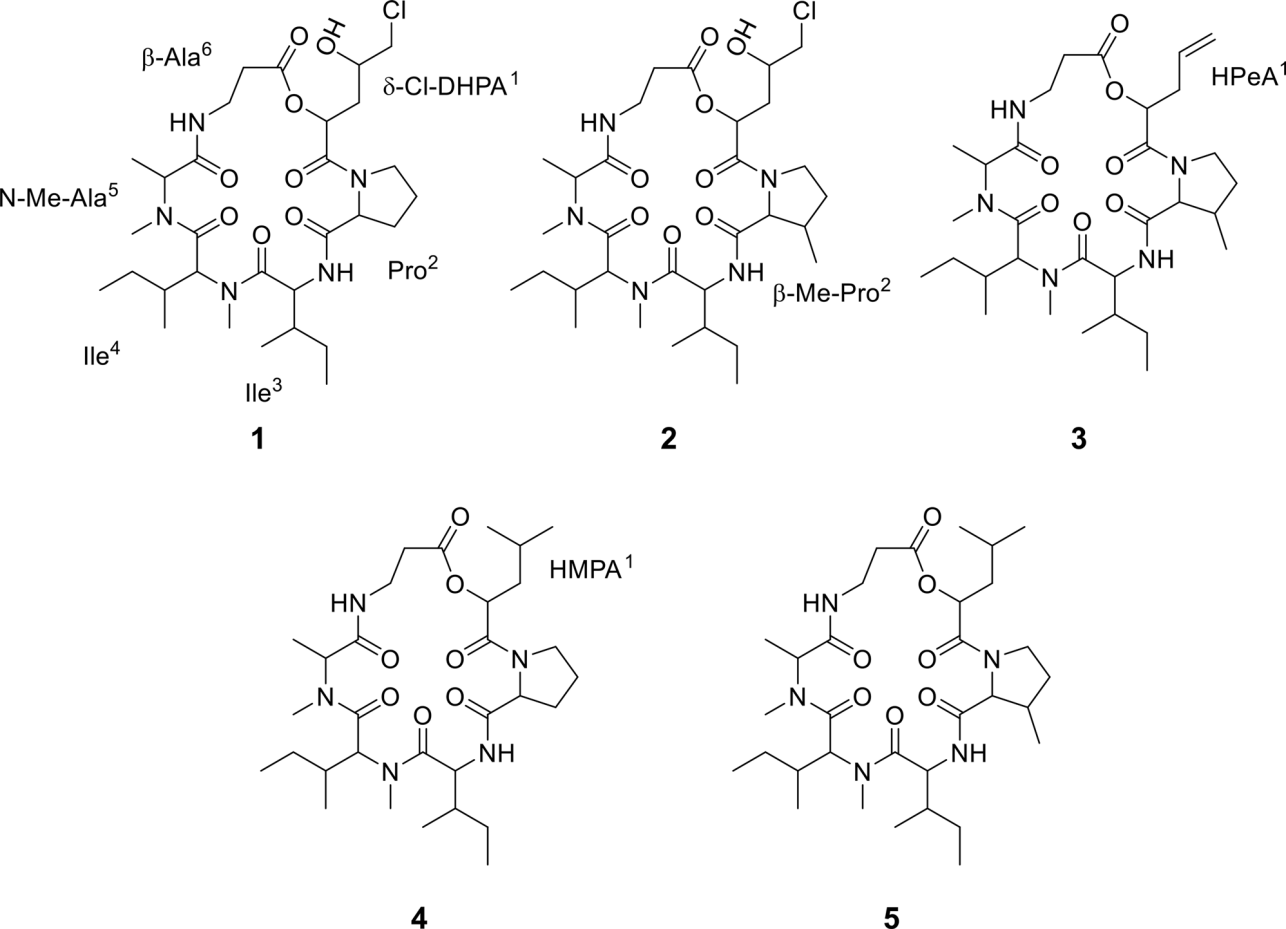
Structures of the isolated compounds **1**‒**5**

The upscaled fermentation of F5 yielded 18.2 g of dry crude extract, which was subsequently fractionated into eight fractions (Fr.1–8). The compounds, targeted for further studies based on the metabolomic analyses, were confirmed to be present in Fr.5. Compounds **1**‒**5** were isolated using mass-guided fractionation on preparative HPLC-MS and yielded 0.6, 2, 2, 1.1, and 1 mg, respectively. Their molecular structures were determined through comprehensive 1D and 2D NMR spectral analyses and identified as destruxin-A4 chlorohydrin (**1**) [32], trichomide D (**2**) [33], destruxin-A5 (**3**) [34], homodestruxin B (**4**) [34], and homodestcardin (**5**) [35]. Their structures are shown in Fig. 5, and the detailed assignments of ^1^H and ^13^C NMR signals are given in the supplementary information (Supplementary Table S1-S3). Additionally, we have also illustrated observed COSY and key HMBC correlations in Figure S96. All these compounds were depsipeptides that consisted of an α-hydroxy acid derivative, a β-alanine, and four amino acids. In particular, **1** consisted of δ-chloro-α,γ-dihydroxypentanoic acid (δ-Cl-DHPA^1^), β-Me-proline (β-Me-Pro^2^), isoleucine (Ile^3^), N-Me-isoleucine (N-Me-Ile^4^), N-Me-alanine (N-Me-Ala^5^), and β-alanine (β-Ala^6^) residues and sequenced as cyclo-(δ-Cl-DHPA^1^-β-Me-Pro^2^-Ile^3^-N-Me-Ile^4^-N-Me-Ala^5^-β-Ala^6^). Compound **2** had β-Me-Pro^2^ instead of the Pro^2^ found in **1**. The δ-Cl-DHPA^1^ residue in **1** was replaced with α-hydroxy-γ-pentenoic acid (HPeA^1^) and α-hydroxy-γ-methylpentanoic acid (HMPA^1^) in **3** and **4**, respectively. Compound **3** had β-Pro^2^ instead of β-Me-Pro^2^ in **4**. Compound **5** had N-proline (N-Pro^2^) instead of N-Me-Prol^2^ in **1**. This is the first time that these known cyclic depsipeptides have been isolated from this genus of fungi.

### Cytotoxic activity of **1**−**5**

Compounds **1**−**5** were evaluated for their cytotoxic activity against six human cell lines: haematological/leukemic cancer cell lines (THP-1, MOLM-13, MV-4-11), solid cancer cell lines (MCF7, A2058), and normal lung fibroblast cell line (MRC-5) with 10% DMSO in growth media as a positive control. Dose−response studies of the cytotoxic effects of **1**−**5** were performed at a concentration range varying between 0.002 and 80 µM (Supplementary Fig. S96). All the compounds inhibited the growth of all tested cell lines in a concentration-dependent manner. Table 1 shows the IC_50_ (the concentration required to inhibit 50% of cell growth) values of **1**−**5** against the various cell lines. Among the five compounds, **2** exhibited the most potent and broad-spectrum cytotoxicity, with submicromolar IC_50_ values against leukemic cells (MOLM-13 and MV-4-11), and remarkably low IC_50_ values (40 nM) against both the breast cancer cell line (MCF7) and the melanoma cell line (A2058). Compound **1** showed a similar potency profile, particularly against the leukemic cell lines (MOLM-13 and MV-4-11). It had IC_50_ of 0.8 µM against MCF7 and 1.1 µM against A2058 cell line. Compounds **3** and **5** demonstrated moderate activity across the cell lines included in the screening; the exception was for **3**, which had an IC_50_ of 15.2 µM against MRC-5, and **5** against THP-1 with an IC_50_ of 16.0 µM. Compound **4** displayed less potency across the cell lines, with higher IC_50_ values (5 to > 40 µM). The results also revealed that the IC_50_ values of the compounds were lowest for MOLM-13 compared to the other cell lines, except for **2** that were most active against MCF7 and A2058. All compounds exhibited cytotoxic activity against the tested cell lines, including normal lung fibroblast MRC-5, indicating a non-selective cytotoxic effect of these compounds. Further testing on a larger panel of cell lines is necessary to confirm their toxicity, and the molecular targets of these compounds should be identified.

**Table 1:** Cytotoxic activity of compounds **1**−**5** against six cell lines

	IC_50_ (µM)					
Compound	THP-1	MOLM-13	MV-4-11	MRC-5	MCF7	A2058
1	2.2	0.4	0.7	2.2	0.8	1.1
2	2.1	0.5	0.8	1.2	40.0*	40.0*
3	8.7	3.0	7.2	15.2	5.2	6.6
4	16.2	5.5	14.6	> 40.0	12.3	30.2
5	16.0	3.6	12.5	5.6	2.1	2.6

*: IC_50_ expressed in nanomolar (nM)

All five compounds were structurally very similar. They all shared β-Ala^6^ and three amino acids (Ile^3^-N-Me-Ile^4^-N-Me-Ala^5^), differing only in the α-hydroxy acid^1^ moiety and a second amino acid (β-Pro^2^ or β-Me-Pro^2^). A comparison of their structures and activities suggested that the presence of a δ-Cl-DHPA^1^ residue in **1** and **2** enhances cytotoxicity, as both exhibited potent effects. Moreover, the stronger activity of **2** compared to **1** against solid tumor cells may be due to the additional Me (CH_3_) group in β-Me-Pro^2^ in **2** compared to the Pro^2^ residue in **1**. Compound **3** contained an HPeA^1^ residue, while **4** contained an MHPA^1^ moiety instead of δ-Cl-DHPA^1^ in **1**. They did not show stronger cytotoxicity than **1** and **2** against these cells. The decreased cytotoxic effect of **3** and **4** might be attributed to the absence of a chlorine (δ-Cl) atom and a hydroxyl (γ-OH) group in their α-hydroxy acid^1^ derivative residues. Additionally, **3** had a lower IC_50_ across cell lines compared to **4**, which suggested that the Me group in the β-Me-Pro^2^ residue in **3** also contributed to the cytotoxic effect. Compound **5** exhibited higher activity compared to **4** across cell lines, especially against solid tumor cells, possibly due to the Me in the β-Me-Pro^2^ residue in **5**. These structure-activity relationship data indicated that the methylation of the Pro^2^ moiety and the presence of δ-Cl and γ-OH in the δ-Cl-DHPA residue of these cyclic depsipeptides are key structural features for potent cytotoxic activity.

### Enzyme inhibitory activity of **1**−**5**

Since compounds **1**−**5** exhibited cytotoxic effects against FLT3-driven acute myeloid leukemia cells such as MOLM-13 and MV-4-11, they were tested in a cell-free assay to evaluate FLT3 kinase inhibition. The FRET signal was measured to assess FLT3 inhibition by these compounds. None of the compounds inhibited FLT3 kinase at the test concentrations used (Supplementary Fig. S97), suggesting other possible mechanisms for their cytotoxicity. The compounds **1**‒**5** were also examined for their PTP1B inhibitory activity as they exhibited strong activity against the MCF7 cell line and PTP1B has been proposed in the literature as a potential target. However, compounds **1**‒**5** showed no activity at a concentration of 100 µM (Supplementary Fig. S98).

## Discussion

Dereplication of complex natural extracts remains a challenging task. While molecular weight and elemental composition predictions based on HRMS data can aid identification, they are often inconclusive. However, MS fragmentation patterns might provide specific features for certain compound classes [36]. These patterns are governed by their molecular structure and properties and serve as indicators of chemical relatedness, as related compounds exhibit similar fragmentation patterns. GNPS-based molecular networks organize MS/MS spectra based on fragment similarities and compare experimental MS/MS spectra against a public spectral library [37]. This helps to annotate unknown derivatives of known metabolites and also aids in identifying new molecular clusters.

MS-based untargeted metabolomics, integrated with chemometrics and molecular networking strategies, provided valuable insights into the metabolomes of phylogenetically related *Acremonium* fungi. Chemometric analyses enabled the discrimination of six *Acremonium* strains and allowed us to assess and identify distinct and shared metabolites between individual species and experimental groups. The results showed significant differences in metabolite composition between the groups (G1, G2, and G3). Among fungi within G2, F2 stands out because it has a distinct metabolite profile compared to F3 and F4. Metabolites found only in F2 are highlighted in the S-plot and loading plot. GNPS-FBMN was used to dereplicate and assess the fragment similarity of these marker compounds of F5. The resulting molecular networks indicated that they were structurally similar peptides. To isolate these peptides, large-scale fermentation of F5 was conducted using 500 mL medium per culture flask. The increased medium volume, compared to small-scale (250 mL/flask), provided sufficient nutrients to sustain fungal growth beyond 35 days. Consequently, the incubation period was extended to 60 days to maximize biomass production. The targeted compounds were then isolated and verified as structurally similar destruxin-type depsipeptides. All these compounds have previously been isolated from the fungus *Trichothecium roseum* (*Myrotheciomycetaceae*, *Hypocreales*) [32, 33, 38]. Additionally, they have been reported from other sources, including: **1** (destruxin-A4 chlorohydrin) from an undescribed mycelium isolated from leaf litter [32]; **3** (destruxin A5) and **4** (homodestruxin B) from an entomopathogenic fungus *Aschersonia* sp. [34]; **4** from a plant pathogenic fungus *Alternaria brassicaea* [39] and a sponge-derived fungus [35]; and **5** (homodestcardin) from an endophytic fungus *Fusarium chlamydosporum* and a sponge-derived fungus [33, 35]. However, this study represents the first report of these destruxin-family cyclic depsipeptides being isolated from *Acremonium* spp. (*Bionectriaceae*, *Hypocreales*), even though several *Acremonium* species are known to produce cyclic depsipeptides, cyclic hexa/hepta-peptides, and linear peptides [40–42]. The co-occurrence of multiple destruxin-family compounds in a single *Acremonium* strain (F5) is particularly noteworthy. These destruxin compounds are composed of an α-hydroxy acid and five amino acid residues and are commonly referred to as cyclic hexadepsipeptides.

This study provides a comparison of the cytotoxic effects of **1**–**5** across various cell lines, categorized into leukemic, solid tumor, and normal fibroblast cells. Notably, all compounds exhibited cytotoxic effects against all tested cell lines, with **2** being the most potent and exhibiting an IC_50_ value of 40 nM against MCF7 and A2058 cell lines. The IC_50_ of **2** is comparable to previously reported data (IC_50_ of 79 nM) for the MCF7 cell line. Among the tested compounds, only **2** has been previously reported to exhibit cytotoxic activity against MCF7, HL-60 (acute promyelocytic leukemia), and SW480 (human colon carcinoma) cells with IC_50_ values of 0.079, 0.107, and 0.149 µM respectively [33]. Given that HL-60 is a subtype of acute myeloid leukemia, the IC_50_ values of **2** against HL-60 (0.149 µM) and MOLM-13 (0.5 µM) are expected to be within a similar range, and indeed, they show slight differences. Importantly, besides **2**, this is the first report of cytotoxic effect of the other four compounds (**1**, **3**–**5**) against the tested cell lines. The result further revealed that the compounds exhibited cytotoxic activity against both subtypes of myeloid leukemia cells, MV-4-11 and MOLM-13, which are highly dependent on FLT3 signalling for survival and proliferation. Nevertheless, none of the compounds showed inhibitory effects on FLT3 kinase. This indicated that their cytotoxic effects operate through mechanisms independent of FLT3 signalling. This conclusion was further supported by the observed cytotoxic effects of the compounds on THP-1 cells, which lack the FLT3-ITD mutation and do not rely on FLT3 signalling for growth or survival. Moreover, compound **3** is known to selectively inhibit PDGF-BB/PDGFR-ββ signalling, thereby attenuating liver fibrosis [38]. The compound’s selective inhibition of PDGFR, with no activity on FLT3, highlights its potential as a targeted therapy for PDGFR-driven cancers: such as non-small cell lung cancers, gastrointestinal stromal tumors, and ovarian cancers. Since compounds **1**–**5** exhibited cytotoxic activity against breast cancer cells (MCF7), their inhibitory activity on PTP1B was also evaluated. PTP1B, in addition to being a potential drug target for treating diabetes and obesity due to its role in downregulating insulin and leptin signalling, has also emerged as a potential therapeutic target for breast cancer treatment [43]. This is due to its role in promoting HER2/Neu (ErbB2)-mediated signalling that promotes tumor cell proliferation [44]. Considering the cytotoxicity of **1**–**5** across a panel of tested cell lines suggests that toxicity may be due to broad-spectrum mechanisms such as causing DNA damage, mitochondrial disruption, or disrupting membrane integrity [45]. Since other compounds structurally similar to destruxins have been reported to inhibit the phosphoinositide-3-kinase (PI3K)/Akt pathway and are associated with intracellular redox balance, it is worth exploring similar cytotoxic mechanisms for destruxins [33, 46, 47]. While the cytotoxicity observed against both cancer and normal (MRC-5) cell lines could be considered limiting the immediate therapeutic application, these compounds could rather act as a starting point for synthesizing analogs to evaluate structure-activity relationship. The cytotoxicity could then be addressed to improve the selectivity of the identified compounds. In addition to cytotoxic effects, the destruxin-type compounds are also known for other biological functions: compounds **1**−**5** have shown lethal activity against brine shrimp and nematocidal activity [33]; **3** has exhibited entomopathogenic activity [34]; **4** has shown phytotoxic activity [48]; and **1** has induced erythropoietin gene expression [32].

## Conclusions

The main aim of this work was to analyze the metabolic profiles of six *Acremonium* strains using MS-based metabolomics to prioritize for further identification of bioactive metabolites. Combining metabolomics, chemometrics, and bioactivity data proved to be effective and highly useful for prioritizing fungal species for isolating potential bioactive secondary metabolites. Our study emphasized the necessity of a multi-informative approach for prioritizing microbial strains for natural product identification. Furthermore, our results on the bioactivity of the isolated destruxin-type depsipeptides exhibited broad cytotoxicity across the tested cell lines. While this limits their immediate therapeutic application, the potent activities highlight these scaffolds as valuable leads for structure-activity relationship studies aimed at improving selectivity. Further studies should focus on elucidating the mode of action of these destruxin-type depsipeptides and optimizing their selectivity once the target is identified. Additionally, further research should investigate the distribution of biosynthetic gene clusters (BGCs) responsible for destruxin-type depsipeptide production across *Acremonium* species to unlock their full potential.

## Supplementary Information

Below is the link to the electronic supplementary material.

Supplementary Material 1

## Data Availability

All data generated or analyzed during this study are included in this published article and its supplementary information file. Supplementary information includes metabolomics and chemometric data, preparatory HPLC-MS chromatograms, HRESIMS spectra, 1D and 2D NMR spectra of the compounds, and bioactivity data.
