# Supplementary material for: Marine-derived Acremonium strain prioritization using untargeted metabolomics approach for the identification of cytotoxic cyclic depsipeptides
Source: BMC Biotechnol. 2025 Nov 24;25:129. doi: 10.1186/s12896-025-01065-2 (PMC12642047; doi:10.1186/s12896-025-01065-2)
Supplement: Supplementary file 1 — Supplementary Material 1 [file 12896_2025_1065_MOESM1_ESM.docx]

**Supporting Information**

Marine-derived *Acremonium* strain prioritization using untargeted Metabolomics approach for the identification of cytotoxic cyclic depsipeptides

Sailesh Maharjan^1^*, Johan Isaksson^2, 3^, Teppo Rämä^1^, Kine Østnes Hansen^2^, Jeanette Hammer Andersen^1^, Espen Holst Hansen^1^

^1^ Marbio, Faculty of Biosciences, Fisheries, and Economics, UiT-The Arctic University of Norway, Tromsø, Norway

^2^ Department of Pharmacy (IFA), Faculty of Health Sciences, UiT-The Arctic University of Norway, Tromsø, Norway

^3^Department of Chemistry (IK), Faculty of Science and Technology, UiT-The Arctic University of Norway, Tromsø, Norway

*** Corresponding authors:**

Sailesh Maharjan: Phone: +4796804941; Email: [sailesh.maharjan@uit.no](mailto:sailesh.maharjan@uit.no)

**List of Figures**

[Figure S 1. Model diagnostic: goodness of fit for the PCA score plot of six fungal extracts (F1-F6) 5](#_Toc209896449)

[Figure S 2. Model diagnostic: Hotelling’s T2 range for the PCA score plot of UHPLC-HR-MS data from six *Acremonium* fungal extracts (F1-F6) 6](#_Toc209896450)

[Figure S 3. Model diagnostic: Model diagnostic: goodness of fit for the PLS-DA score plot of UHPLC-HR-MS data from six *Acremonium* fungal extracts (F1-F6) 7](#_Toc209896451)

[Figure S 4. Model diagnostic: Hotelling’s T2 range for the PLS-DA score plot of UHPLC-HR-MS data from six *Acremonium* fungal extracts (F1-F6) 7](#_Toc209896452)

[Figure S 5. BPI Chromatogram of the first round of purification of Fr.5, resulting in subfractions (Fr.5A‒Fr.5E). 8](#_Toc209896453)

[Figure S 6. BPI Chromatogram of the second round of purification of Fr.5A, resulting in a pure compound **1**. 8](#_Toc209896454)

[Figure S 7. BPI Chromatogram of the second round of purification of Fr.5B, resulting in a pure compound **2**. 9](#_Toc209896455)

[Figure S 8. BPI Chromatogram of Fr.5C. After the first round of purification, it was obtained as a pure compound and named as **3**, without the need for an additional purification step. 9](#_Toc209896456)

[Figure S 9. BPI Chromatogram of the second round of purification of Fr.5D, resulting in a pure compound **4**. 10](#_Toc209896457)

[Figure S 10. BPI Chromatogram of the second round of purification of Fr.5E, resulting in a pure compound **5**. 10](#_Toc209896458)

[Figure S 11. HRESIMS of **1** at (a) low collision energy and (b) high collision energy in ESI+, HDMS^E^ mode. (c) Fragmentation of **1**. 11](#_Toc209896459)

[Figure S 12. ^1^H NMR spectrum of **1**. 12](#_Toc209896460)

[Figure S 13. ^13^C NMR spectrum of **1**. 12](#_Toc209896461)

[Figure S 14. HSQC spectrum of **1**. 13](#_Toc209896462)

[Figure S 15. ^1^H, ^15^N-HSQC spectrum of **1**. 13](#_Toc209896463)

[Figure S 16. ^1^H,^15^N-HSQC (Expanded) spectrum of **1**. 14](#_Toc209896464)

[Figure S 17. ^1^H, ^15^N-HMBC spectrum of **1**. 14](#_Toc209896465)

[Figure S 18. ^1^H,^15^N-HMBC (Expanded) spectrum of **1**. 15](#_Toc209896466)

[Figure S 19. HMBC spectrum of **1**. 15](#_Toc209896467)

[Figure S 20. HMBC (8Hz long range) spectrum of **1**. 16](#_Toc209896468)

[Figure S 21. H2BC spectrum of **1**. 16](#_Toc209896469)

[Figure S 22. DQF-COSY spectrum of **1**. 17](#_Toc209896470)

[Figure S 23. TOCSY ((DIPSI2) 60ms) spectrum of **1**. 17](#_Toc209896471)

[Figure S 24. ROESY (300 ms) spectrum of **1**. 18](#_Toc209896472)

[Figure S 25. HRESIMS of **2** at (a) low collision energy and (b) high collision energy in ESI+, HDMS^E^ mode. (c) Fragmentation of **2**. 19](#_Toc209896473)

[Figure S 26. ^1^H NMR spectrum of **2**. 20](#_Toc209896474)

[Figure S 27. ^1^H NMR (Expanded) spectrum of **2**. 20](#_Toc209896475)

[Figure S 28. ^13^C NMR spectrum of **2**. 21](#_Toc209896476)

[Figure S 29. ^13^C NMR (Expanded) spectrum of **2**. 21](#_Toc209896477)

[Figure S 30. HSQC spectrum of **2**. 22](#_Toc209896478)

[Figure S 31. HSQC (Expanded) spectrum of **2**. 22](#_Toc209896479)

[Figure S 32. HMBC spectrum of **2**. 23](#_Toc209896480)

[Figure S 33. H2BC spectrum of **2**. 23](#_Toc209896481)

[Figure S 34. H2BC (5 Hz long range) spectrum of **2**. 24](#_Toc209896482)

[Figure S 35. ^1^H,^15^N-HSQC spectrum of **2**. 24](#_Toc209896483)

[Figure S 36. ^1^H,^15^N-HSQC (Expanded) spectrum of **2**. 25](#_Toc209896484)

[Figure S 37. DQF-COSY spectrum of **2**. 25](#_Toc209896485)

[Figure S 38. TCOSY ((DIPSI2) 60ms) spectrum of **2**. 26](#_Toc209896486)

[Figure S 39. ROESY (300 ms) spectrum of **2**. 26](#_Toc209896487)

[Figure S 40. HRESIMS of **3** at (a) low collision energy and (b) high collision energy in ESI+, HDMS^E^ mode. Fragmentation of **3**. 27](#_Toc209896488)

[Figure S 41. ^1^H NMR spectrum of **3**. 28](#_Toc209896489)

[Figure S 42. ^1^H NMR (Expanded) spectrum of **3**. 28](#_Toc209896490)

[Figure S 43. ^13^C NMR spectrum of **3**. 29](#_Toc209896491)

[Figure S 44. ^13^C NMR (Expanded) spectrum of **3**. 29](#_Toc209896492)

[Figure S 45. HSQC spectrum of **3**. 30](#_Toc209896493)

[Figure S 46. HSQC (Expanded) spectrum of **3**. 30](#_Toc209896494)

[Figure S 47. HMBC of spectrum **3**. 31](#_Toc209896495)

[Figure S 48. HMBC (Expanded1) spectrum of **3**. 31](#_Toc209896496)

[Figure S 49. HMBC (Expanded2) spectrum of **3**. 32](#_Toc209896497)

[Figure S 50. Selective HMBC (8 Hz) spectrum of **3**. 32](#_Toc209896498)

[Figure S 51. H2BC spectrum of **3**. 33](#_Toc209896499)

[Figure S 52. ^1^H,^15^N-HSQC spectrum of **3**. 33](#_Toc209896500)

[Figure S 53. ^1^H,^15^N-HSQC (Expanded) spectrum of **3**. 34](#_Toc209896501)

[Figure S 54. COSY spectrum of **3**. 34](#_Toc209896502)

[Figure S 55. ROESY (300 ms) spectrum of **3**. 35](#_Toc209896503)

[Figure S 56. ROESY (Expanded) spectrum of **3**. 35](#_Toc209896504)

[Figure S 57. HRESIMS of **4** at (a) low collision energy and (b) high collision energy in ESI+, HDMS^E^ mode. Fragmentation of **4**. 36](#_Toc209896505)

[Figure S 58. ^1^H NMR spectrum of **4**. 37](#_Toc209896506)

[Figure S 59. ^1^H NMR (Expanded) spectrum of **4**. 37](#_Toc209896507)

[Figure S 60. ^13^C NMR spectrum of **4**. 38](#_Toc209896508)

[Figure S 61. ^13^C NMR (Expanded) spectrum of **4**. 38](#_Toc209896509)

[Figure S 62. HSQC spectrum of **4**. 39](#_Toc209896510)

[Figure S 63. HSQC (Expanded) spectrum of **4**. 39](#_Toc209896511)

[Figure S 64. ^1^H,^15^N-HSQC spectrum of **4**. 40](#_Toc209896512)

[Figure S 65. ^1^H,^15^N-HSQC (Expanded) spectrum of **4**. 40](#_Toc209896513)

[Figure S 66. HMBC spectrum of **4**. 41](#_Toc209896514)

[Figure S 67. HMBC (Expanded) spectrum of **4**. 41](#_Toc209896515)

[Figure S 68. Selective HMBC (8 Hz) spectrum of **4**. 42](#_Toc209896516)

[Figure S 69. Selective HMBC (Expanded) spectrum of **4**. 42](#_Toc209896517)

[Figure S 70. Selective HMBC (8 Hz) spectrum of **4**. 43](#_Toc209896518)

[Figure S 71. H2BC spectrum of **4**. 43](#_Toc209896519)

[Figure S 72. H2BC (Expanded) spectrum of **4**. 44](#_Toc209896520)

[Figure S 73. COSY spectrum of **4**. 44](#_Toc209896521)

[Figure S 74. COSY (Expanded) spectrum of **4**. 45](#_Toc209896522)

[Figure S 75. ROESY spectrum of **4**. 45](#_Toc209896523)

[Figure S 76. ROESY spectrum of **4**. 46](#_Toc209896524)

[Figure S 77. HRESIMS of **5** at (a) low collision energy and (b) high collision energy in ESI+, HDMS^E^ mode. Fragmentation of **5**. 47](#_Toc209896525)

[Figure S 78. ^1^H NMR spectrum of **5**. 48](#_Toc209896526)

[Figure S 79. ^1^H NMR (Expanded) spectrum of **5**. 48](#_Toc209896527)

[Figure S 80. ^13^C NMR spectrum of **5**. 49](#_Toc209896528)

[Figure S 81. ^13^C NMR (Expanded) spectrum of **5**. 49](#_Toc209896529)

[Figure S 82. HSQC spectrum of **5**. 50](#_Toc209896530)

[Figure S 83. ^1^H,^15^N-HSQC spectrum of **5**. 50](#_Toc209896531)

[Figure S 84. ^1^H,^15^N-HSQC (Expanded) spectrum of **5**. 51](#_Toc209896532)

[Figure S 85. HMBC spectrum of **5**. 51](#_Toc209896533)

[Figure S 86. HMBC (Expanded1) spectrum of **5**. 52](#_Toc209896534)

[Figure S 87. HMBC (Expanded2) spectrum of **5**. 52](#_Toc209896535)

[Figure S 88. H2BC spectrum of **5**. 53](#_Toc209896536)

[Figure S 89. H2BC (Expanded) spectrum of **5**. 53](#_Toc209896537)

[Figure S 90. ^1^H,^15^N-HMBC spectrum of **5**. 54](#_Toc209896538)

[Figure S 91. Selective HMBC (8 Hz) spectrum of **5**. 54](#_Toc209896539)

[Figure S 92. COSY spectrum of **5**. 55](#_Toc209896540)

[Figure S 93. COSY (Expanded) spectrum of **5**. 55](#_Toc209896541)

[Figure S 94. ROESY spectrum of **5**. 56](#_Toc209896542)

[Figure S 95. ROESY (Expanded) spectrum of **5**. 56](#_Toc209896543)

[Figure S 96. The key HMBC (blue arrows) and ^1^H-^1^H COSY (blue bond lines) correlations of **1**–**5**. 60](#_Toc209896544)

[Figure S 97. Dose-response curve showing the survival percentage of (a) THP-1, (b) MOLM-13, (c) MV-4-11, (d) MCF7, and (e) A2058 cells treated with compounds **1**‒**5** at different concentrations. The survival % were measured using MTS assay. Data represent the mean values from three technical replicates. 61](#_Toc209896545)

[Figure S 98. Dose-response (DR) curves for the FLT3 WT enzyme inhibitory activity of quizartinib and compounds **1**‒**5**. The Curves were obtained by plotting the graph between log_10_ concentrations of the compounds (nM) and emission ratios (Y-axis). Error bars represent the standard deviation of three technical replicates. Compounds **1**‒**5** did not inhibit FLT3 WT at the concentration range tested (12 nM‒50 µM). 62](#_Toc209896546)

[Figure S 99. Residual PTP1B enzyme activity of **1**‒**5**. The compounds were tested at 100 µM with two technical replicates. None of the compounds were active, as PTP1B enzyme activity of all the compounds was above 75%. The enzyme activity should be below 30% to be considered active. 63](#_Toc209896547)

**List of Tables**

[Table S 1. ^1^H (600 MHz) and ^13^C (151 MHz) NMR data of **1** and **2** in DMSO-*d_6_*. 57](#_Toc209896548)

[Table S 2. ^1^H (600 MHz) and ^13^C (151 MHz) NMR data of **3** in DMSO-*d_6_*. 58](#_Toc209896549)

[Table S 3. ^1^H (600 MHz) and ^13^C (151 MHz) NMR data of **4** and **5** in DMSO-*d_6_*. 59](#_Toc209896550)

**Model diagnostics for chemometrics**

**PCA Diagnostics: Evaluation of R^2^ and Q^2^**

In the PCA plot, R²Y(cum) represents the proportion of variance in the Y matrix (response matrix) explained by the model, indicating how well the model fits the data. Q²(cum) represents the proportion of variance in the Y matrix that can be predicted by the model through cross-validation, reflecting its predictive accuracy.

The values of R²Y and Q² were approximately 0.6 and 0.4, respectively, meaning the two components together explain 60% of the variance in Y and predict 40% of variance. This suggests that the model is stable and reliable. Ideally, R² should be slightly larger than Q², with the difference not being too large to avoid overfitting. In metabolomics or when analyzing complex mixtures, R²Y and Q² values greater than or equal to 0.5 are generally considered acceptable for a good model. In this case, the PCA plot (PC1 vs. PC2) with R²Y ≈ 0.6 and Q² ≈ 0.4 indicates a moderately good model. While the model is not overfitting, its predictive ability could be improved. Adding more components might enhance R² and Q², but it is not strictly necessary in this case.


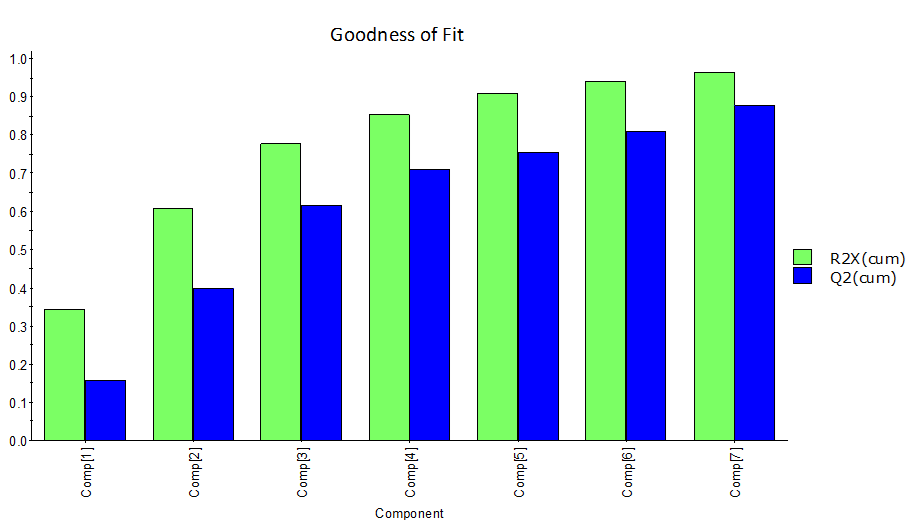


Figure S 1. Model diagnostic: goodness of fit for the PCA score plot of six fungal extracts (F1-F6)

**PCA Diagnostics: Hotelling’s T2 Range**

In the PCA plot, strong outliers are easy to identify but are significant because they often have a substantial influence on the model. Hotelling’s T² is a multivariate generalization of Student’s t-distribution. If the data is normally distributed, 95% of the observations fall inside the range, while 5% fall outside. It visually emphasizes deviations from normality. Hotelling’s T² statistic measures how far a given observation is from the center (mean) of the model in the multivariate space defined by the principal components. The larger the distance, the more extreme the observation. In this analysis, all T² values for the samples were below the T² Crit (95%) value of 32.59, indicating that they are within the normal range.


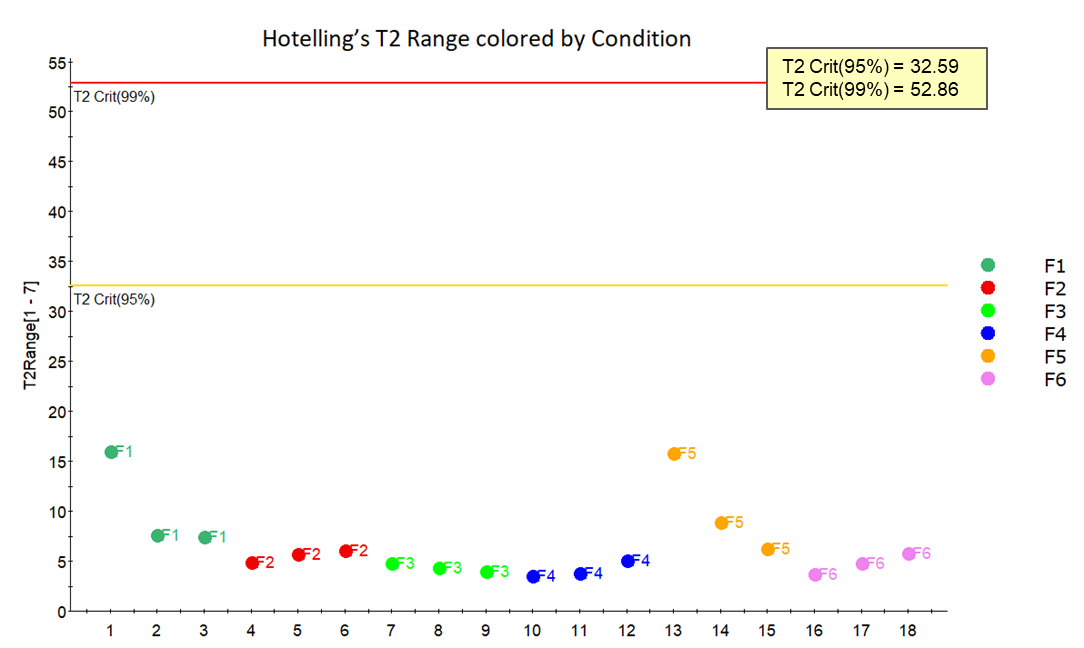


Figure S 2. Model diagnostic: Hotelling’s T2 range for the PCA score plot of UHPLC-HR-MS data from six *Acremonium* fungal extracts (F1-F6)

**PLS-DA Diagnostics: Evaluation of R^2^ and Q^2^**

In PLS-DA plot (PC1 vs. PC2), the goodness of fit with R²Y ≈ 0.75 and Q² ≈ 0.7 indicates a good/stable and reliable model. It means two components together explain 75% of the variance in Y and predicts 70% of variation.


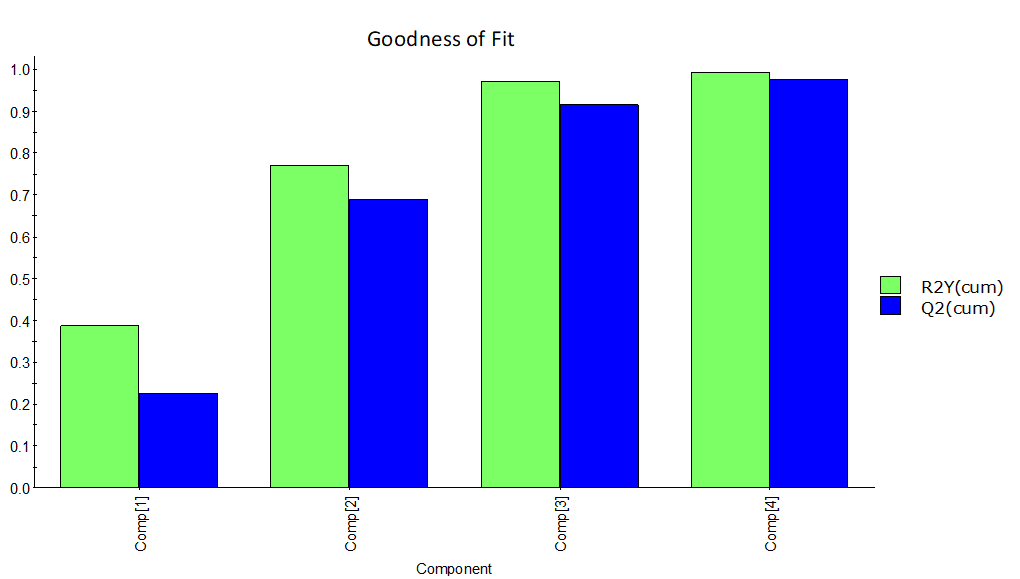


Figure S 3. Model diagnostic: Model diagnostic: goodness of fit for the PLS-DA score plot of UHPLC-HR-MS data from six *Acremonium* fungal extracts (F1-F6)

**PCA Diagnostics: Hotelling’s T2 Range**

In this analysis, all T² values for the samples were below the T² Crit (95%) value of 15.12, indicating that they are within the normal range.


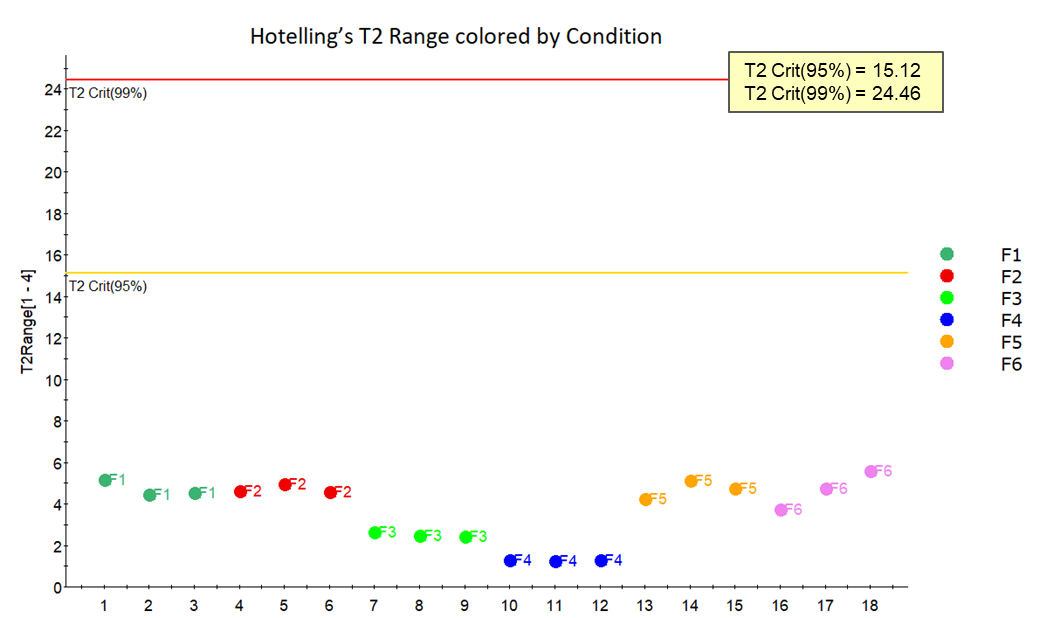


Figure S 4. Model diagnostic: Hotelling’s T2 range for the PLS-DA score plot of UHPLC-HR-MS data from six *Acremonium* fungal extracts (F1-F6)

**Isolation of Compounds**

**First Round of Purification**


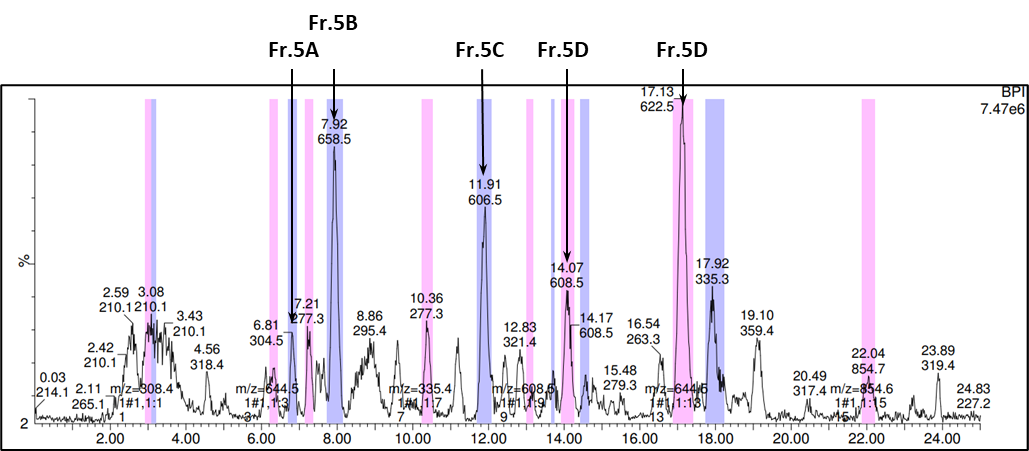


Figure S 5. BPI Chromatogram of the first round of purification of Fr.5, resulting in subfractions (Fr.5A‒Fr.5E).

**Second Round of Purification**


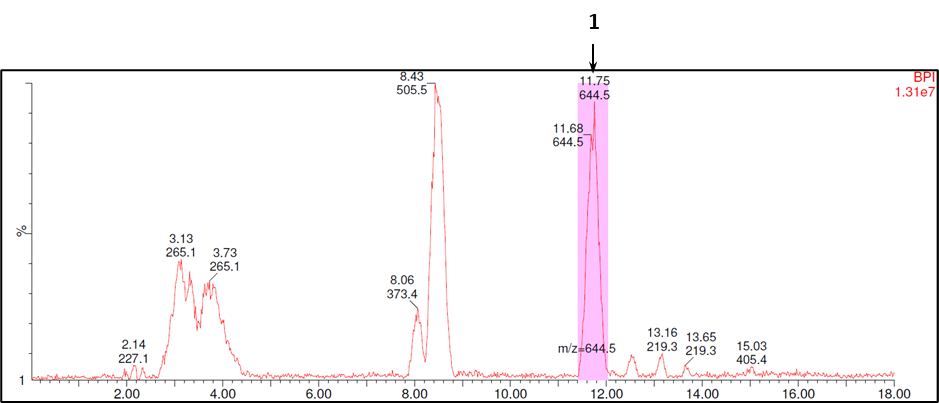


Figure S 6. BPI Chromatogram of the second round of purification of Fr.5A, resulting in a pure compound **1**.


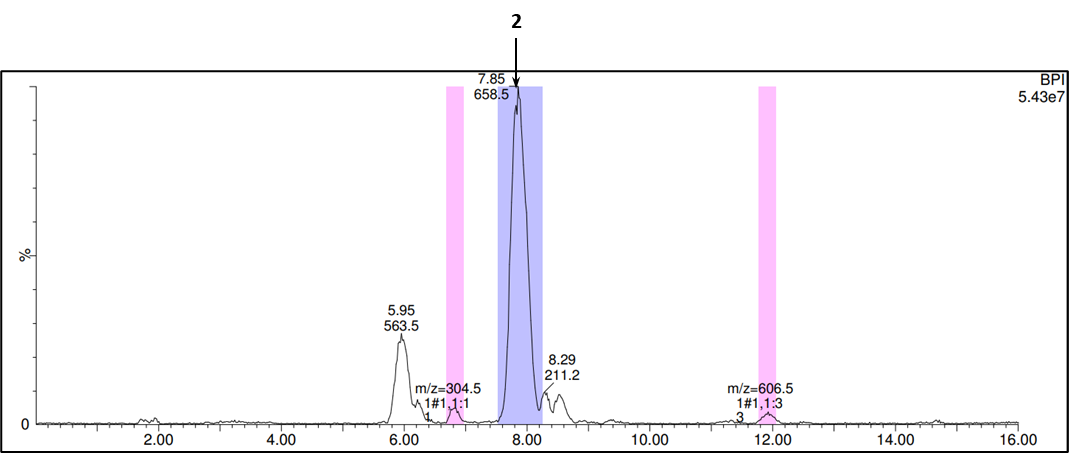


Figure S 7. BPI Chromatogram of the second round of purification of Fr.5B, resulting in a pure compound **2**.


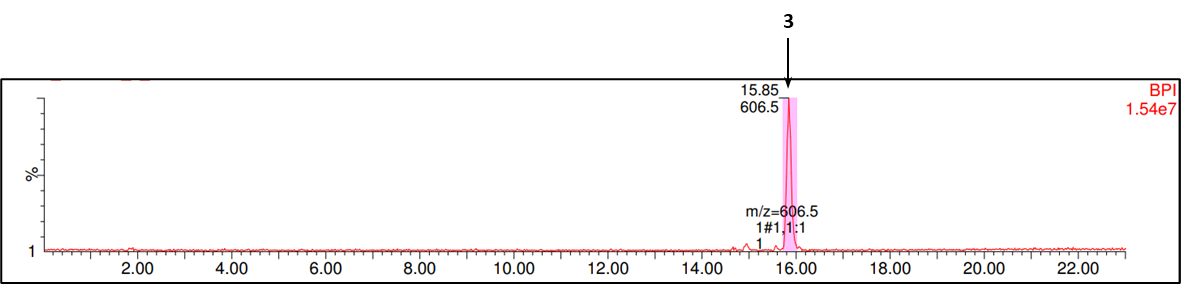


Figure S 8. BPI Chromatogram of Fr.5C. After the first round of purification, it was obtained as a pure compound and named as **3**, without the need for an additional purification step.


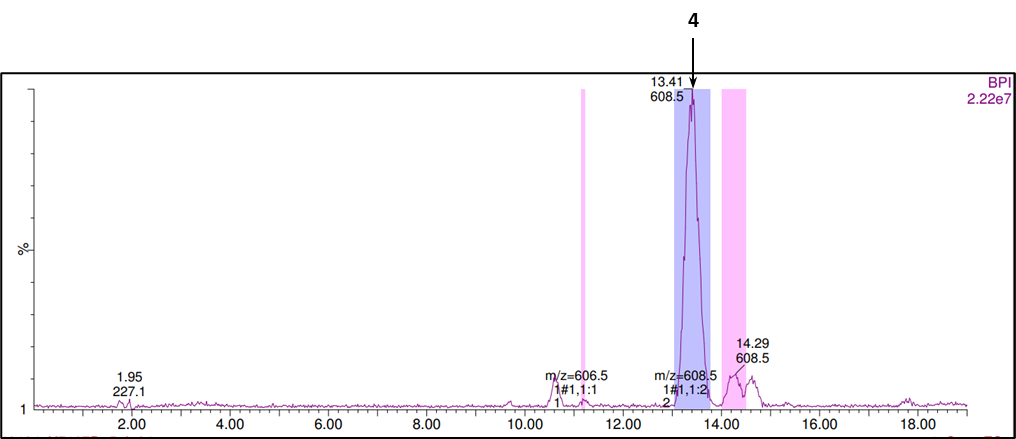


Figure S 9. BPI Chromatogram of the second round of purification of Fr.5D, resulting in a pure compound **4**.


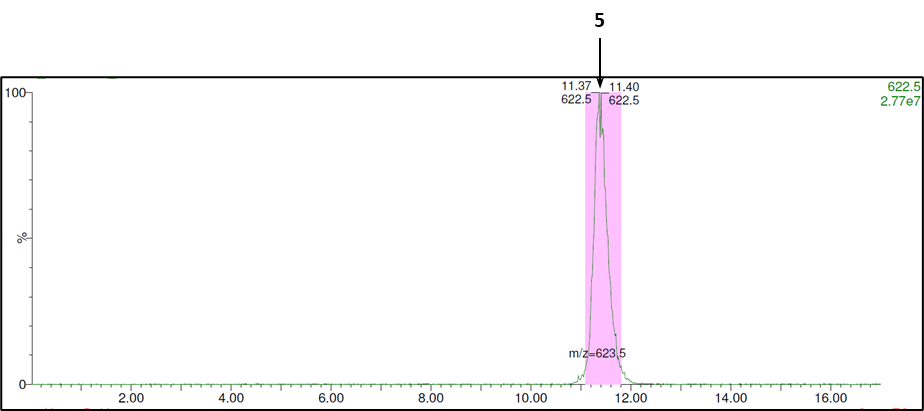


Figure S 10. BPI Chromatogram of the second round of purification of Fr.5E, resulting in a pure compound **5**.

**Compound 1**


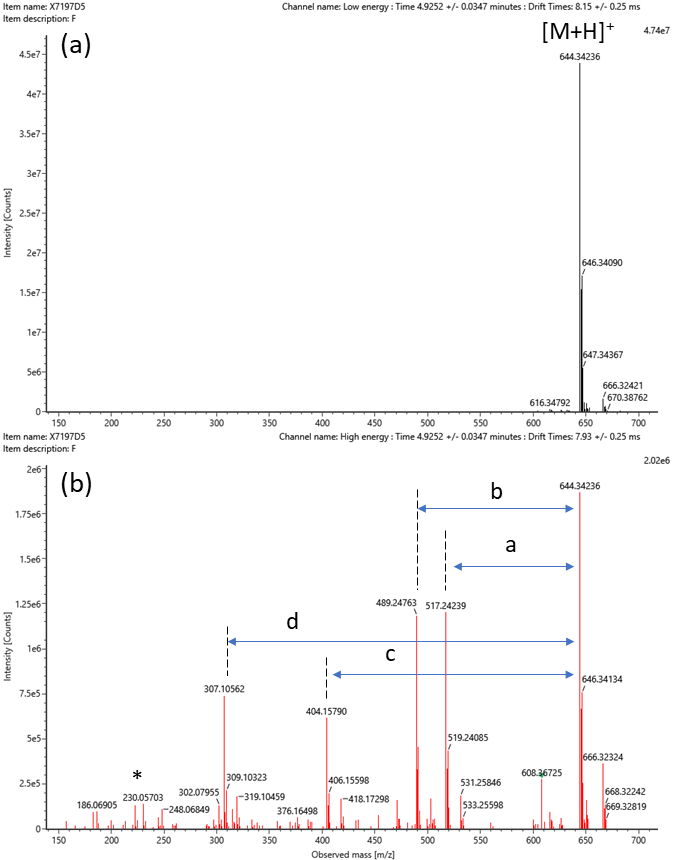


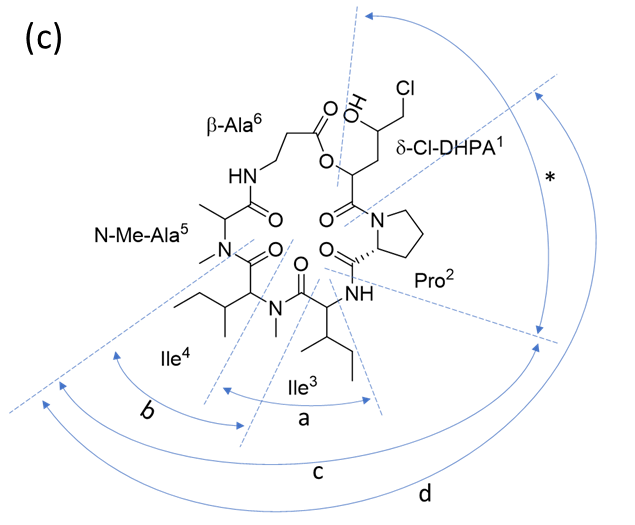


Figure S 11. HRESIMS of **1** at (a) low collision energy and (b) high collision energy in ESI+, HDMS^E^ mode. (c) Fragmentation of **1**.


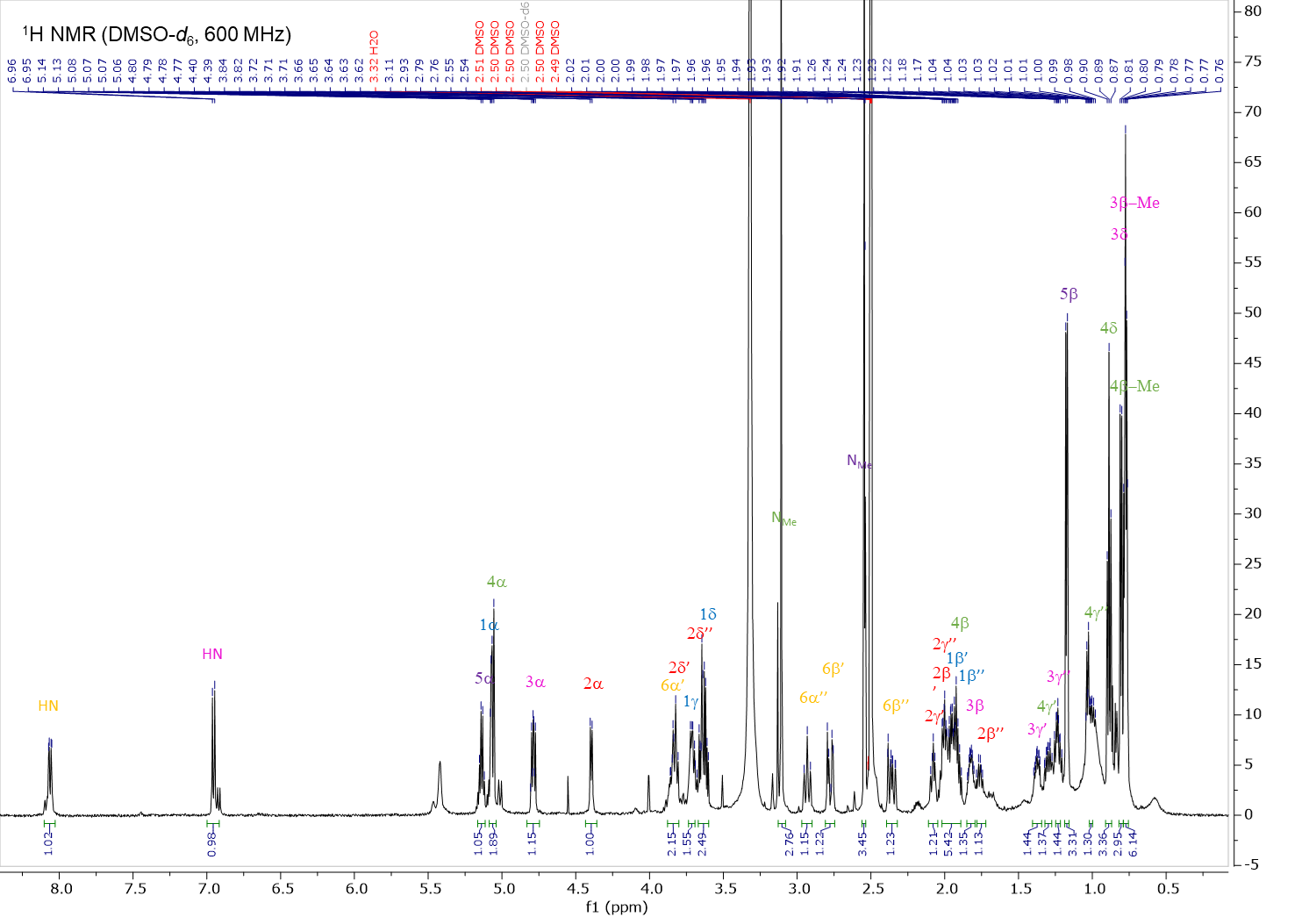


Figure S 12. ^1^H NMR spectrum of **1**.


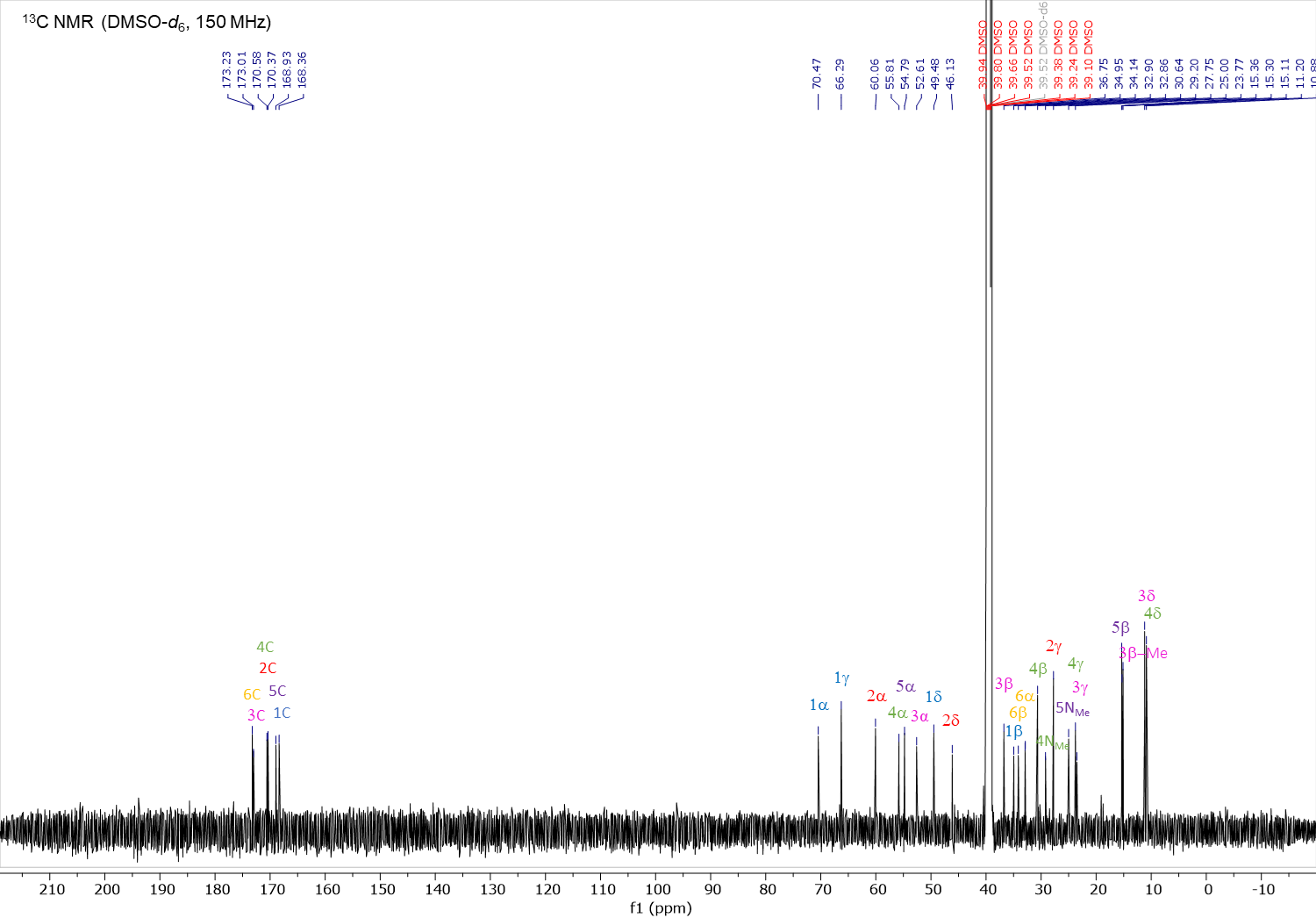


Figure S 13. ^13^C NMR spectrum of **1**.


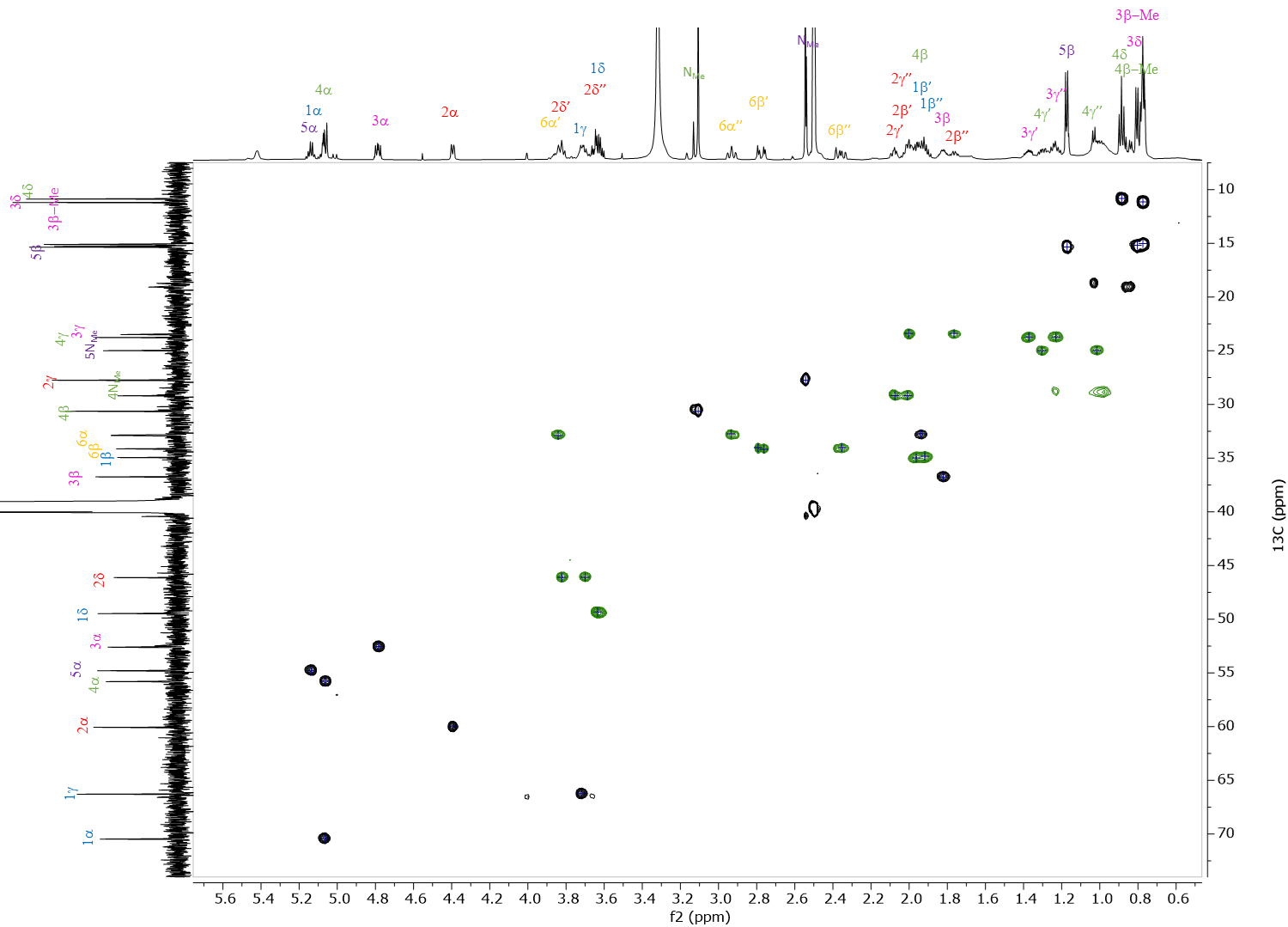


Figure S 14. HSQC spectrum of **1**.


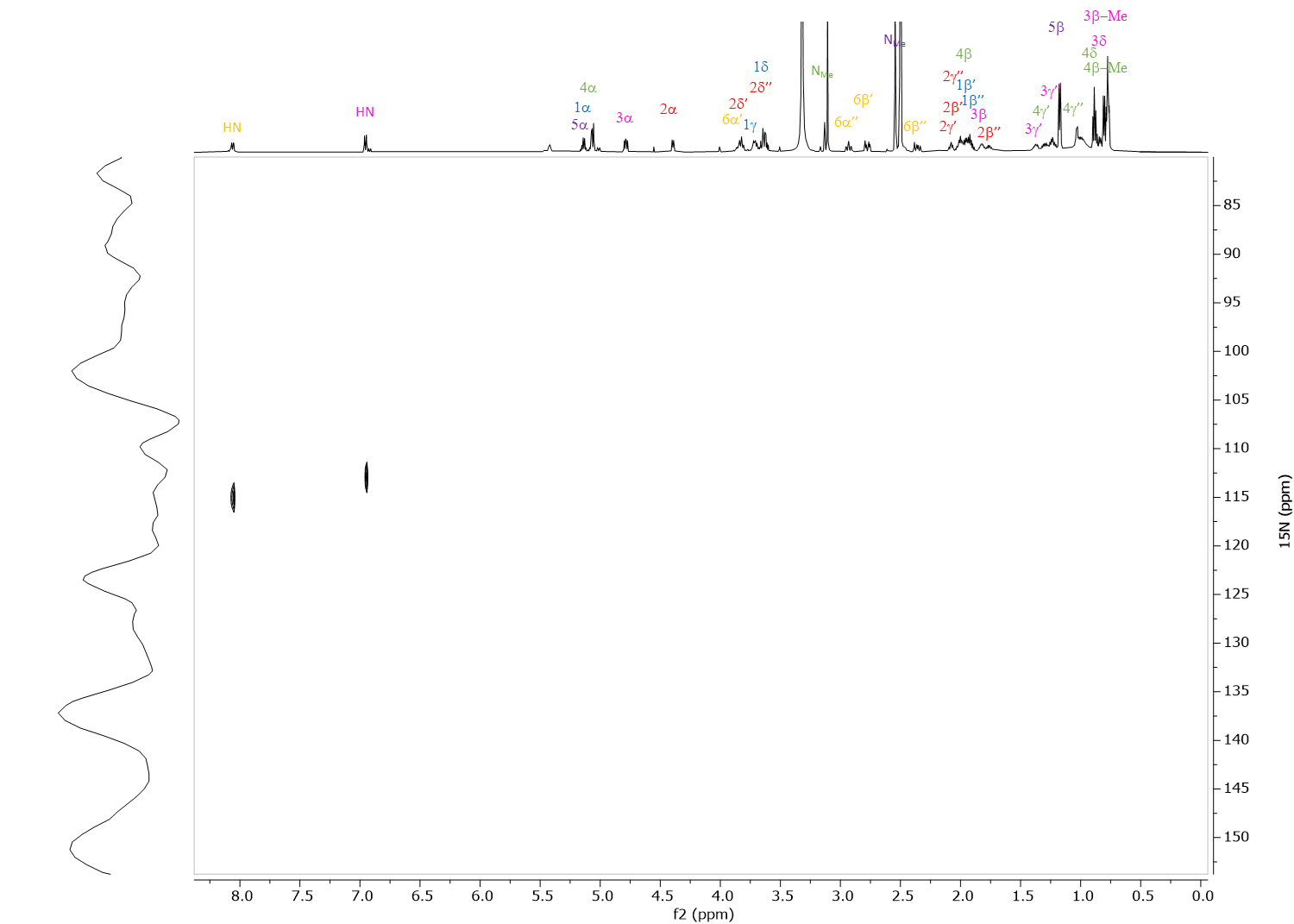


Figure S 15. ^1^H, ^15^N-HSQC spectrum of **1**.


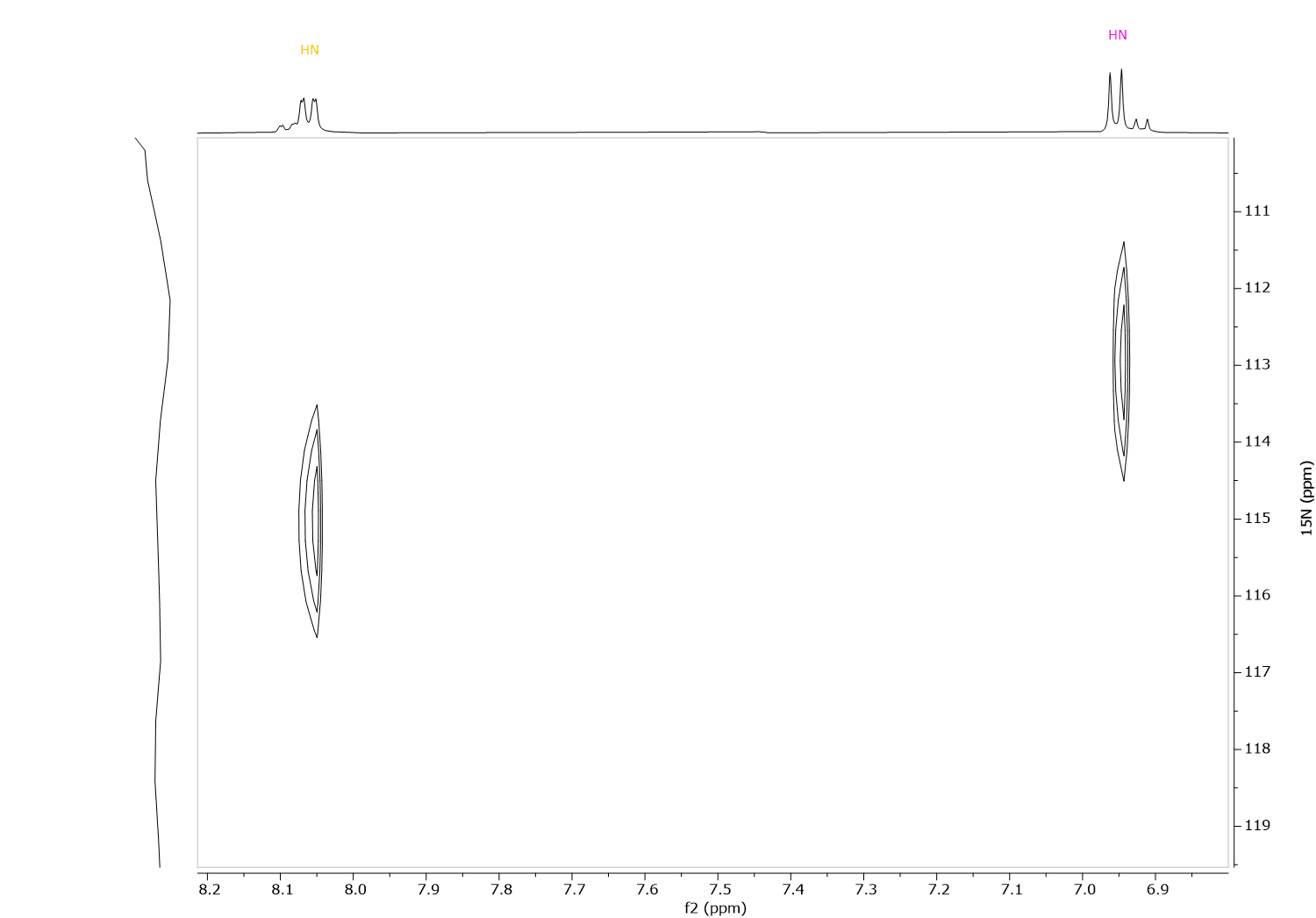


Figure S 16. ^1^H,^15^N-HSQC (Expanded) spectrum of **1**.


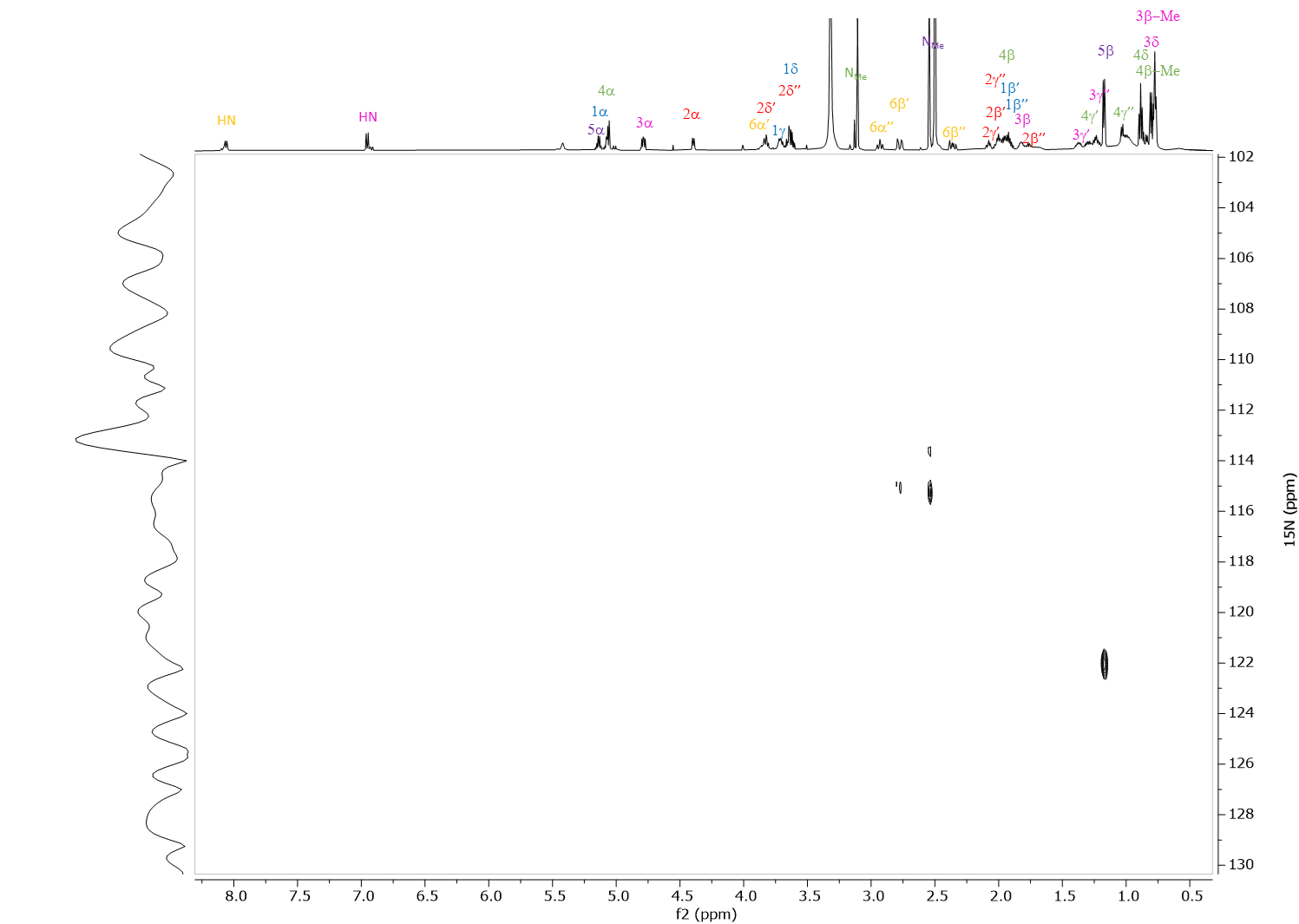


Figure S 17. ^1^H, ^15^N-HMBC spectrum of **1**.


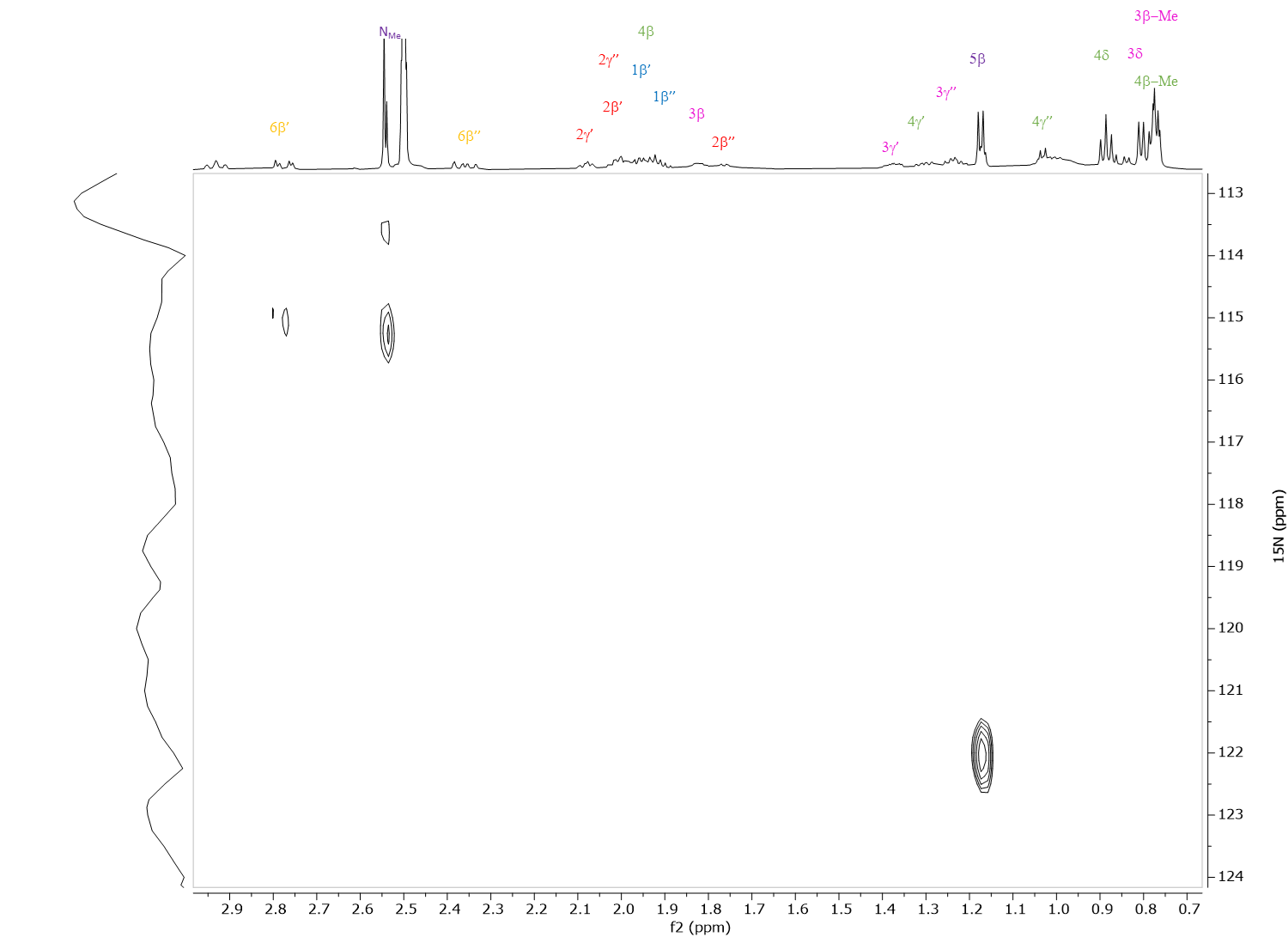


Figure S 18. ^1^H,^15^N-HMBC (Expanded) spectrum of **1**.


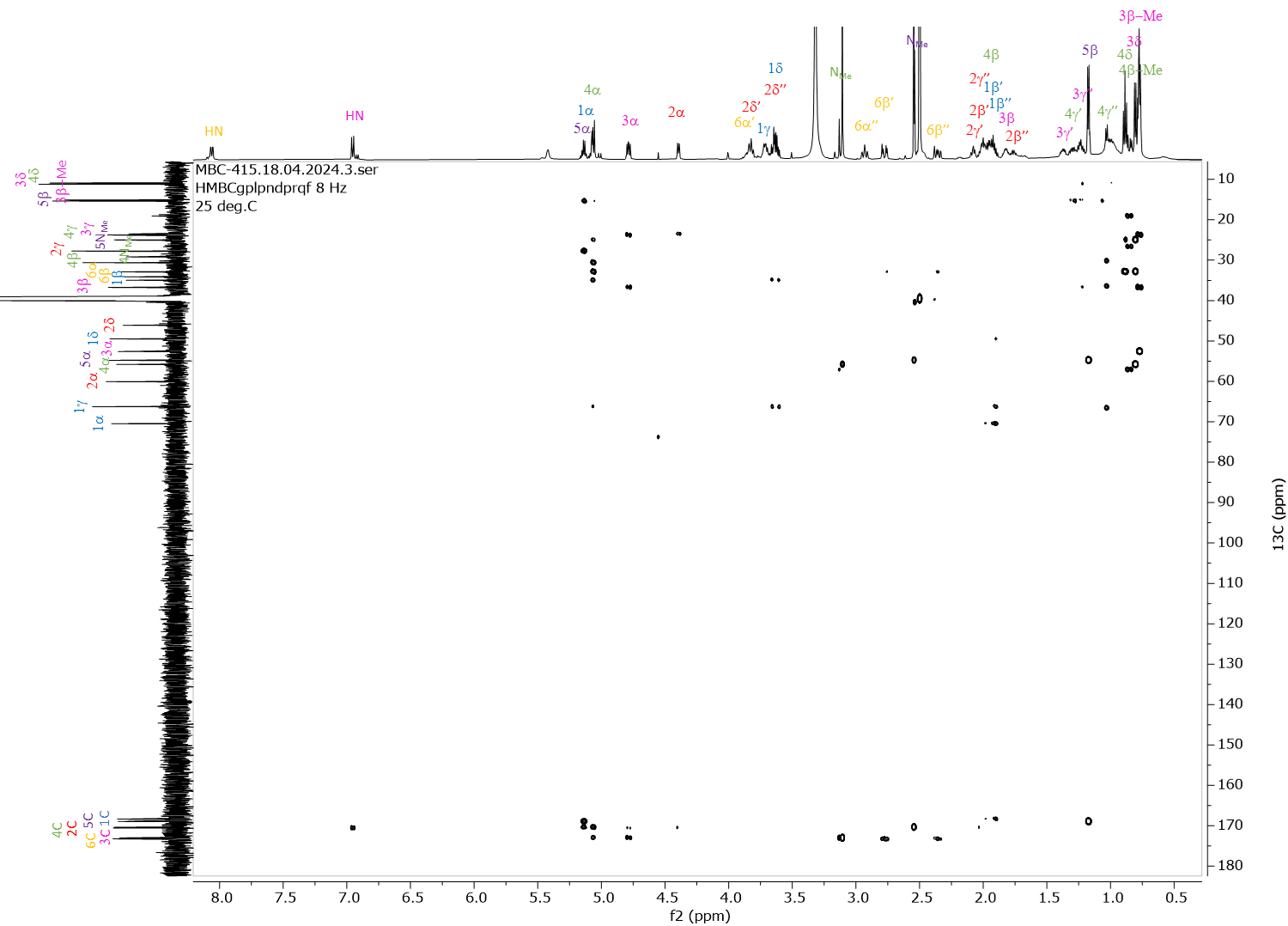


Figure S 19. HMBC spectrum of **1**.


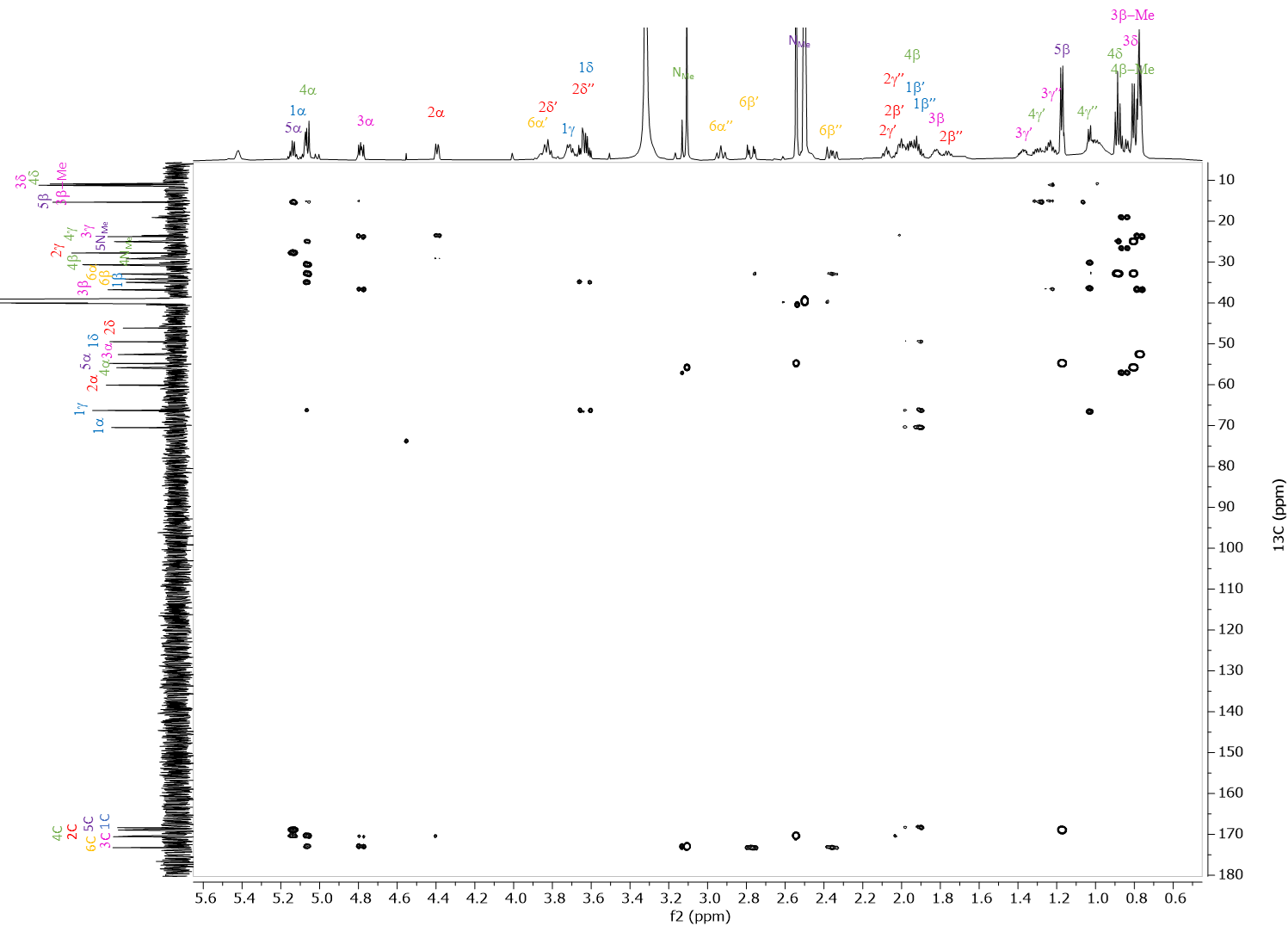


Figure S 20. HMBC (8Hz long range) spectrum of **1**.


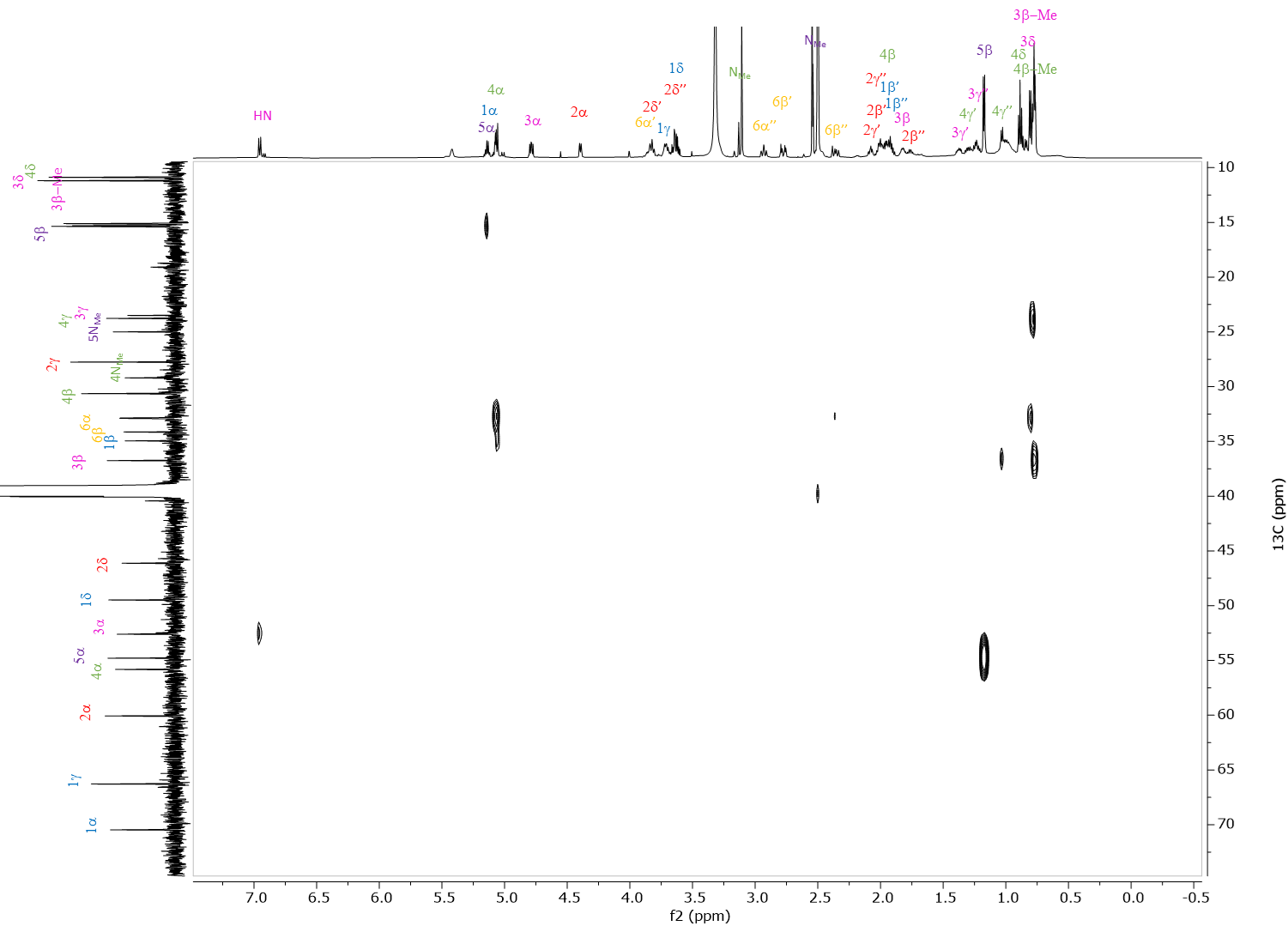


Figure S 21. H2BC spectrum of **1**.


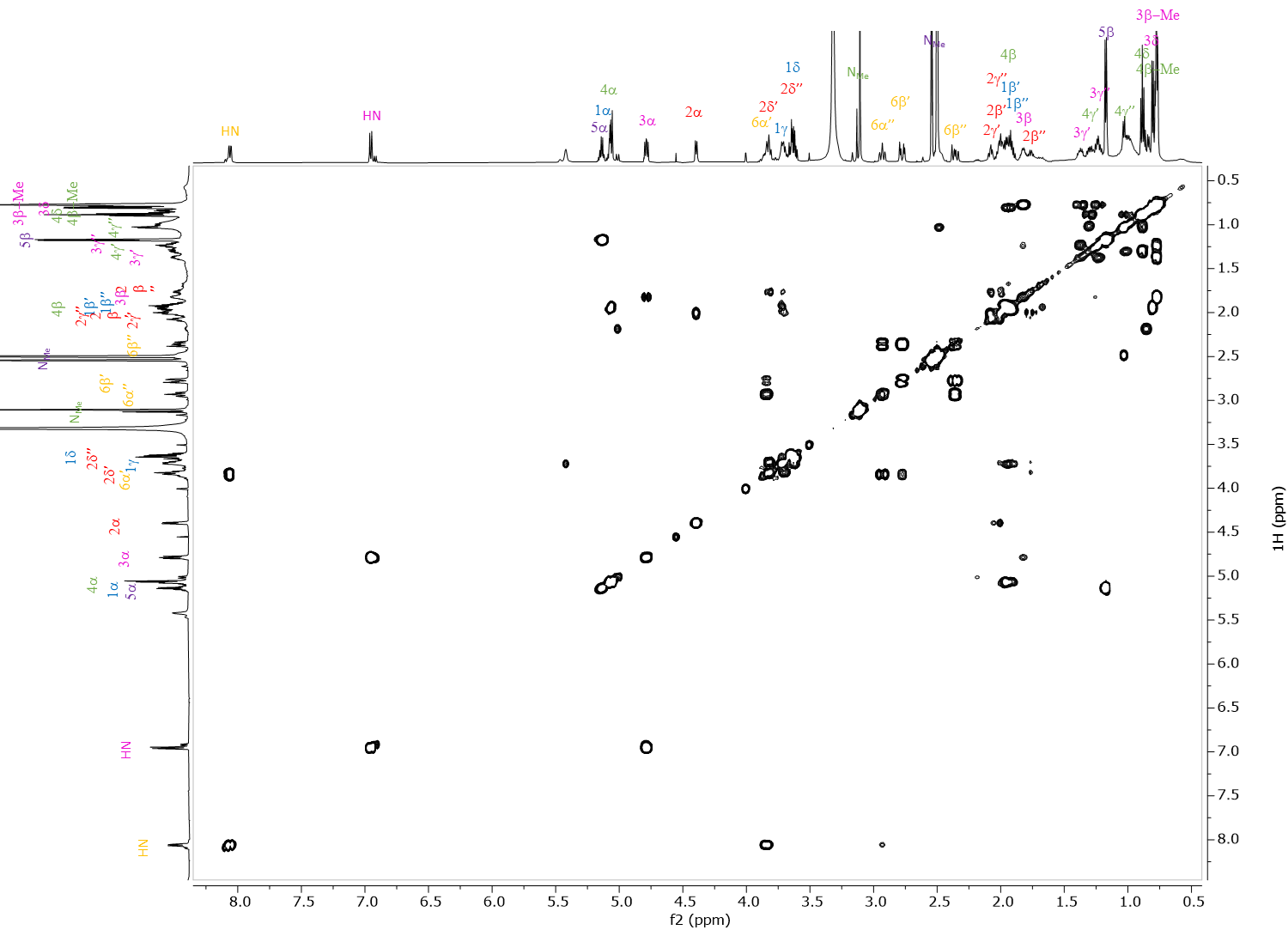


Figure S 22. DQF-COSY spectrum of **1**.


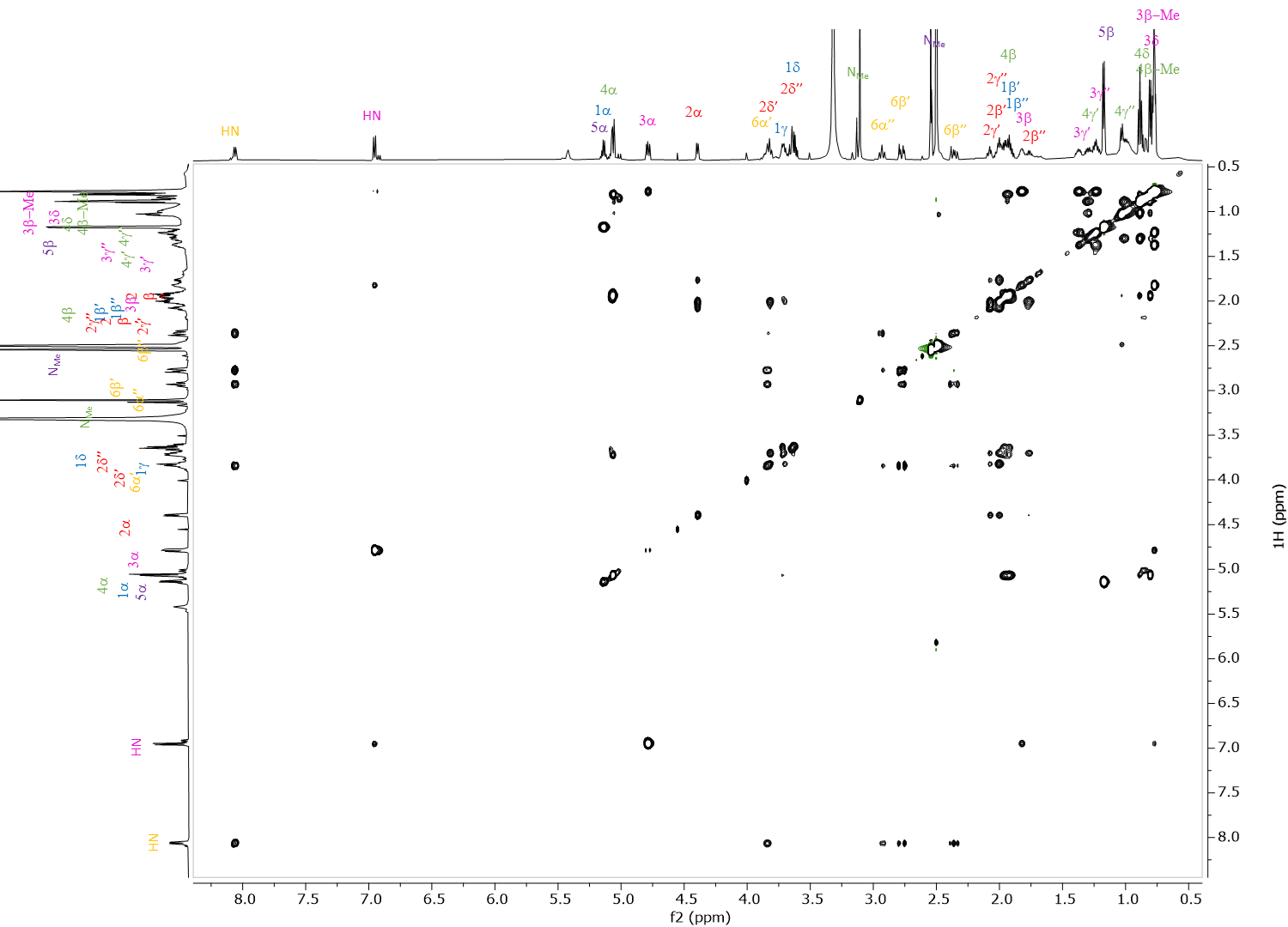


Figure S 23. TOCSY ((DIPSI2) 60ms) spectrum of **1**.


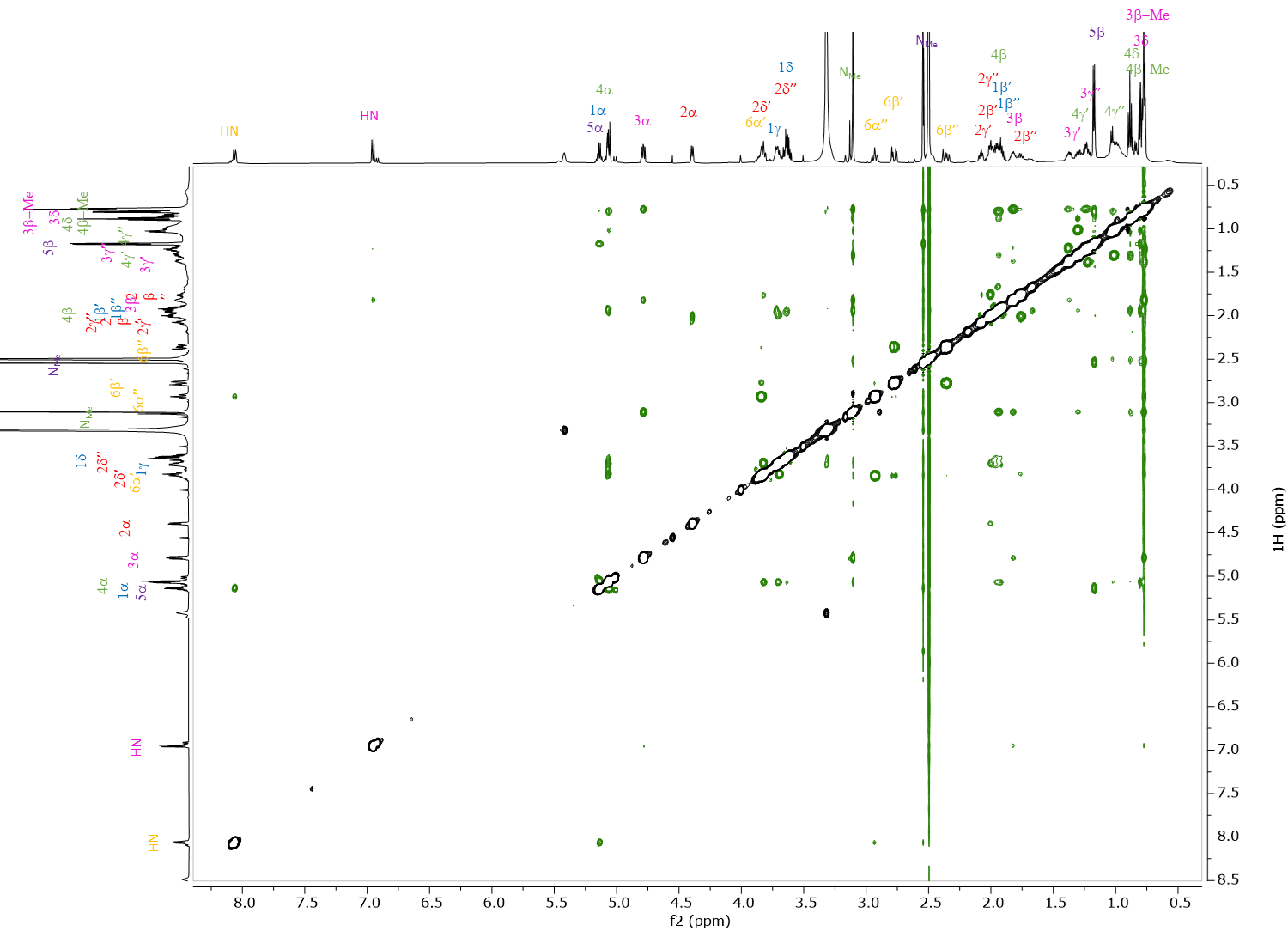


Figure S 24. ROESY (300 ms) spectrum of **1**.

**Compound 2**


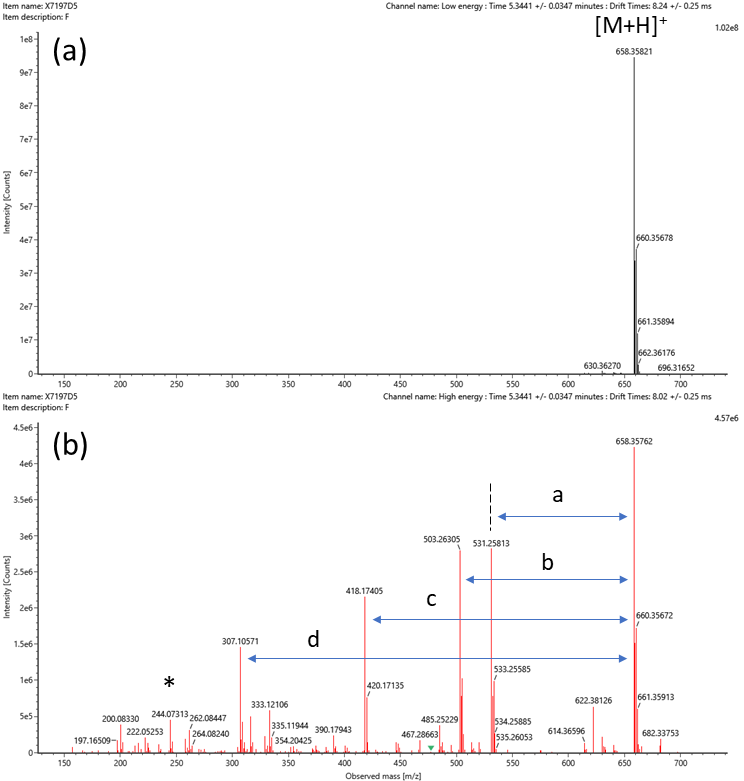


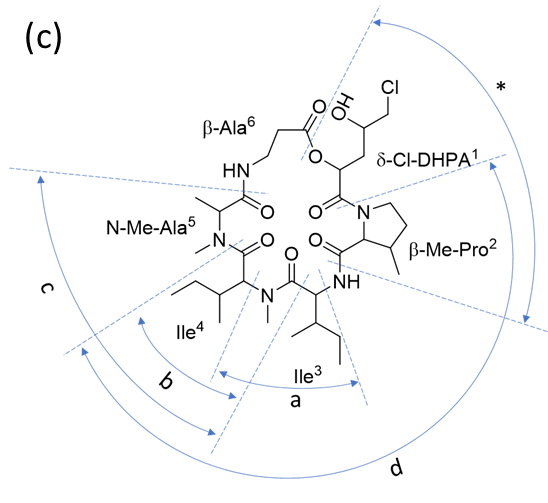


Figure S 25. HRESIMS of **2** at (a) low collision energy and (b) high collision energy in ESI+, HDMS^E^ mode. (c) Fragmentation of **2**.


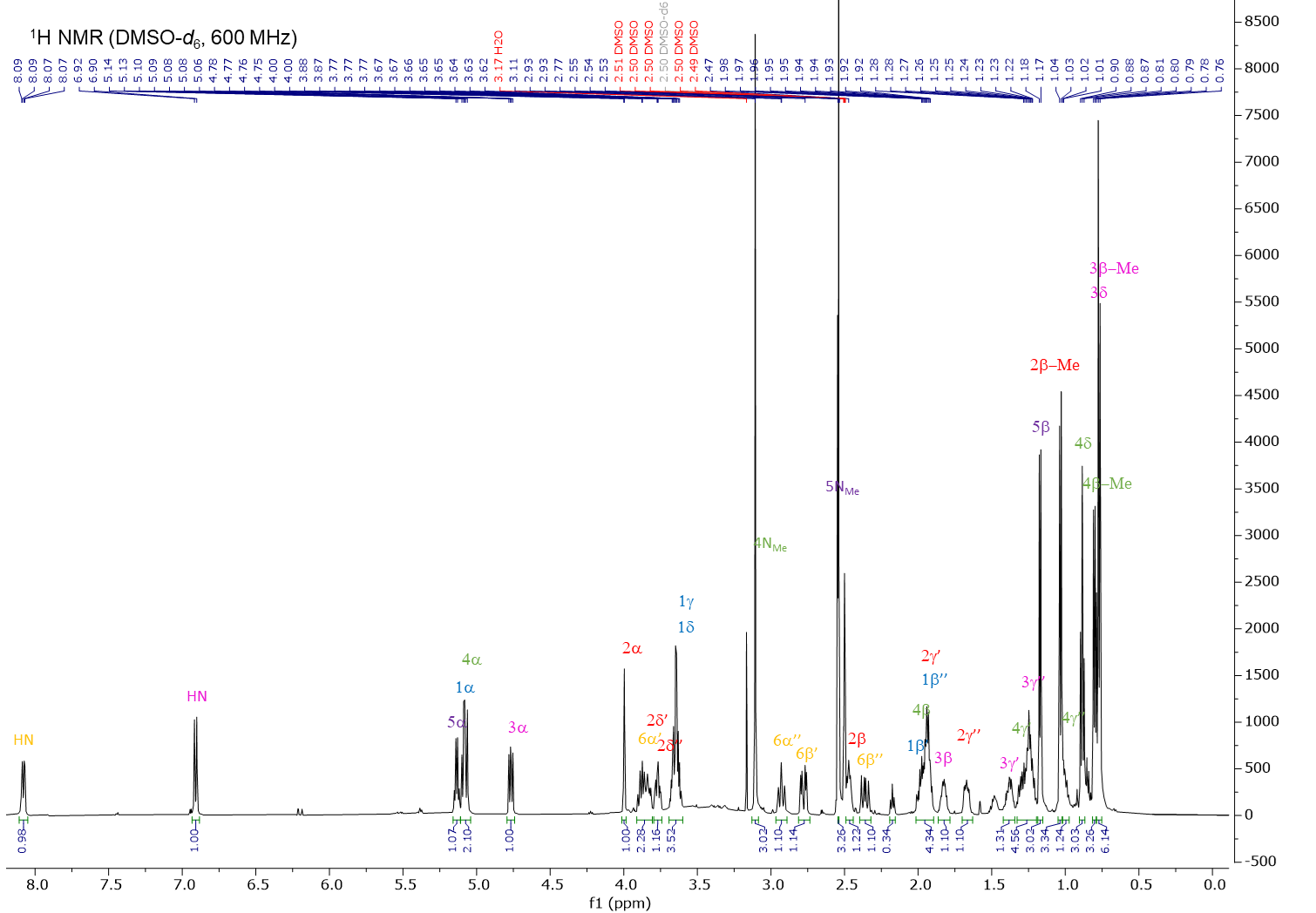


Figure S 26. ^1^H NMR spectrum of **2**.


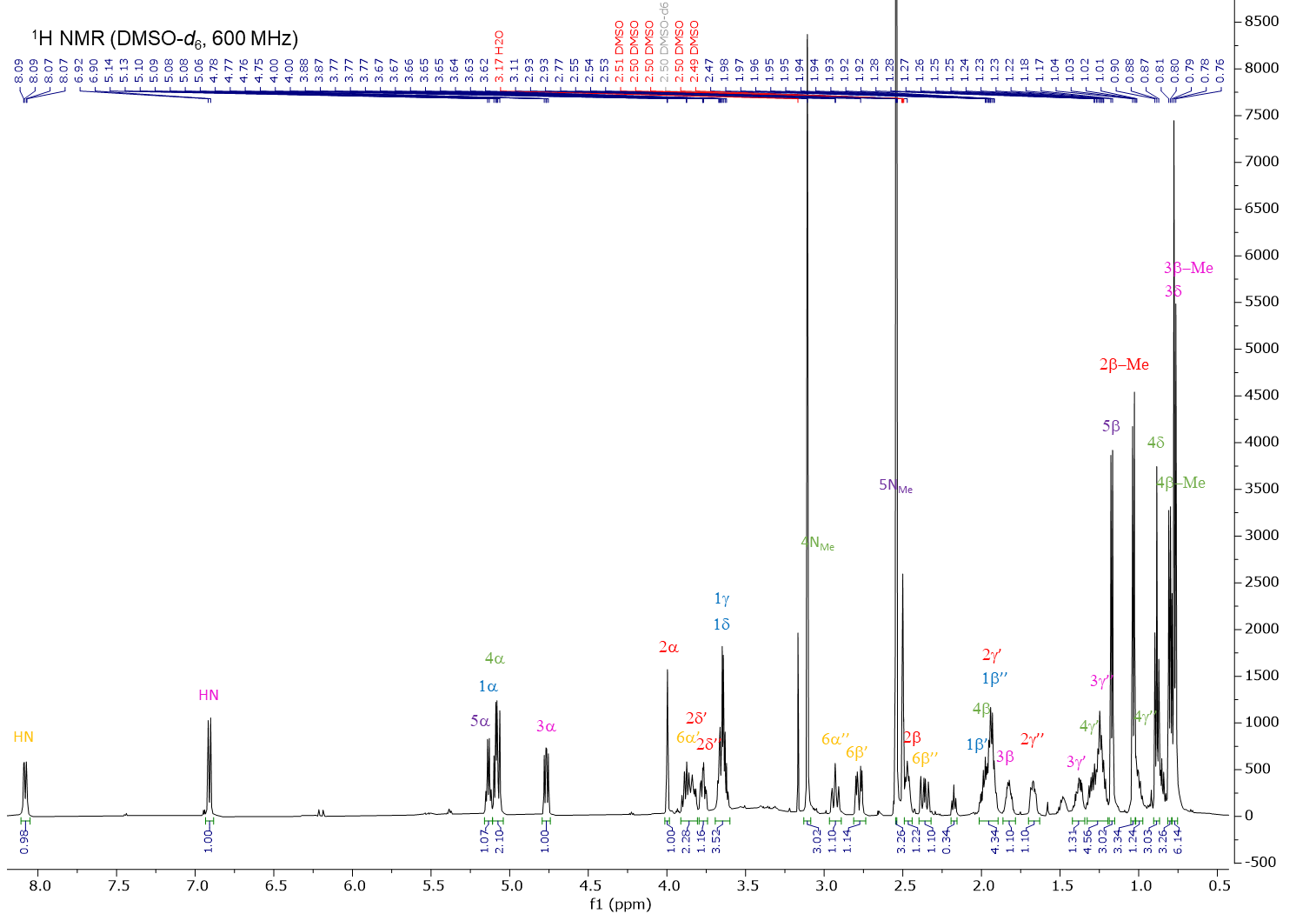


Figure S 27. ^1^H NMR (Expanded) spectrum of **2**.


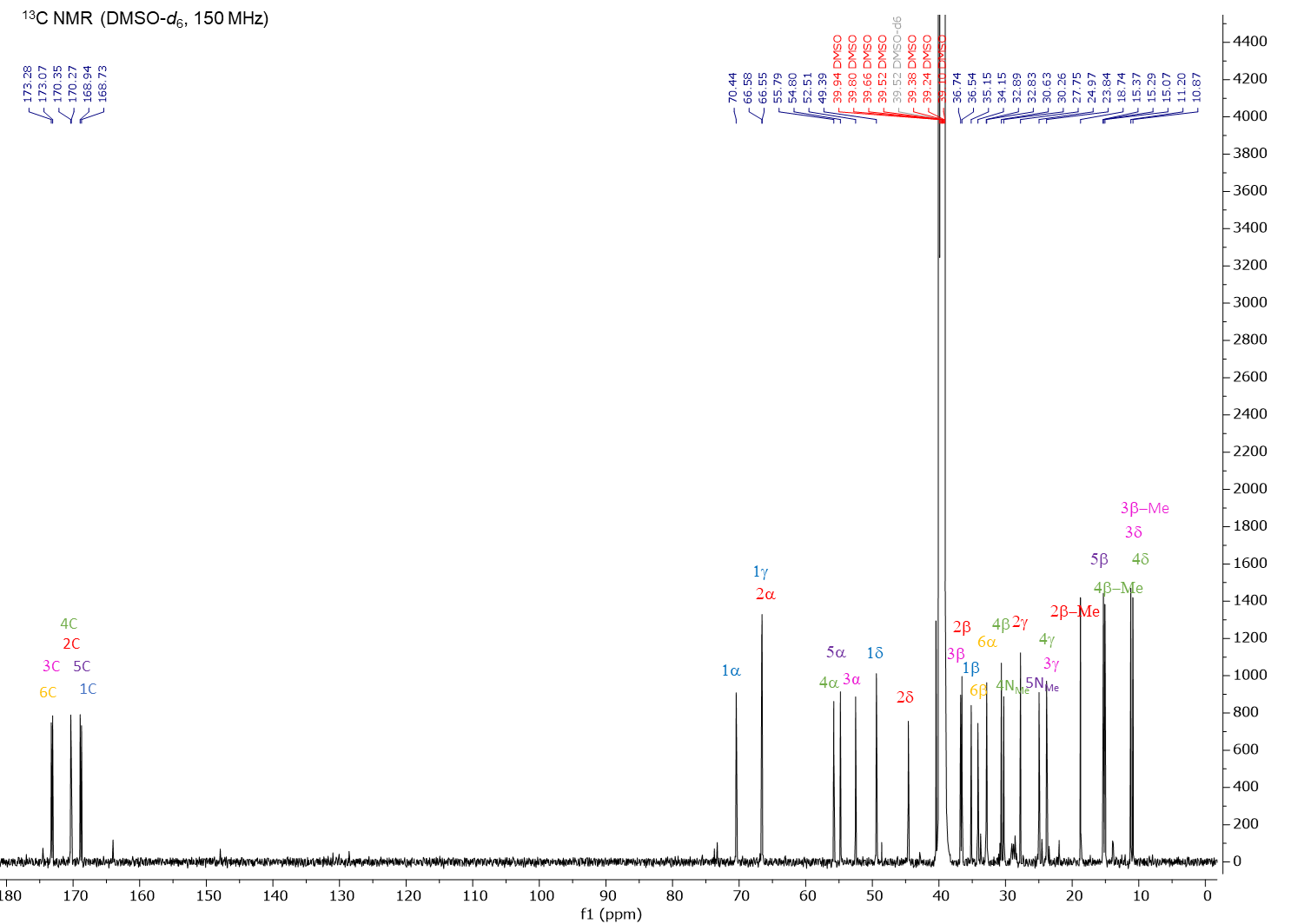


Figure S 28. ^13^C NMR spectrum of **2**.


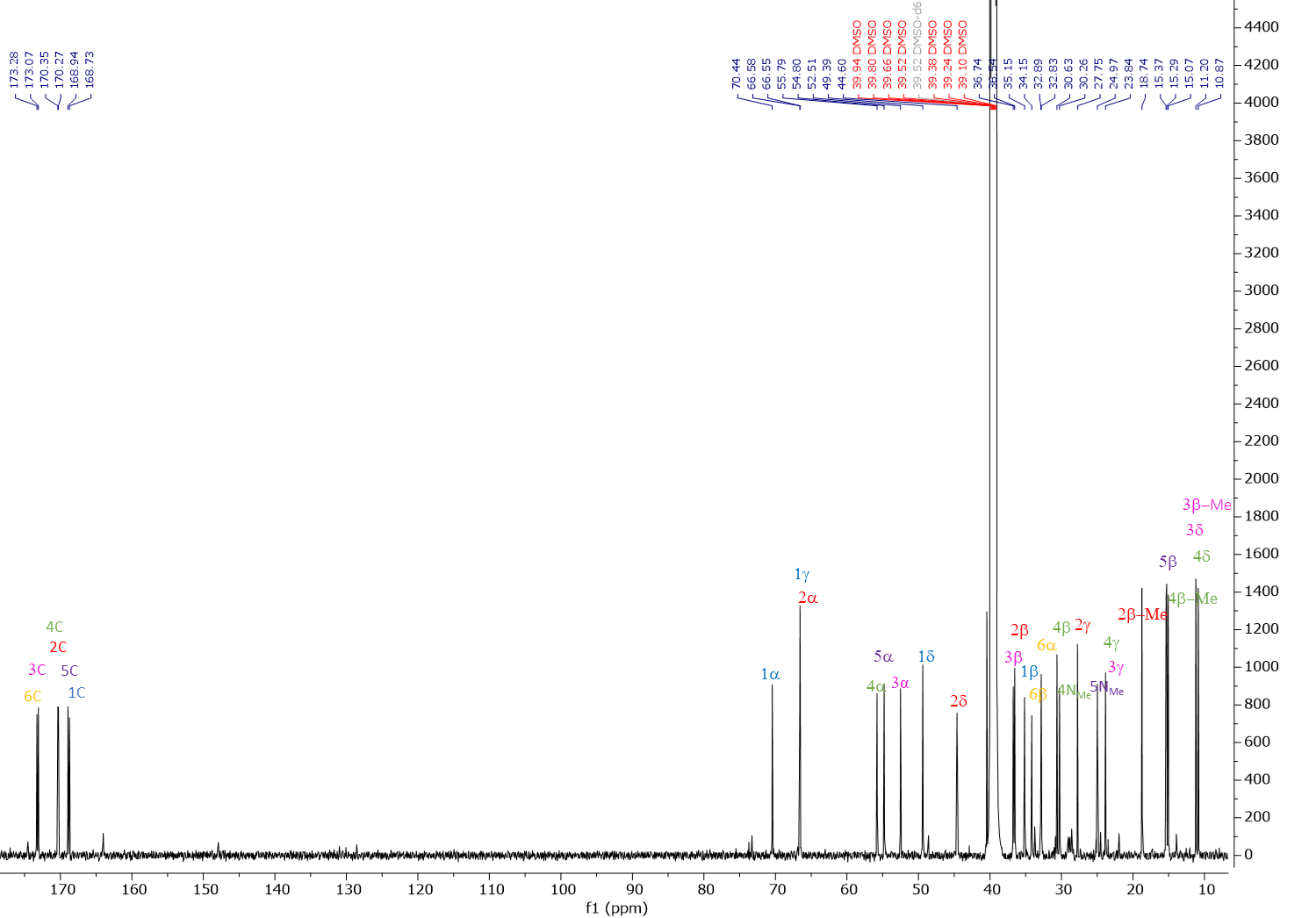


Figure S 29. ^13^C NMR (Expanded) spectrum of **2**.


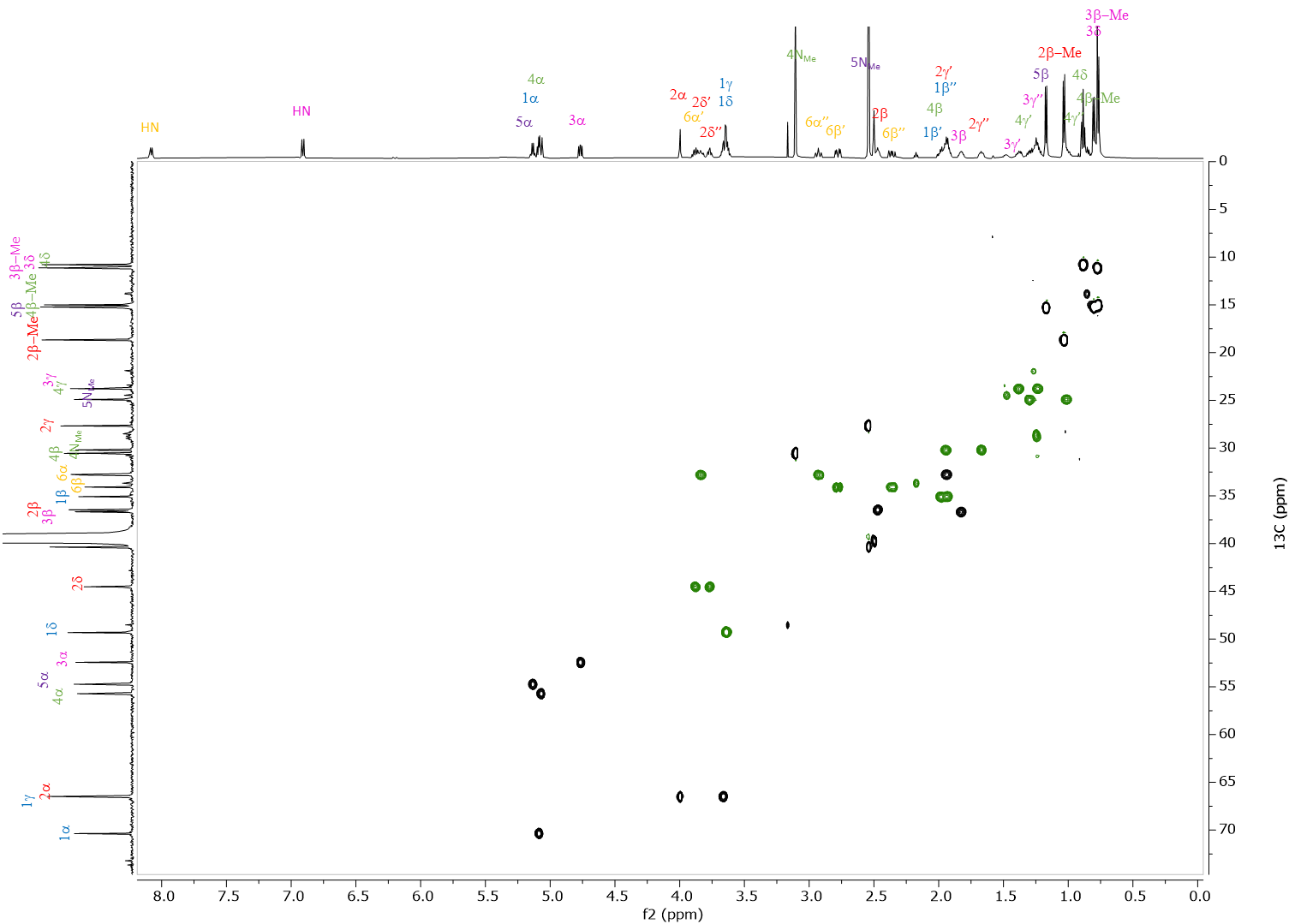


Figure S 30. HSQC spectrum of **2**.


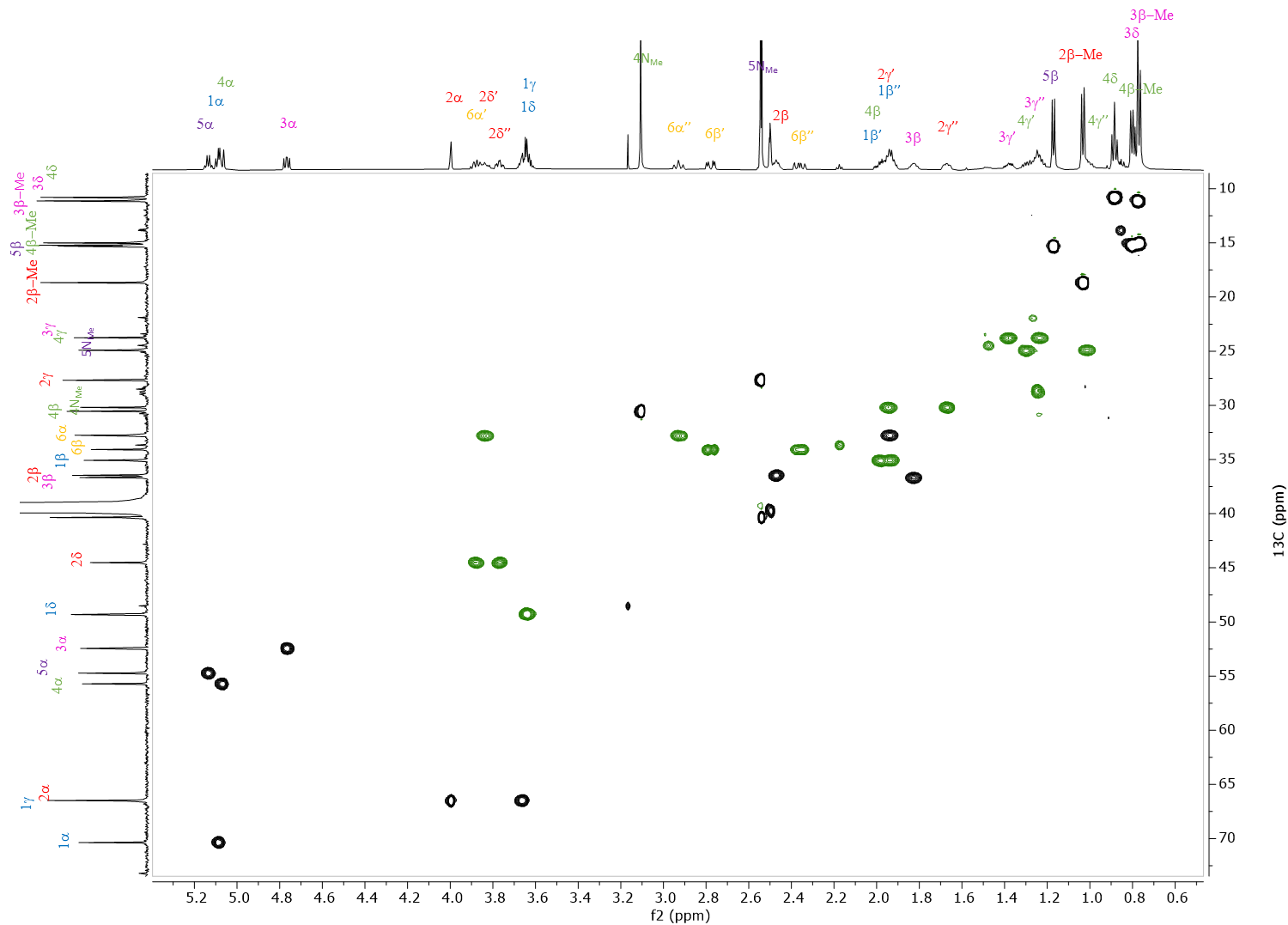


Figure S 31. HSQC (Expanded) spectrum of **2**.


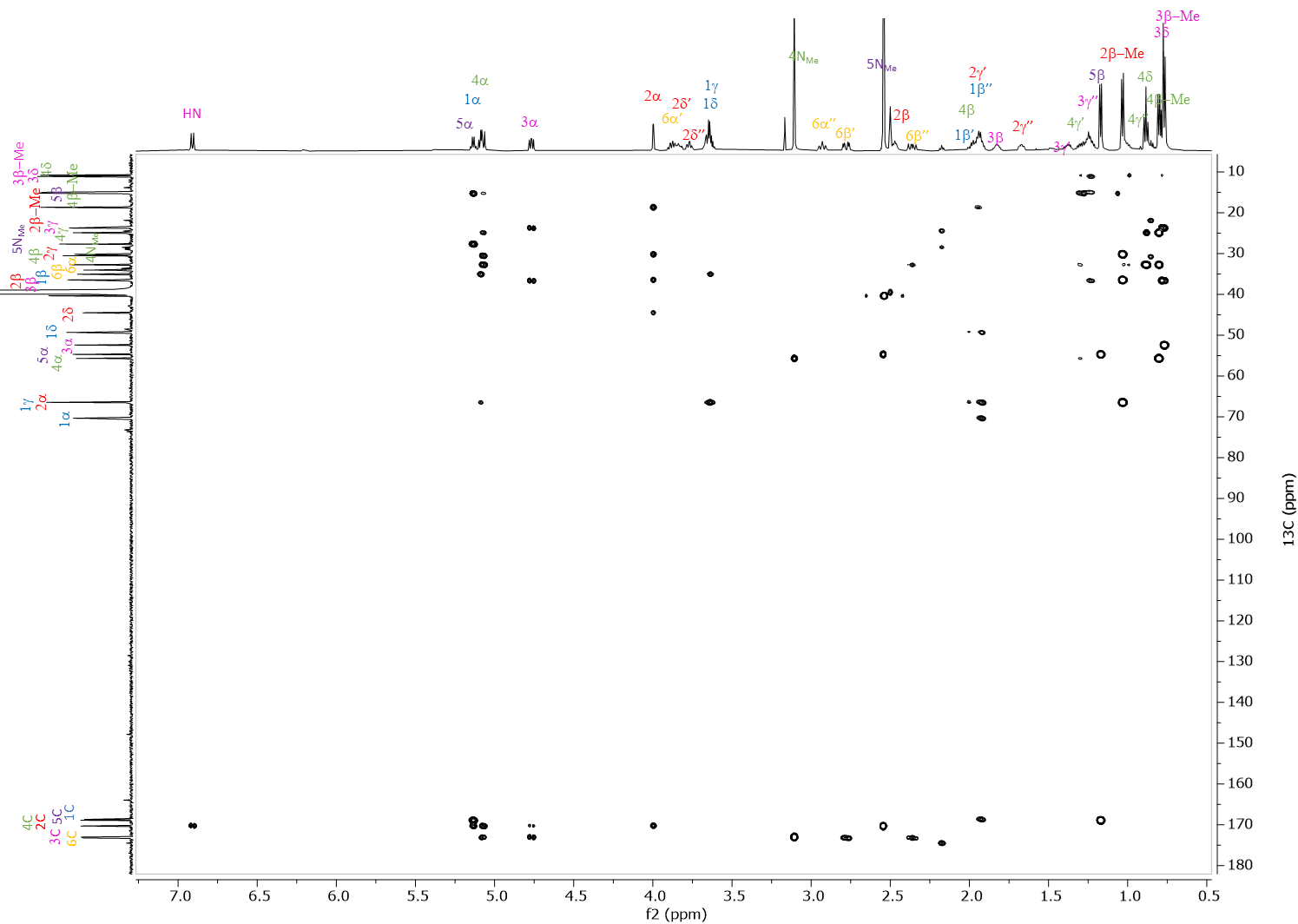


Figure S 32. HMBC spectrum of **2**.


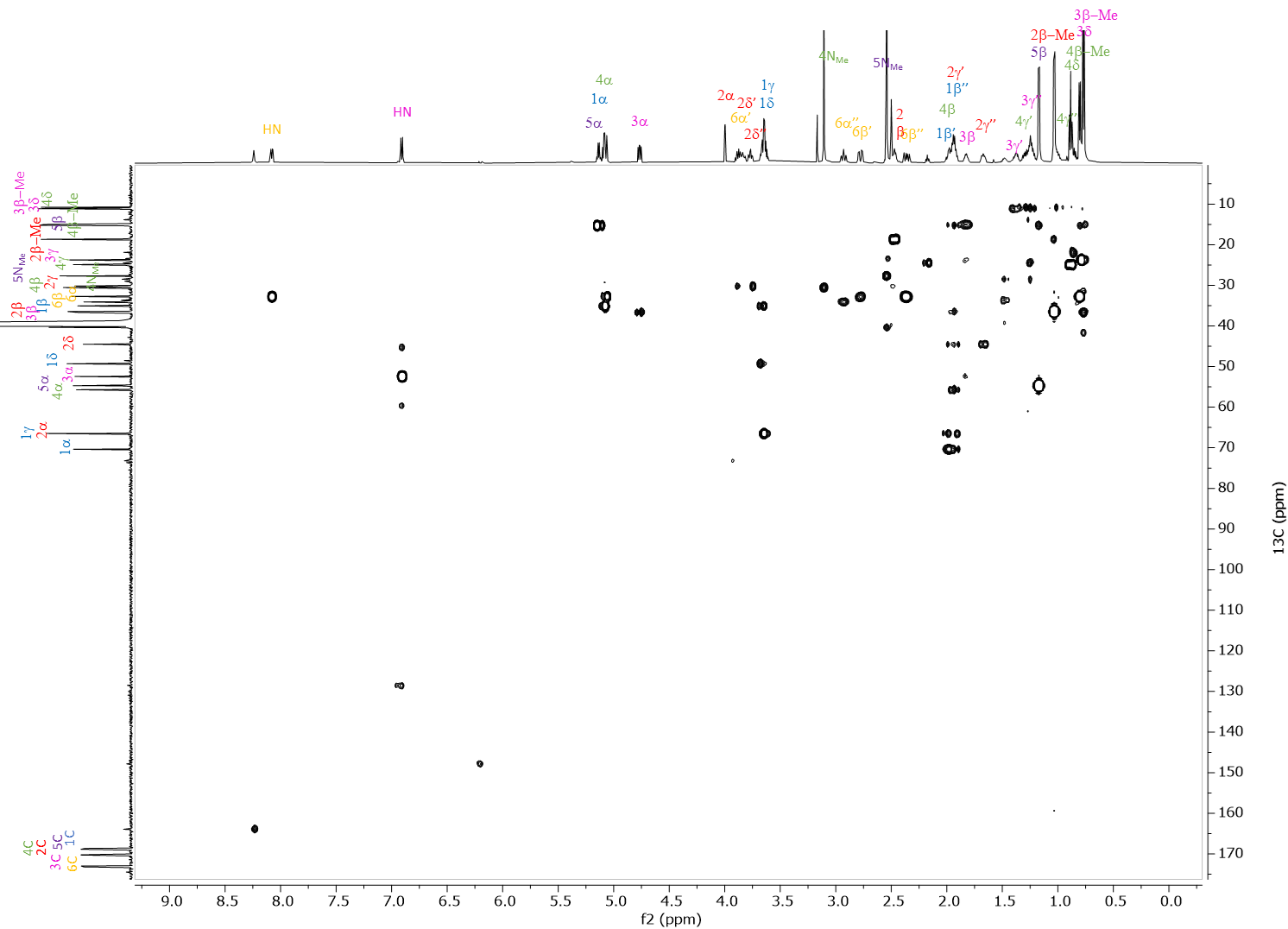


Figure S 33. H2BC spectrum of **2**.


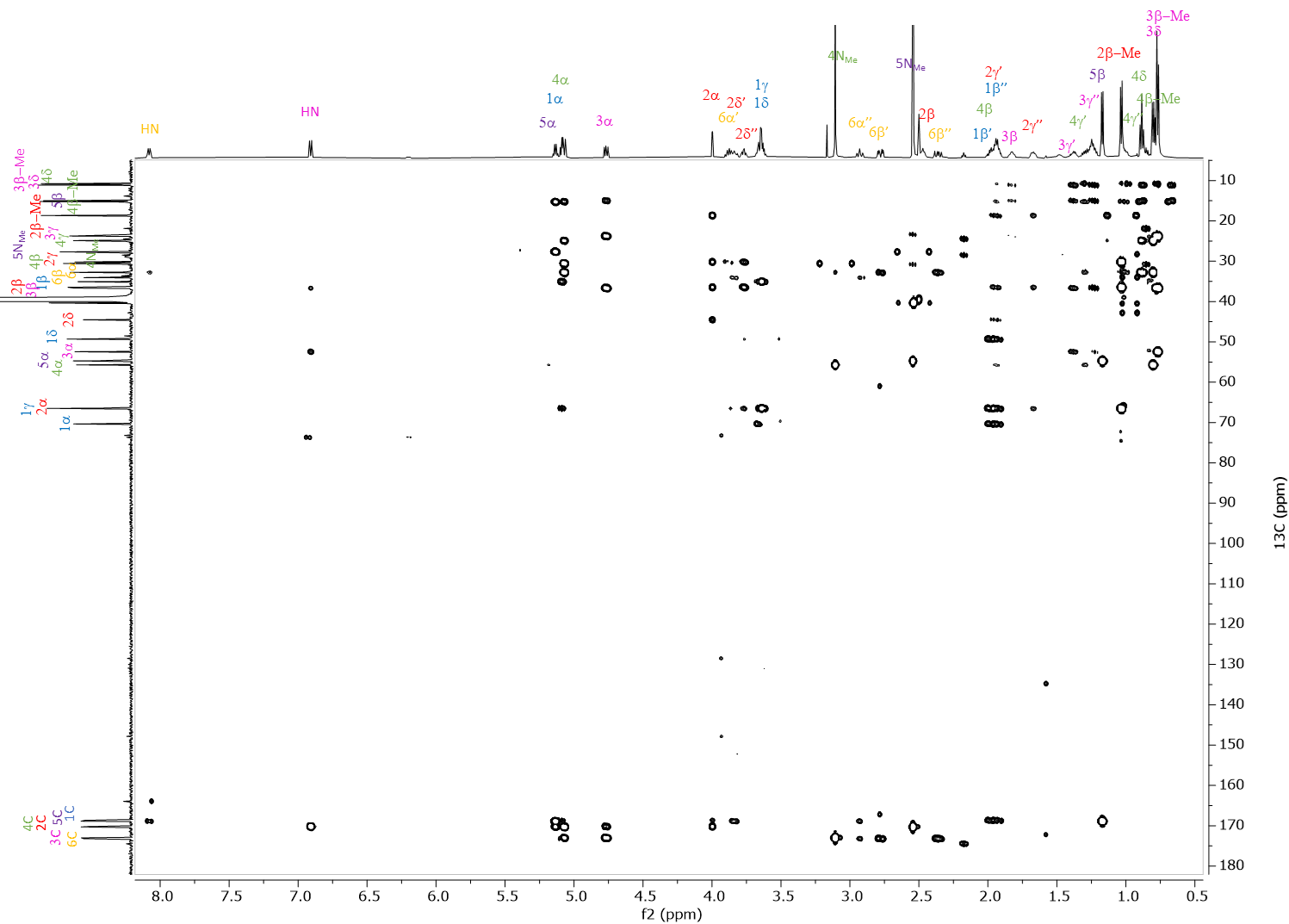


Figure S 34. H2BC (5 Hz long range) spectrum of **2**.


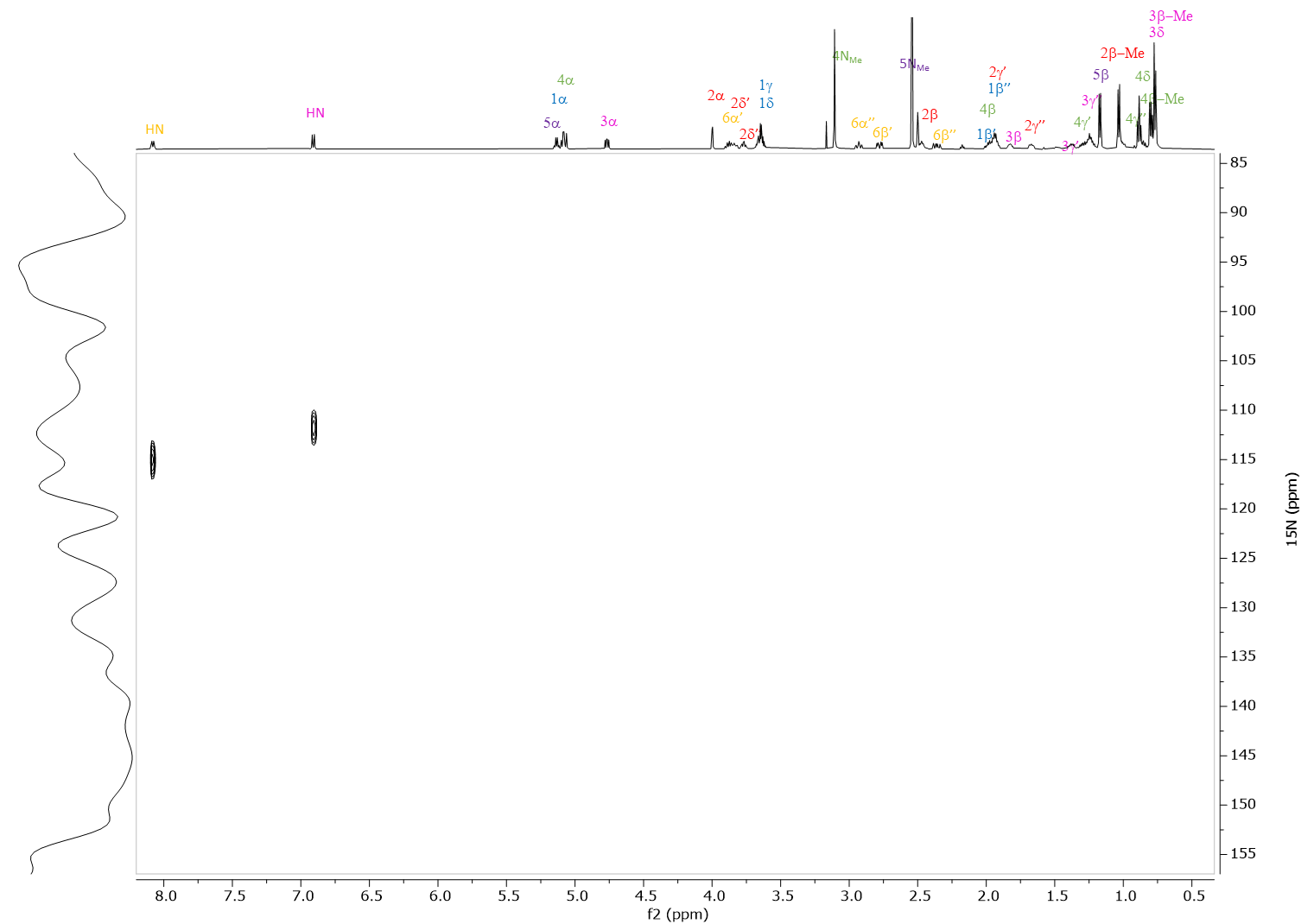


Figure S 35. ^1^H,^15^N-HSQC spectrum of **2**.


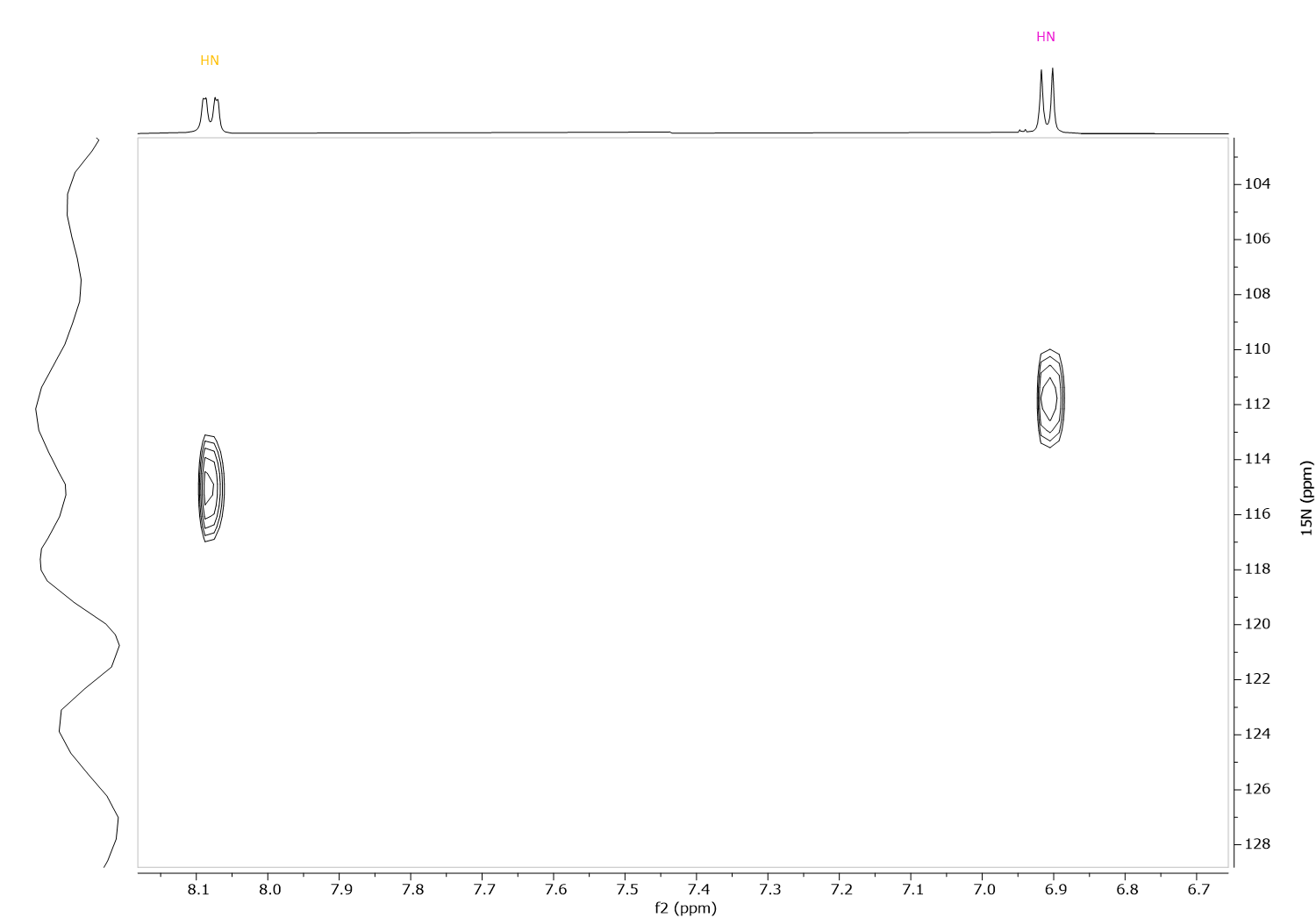


Figure S 36. ^1^H,^15^N-HSQC (Expanded) spectrum of **2**.


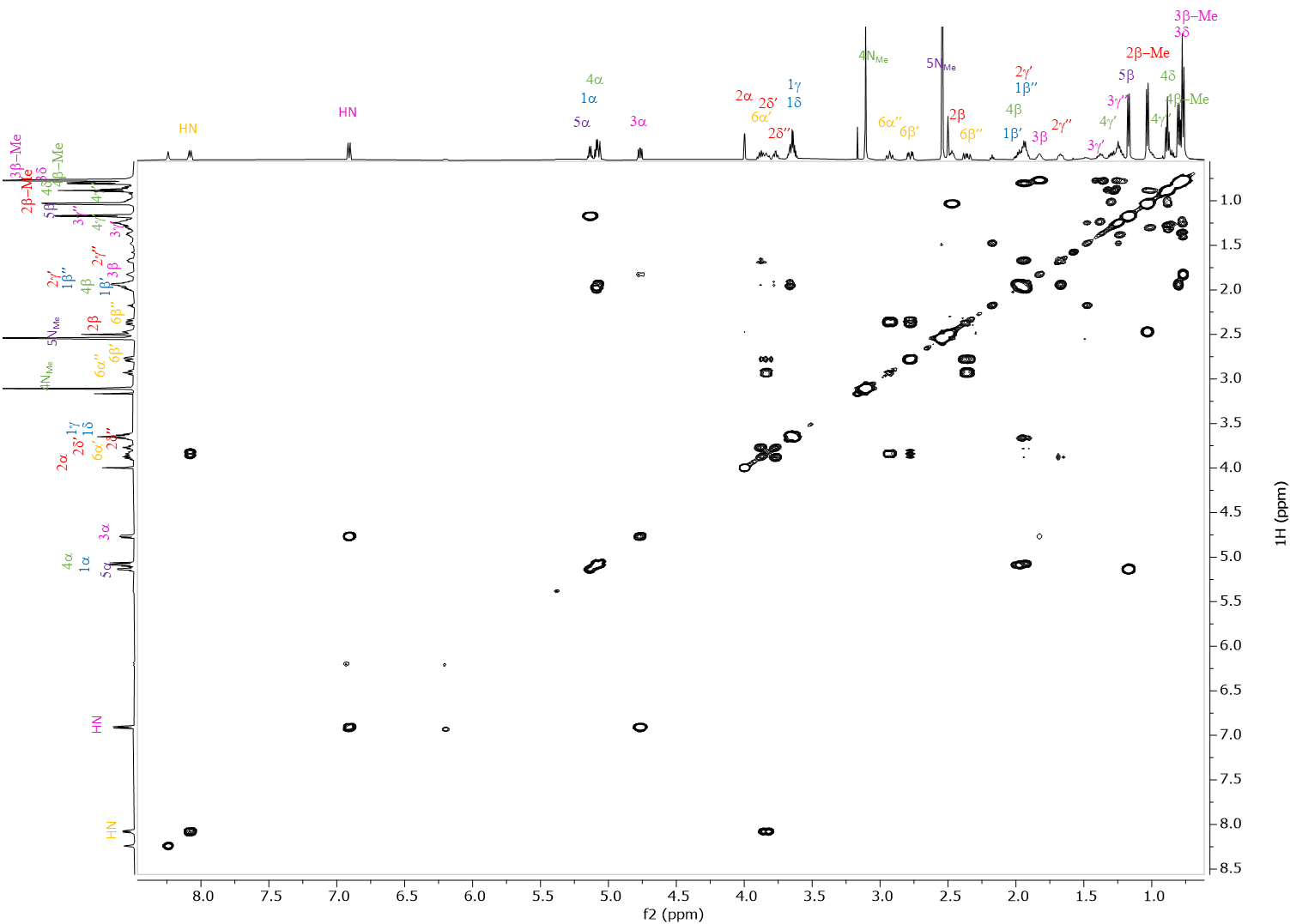


Figure S 37. DQF-COSY spectrum of **2**.


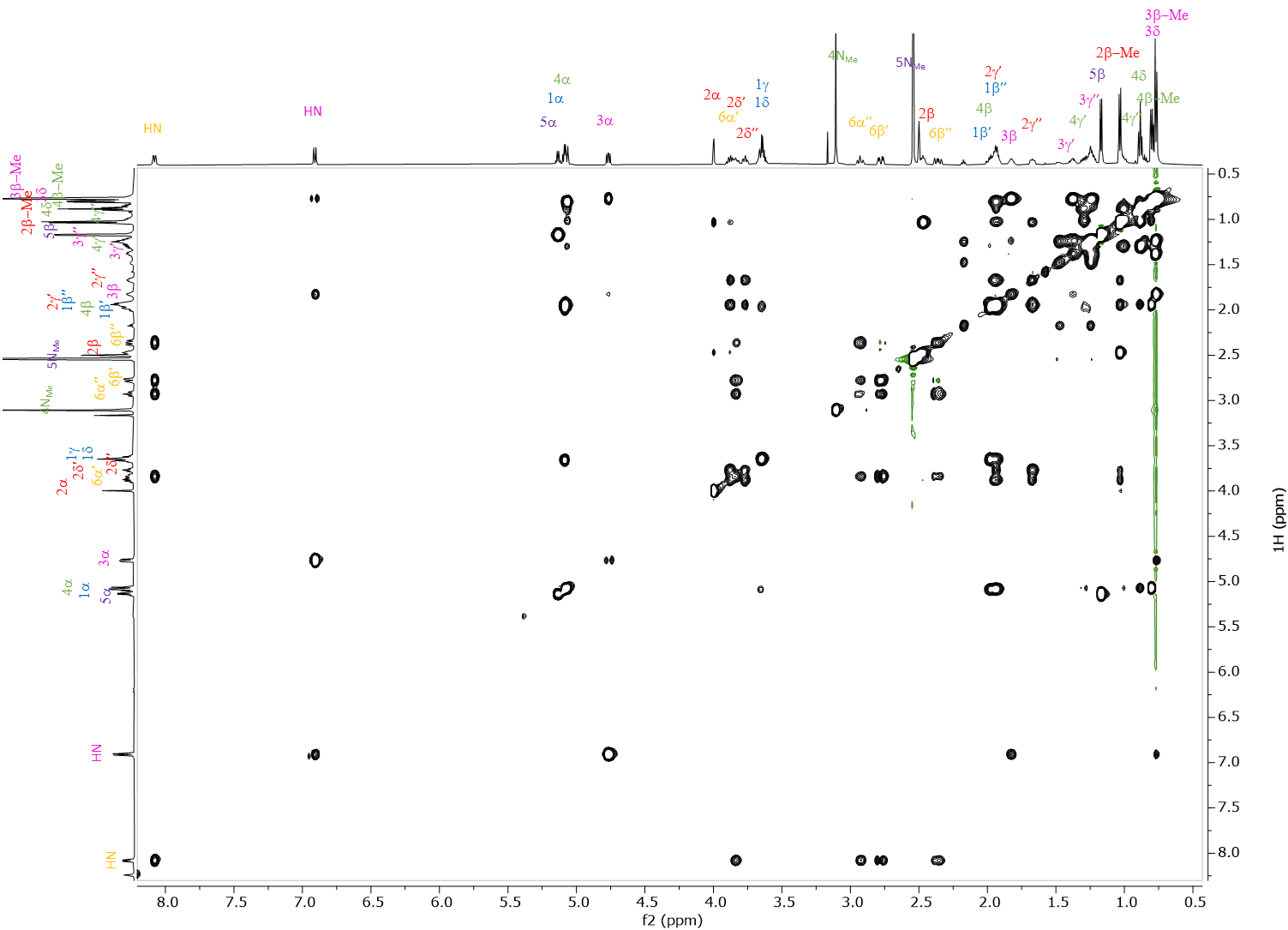


Figure S 38. TCOSY ((DIPSI2) 60ms) spectrum of **2**.


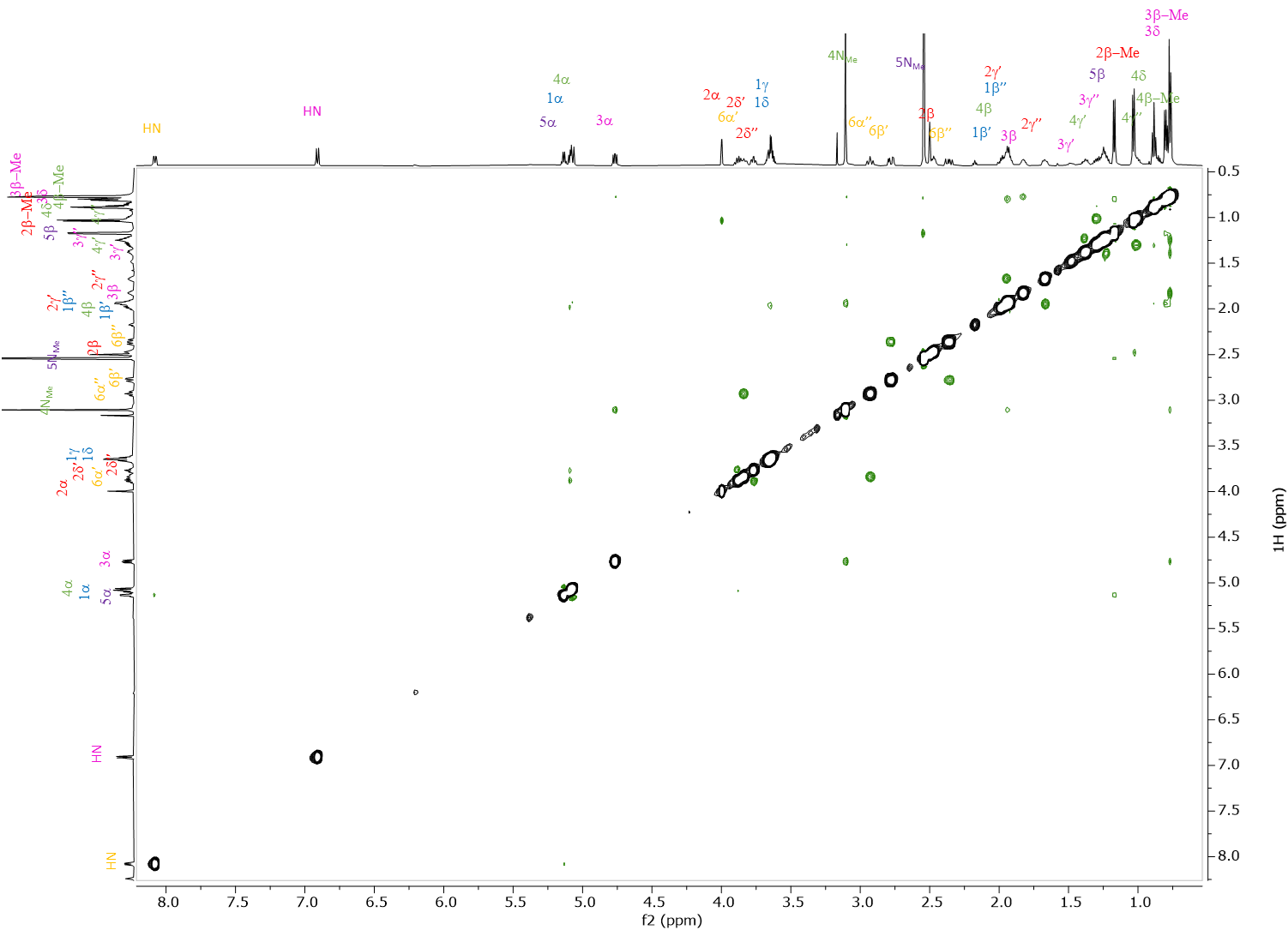


Figure S 39. ROESY (300 ms) spectrum of **2**.

**Compound 3**


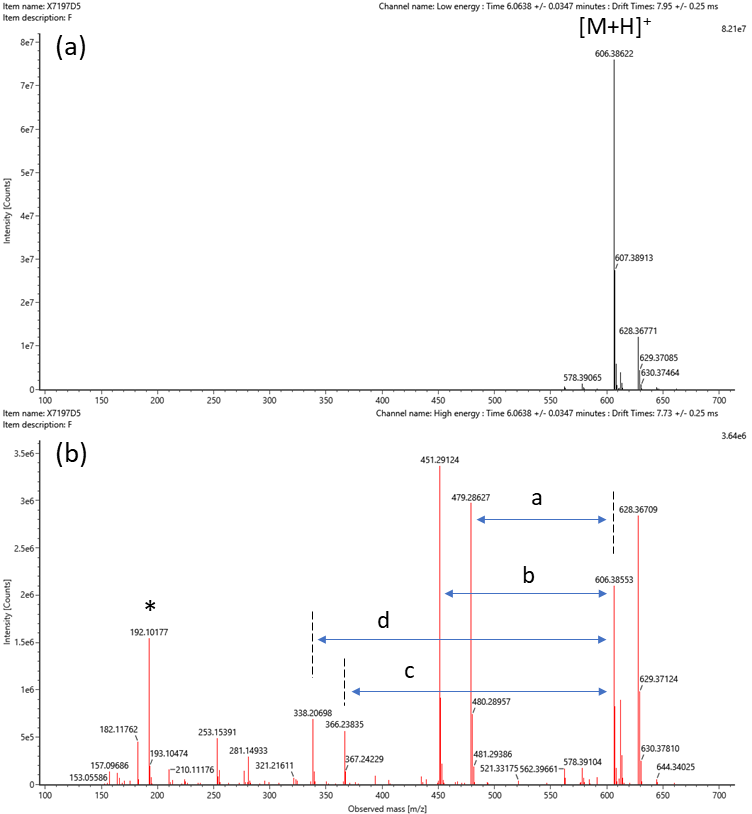


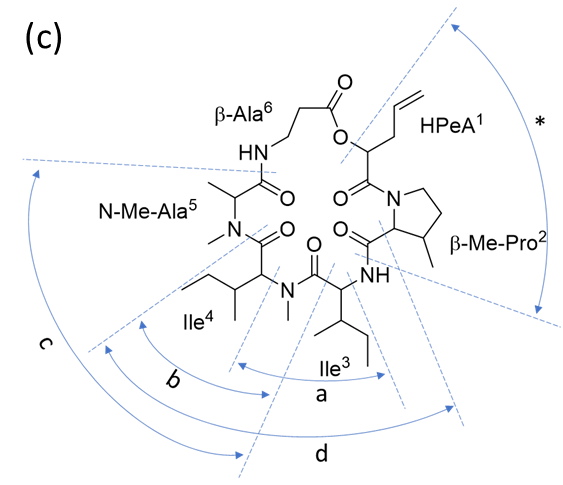


Figure S 40. HRESIMS of **3** at (a) low collision energy and (b) high collision energy in ESI+, HDMS^E^ mode. Fragmentation of **3**.


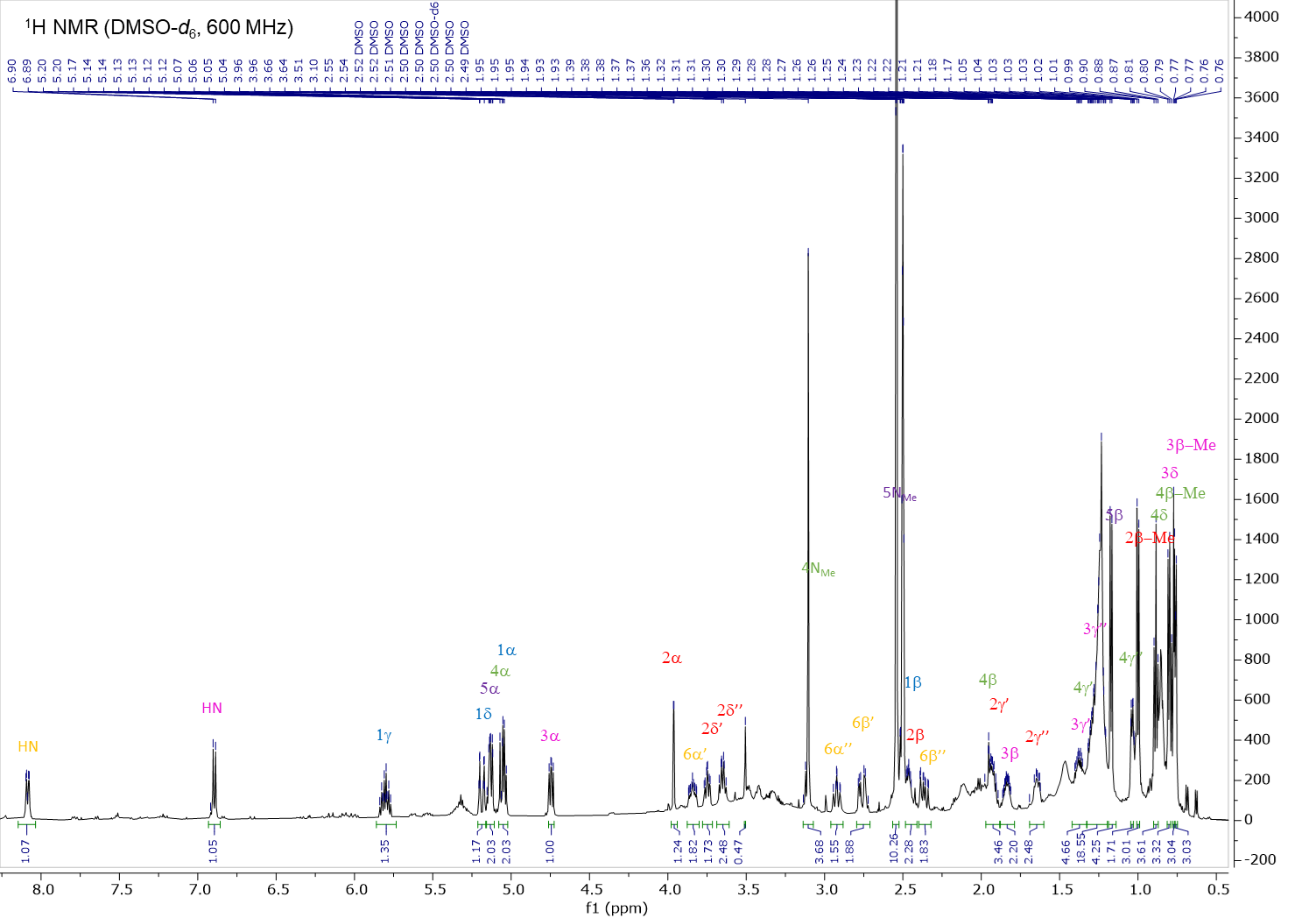


Figure S 41. ^1^H NMR spectrum of **3**.


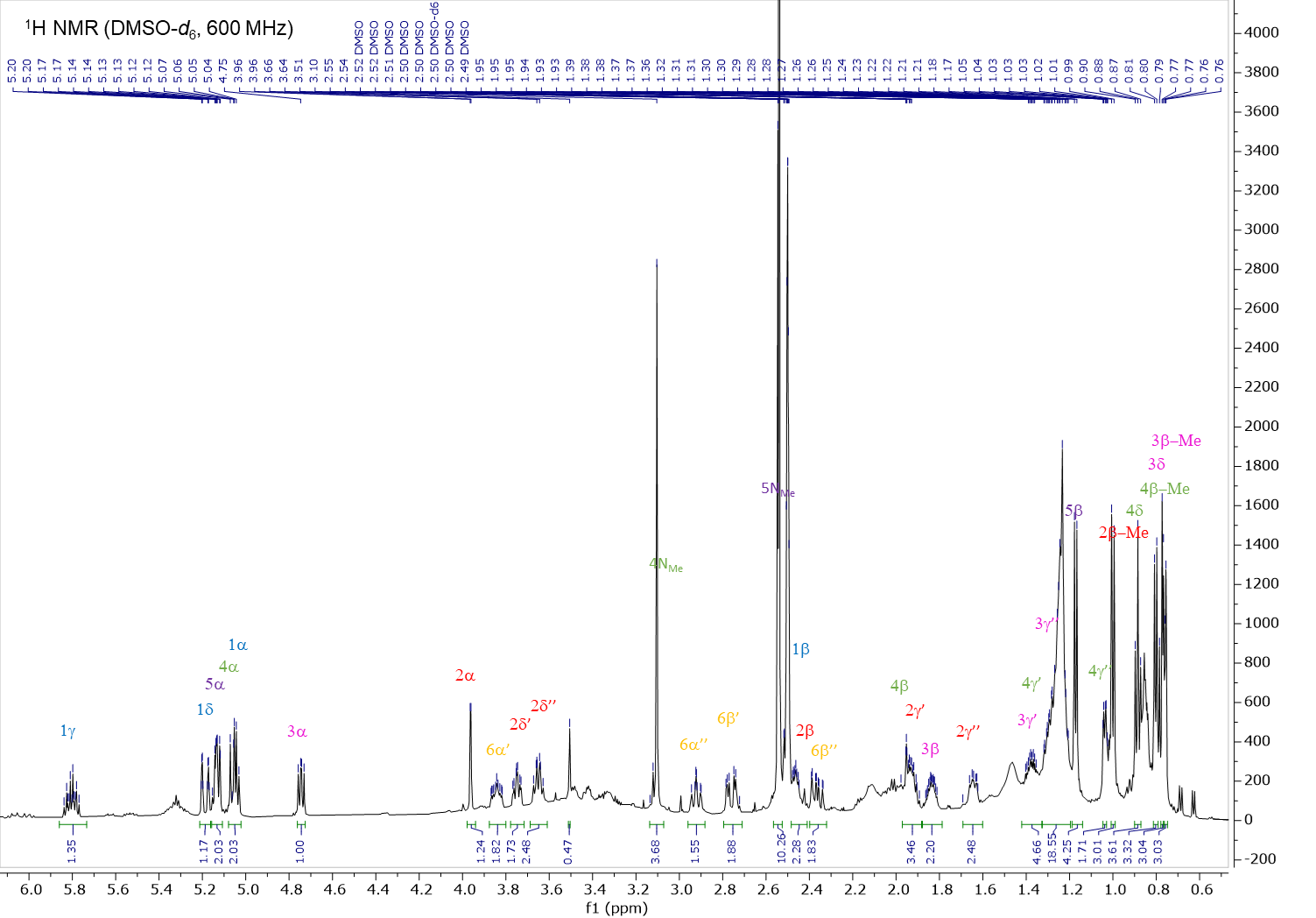


Figure S 42. ^1^H NMR (Expanded) spectrum of **3**.


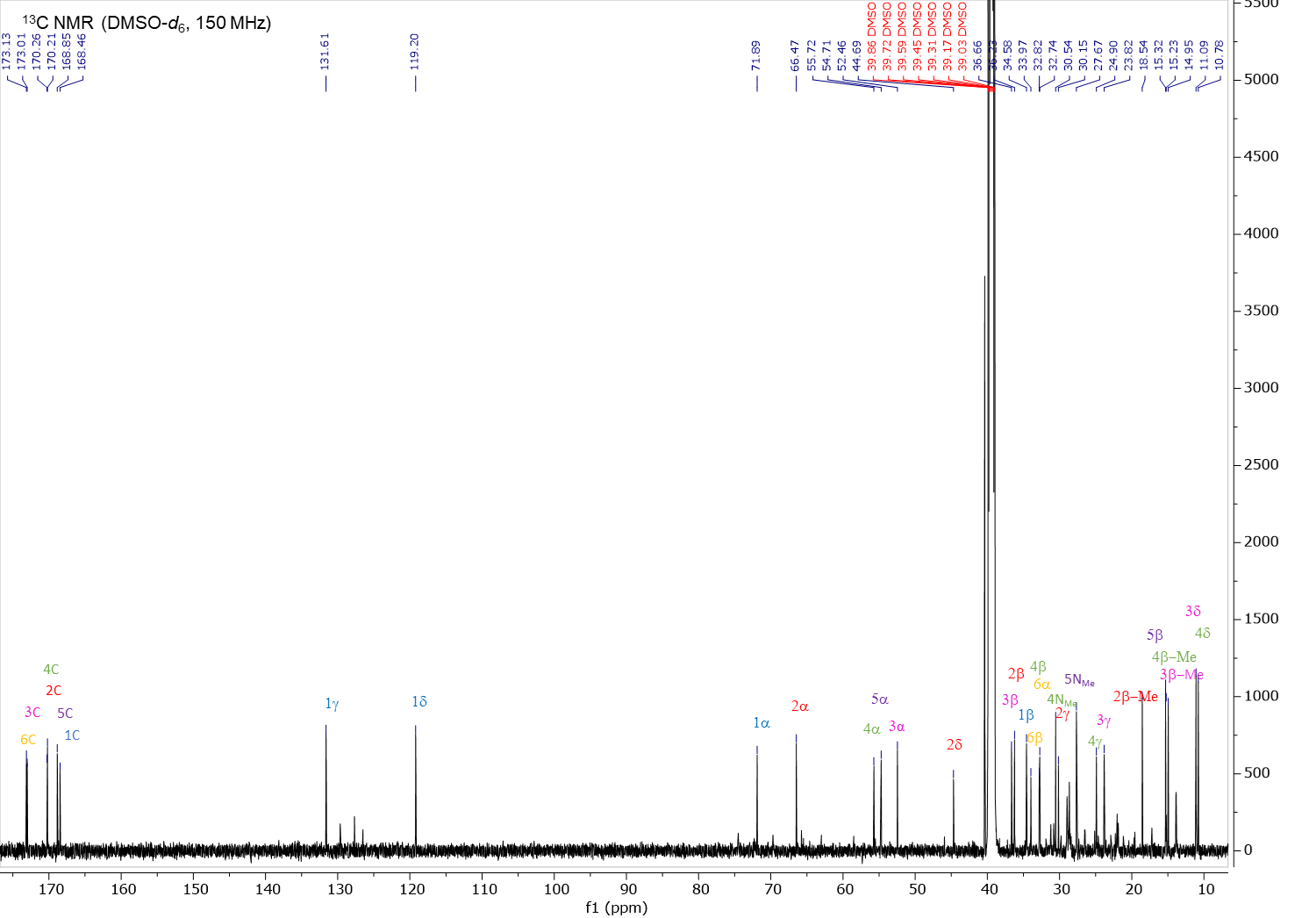


Figure S 43. ^13^C NMR spectrum of **3**.


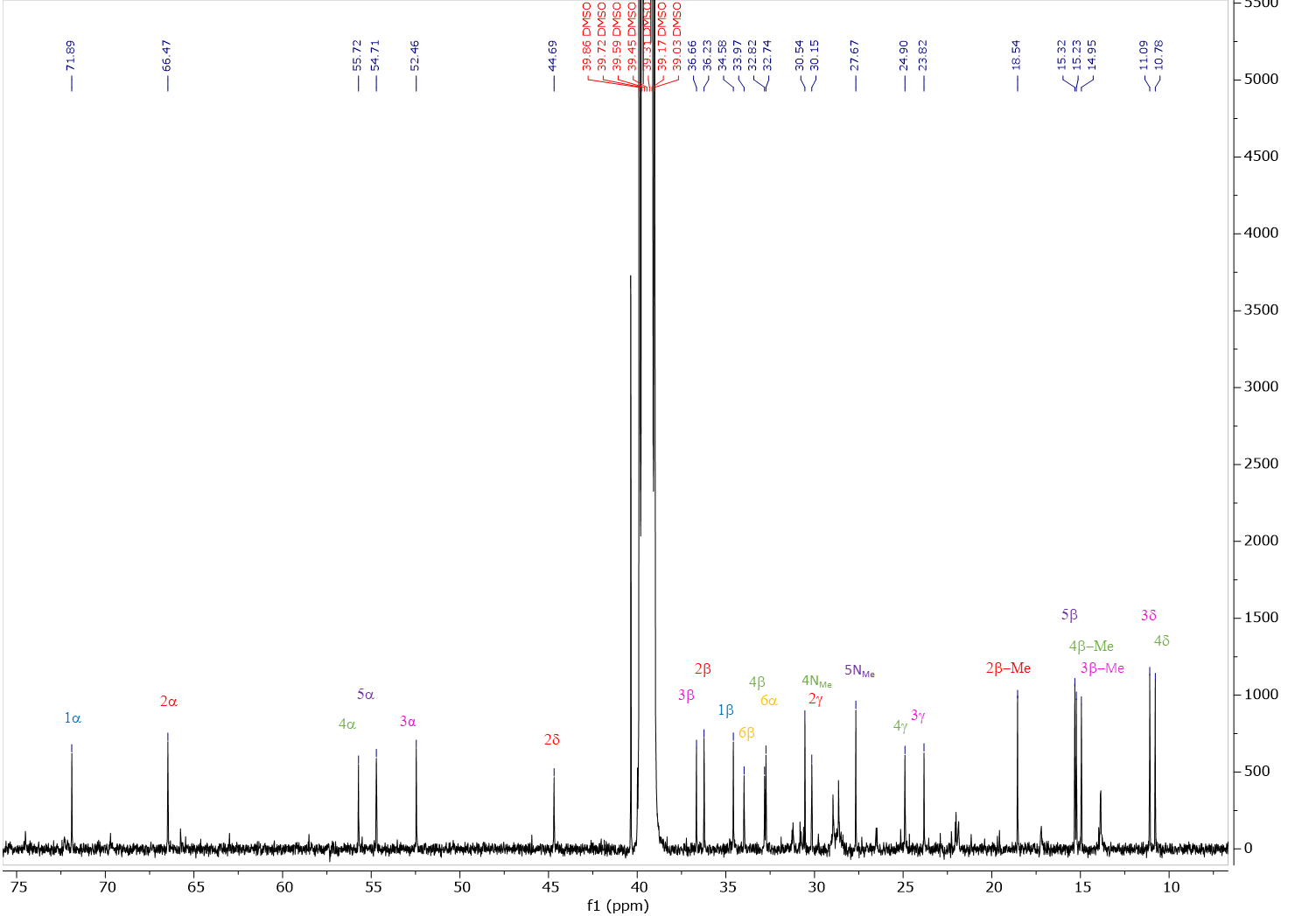


Figure S 44. ^13^C NMR (Expanded) spectrum of **3**.


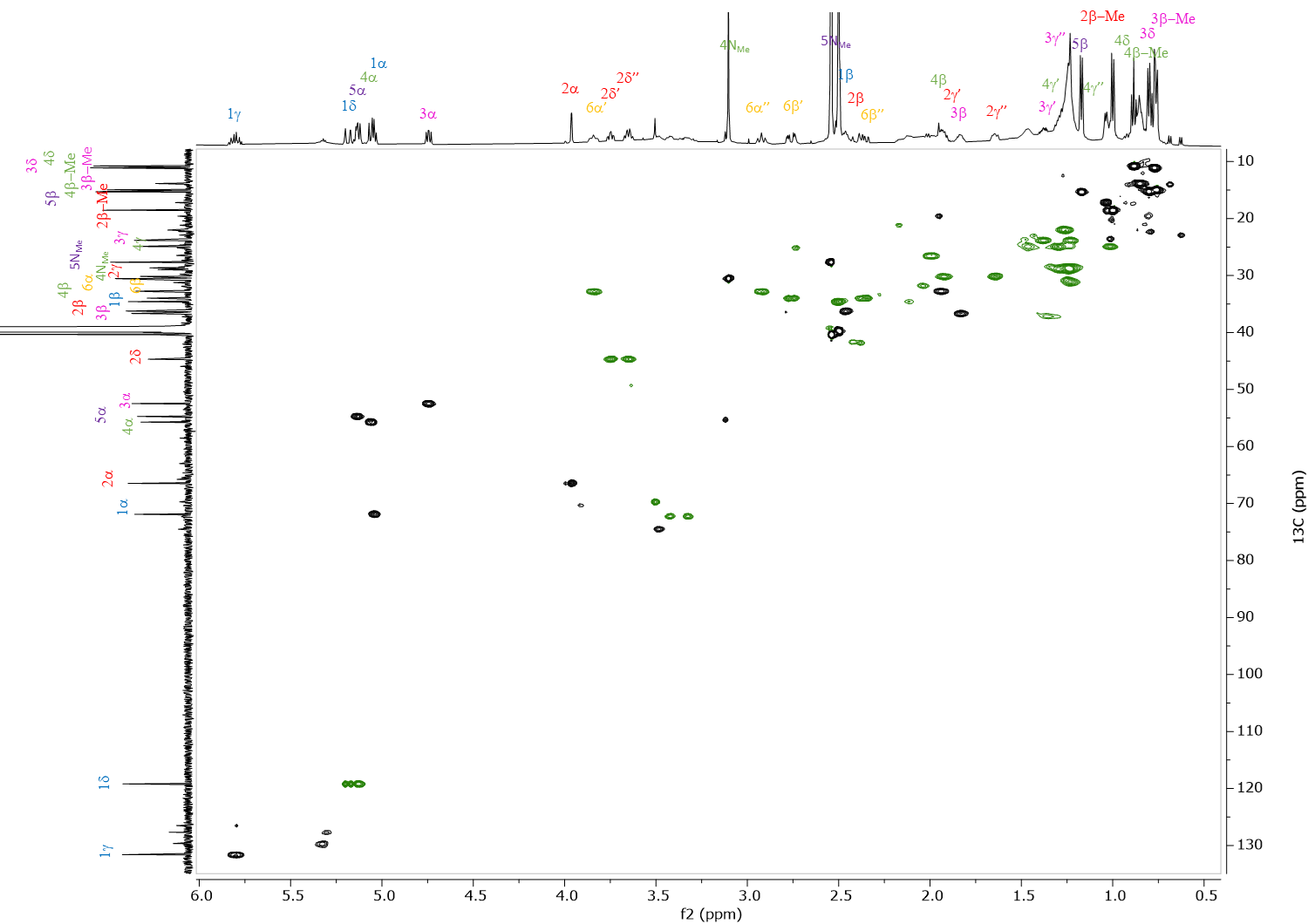


Figure S 45. HSQC spectrum of **3**.


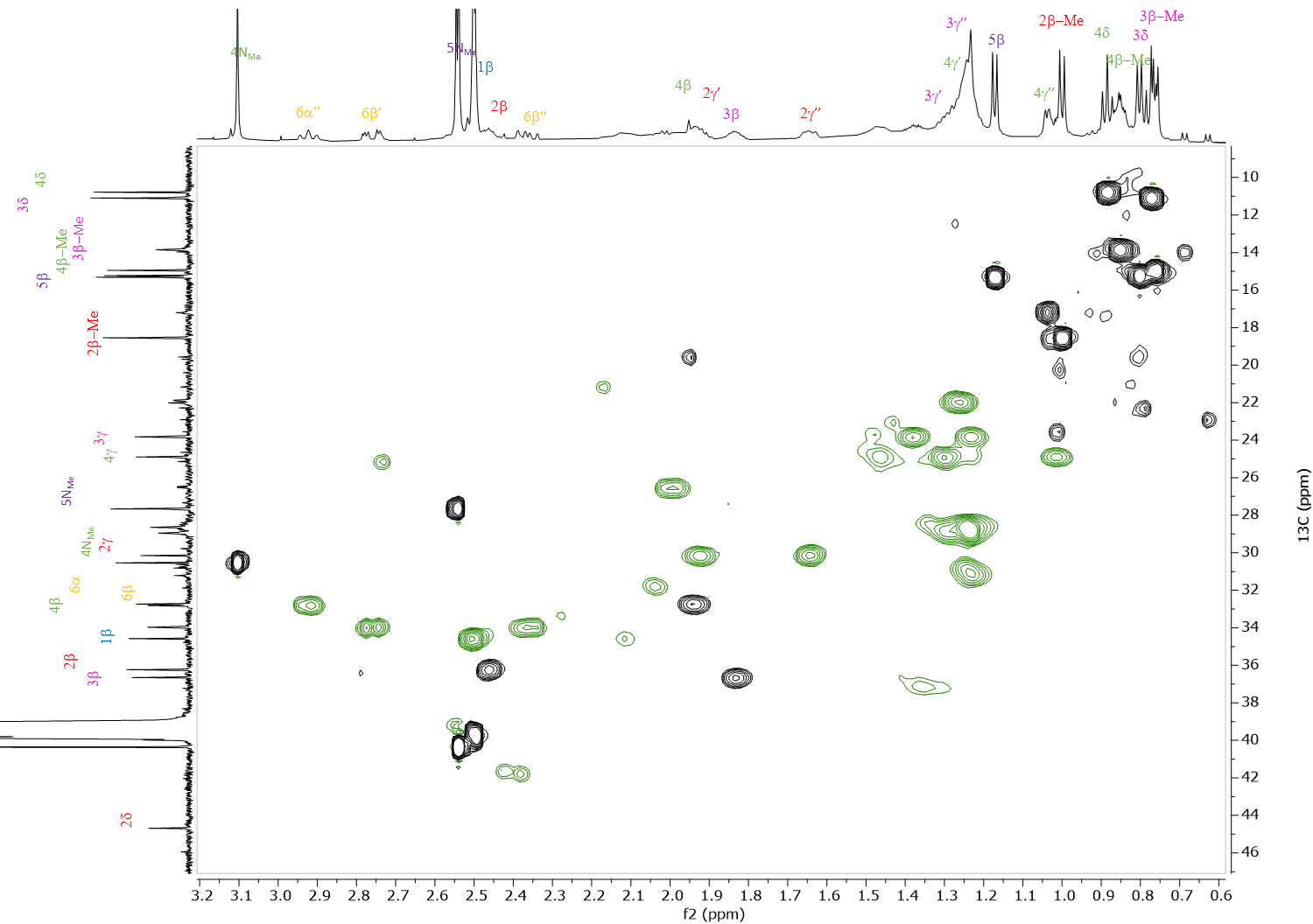


Figure S 46. HSQC (Expanded) spectrum of **3**.


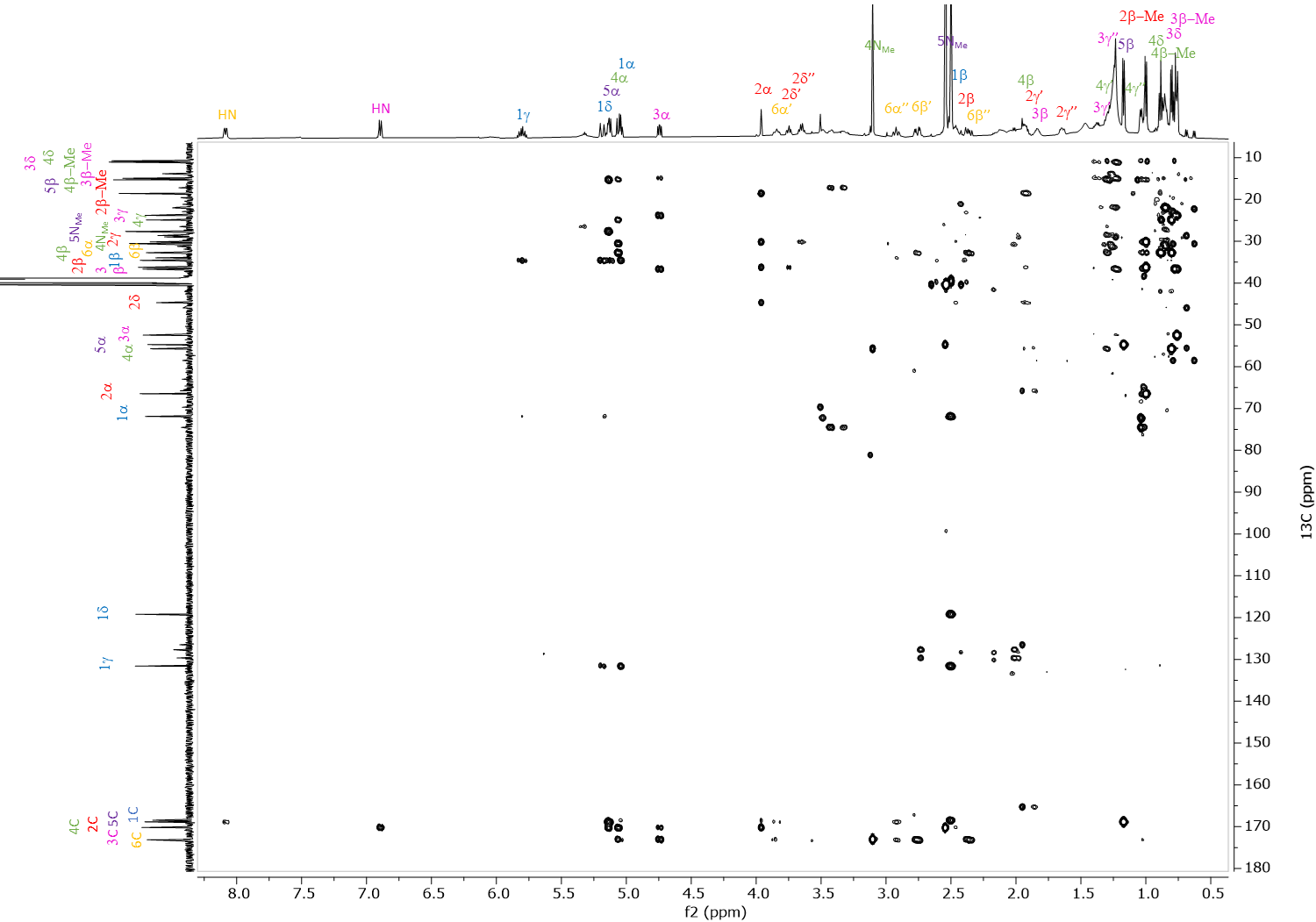


Figure S 47. HMBC of spectrum **3**.


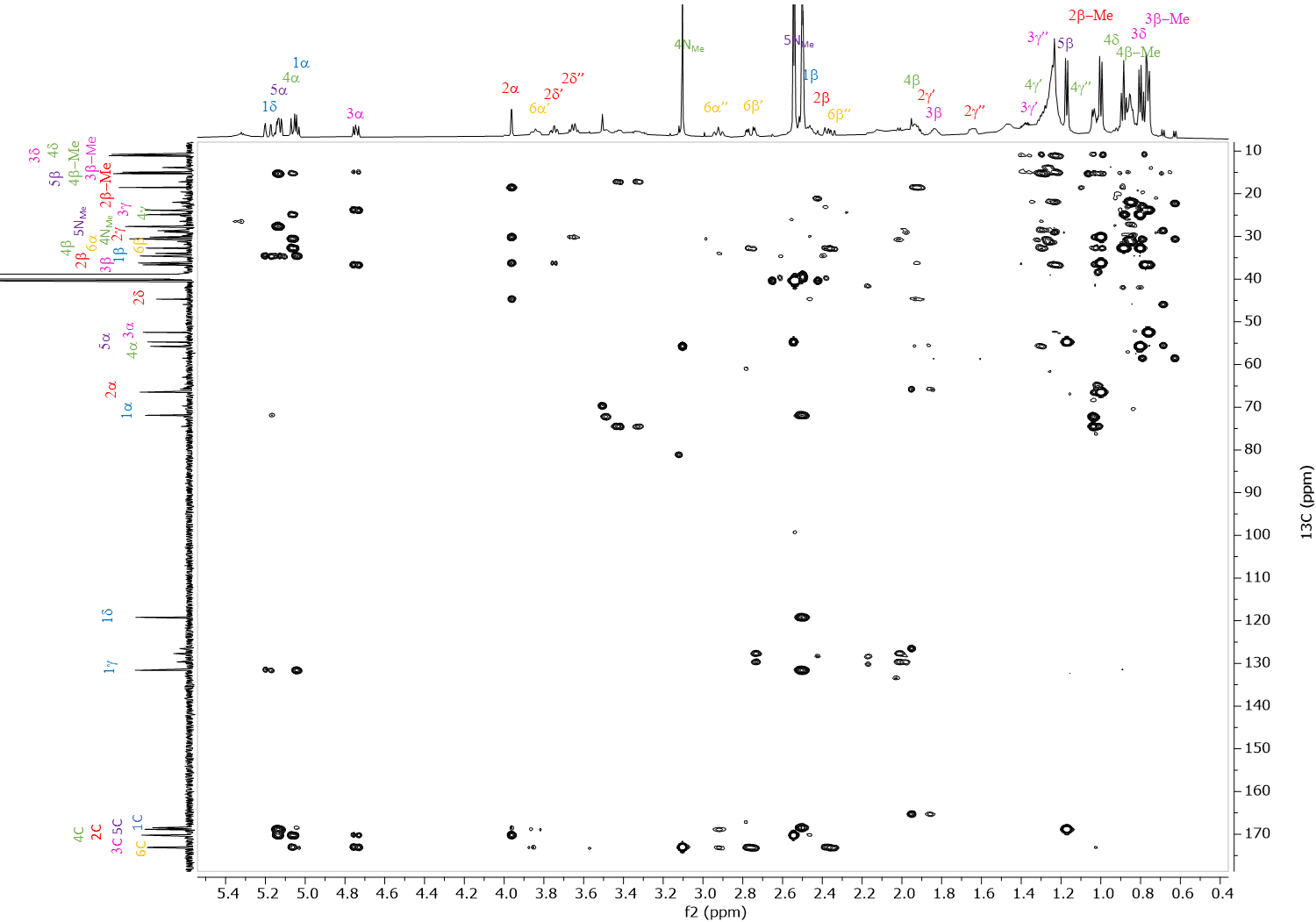


Figure S 48. HMBC (Expanded1) spectrum of **3**.


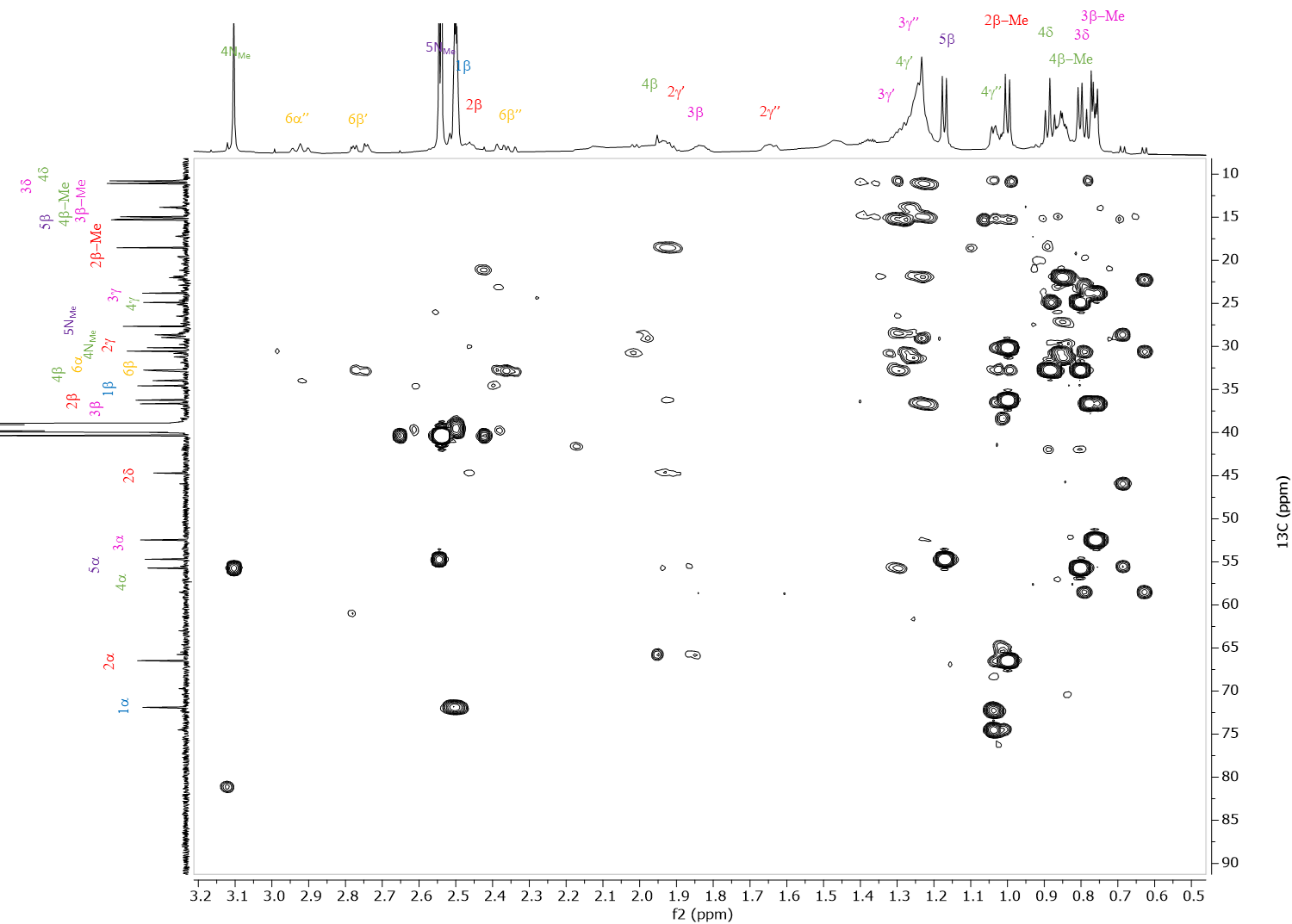


Figure S 49. HMBC (Expanded2) spectrum of **3**.


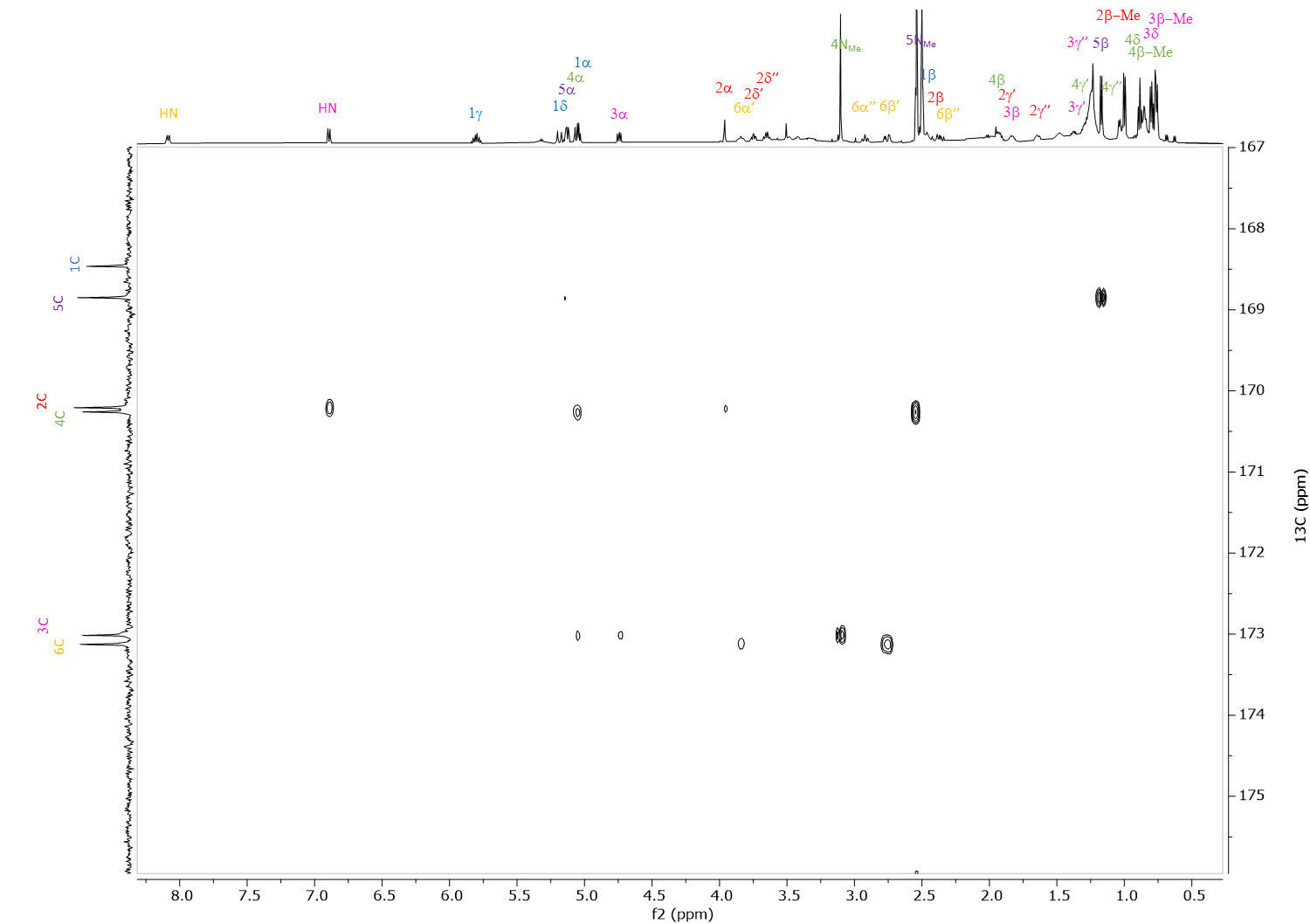


Figure S 50. Selective HMBC (8 Hz) spectrum of **3**.


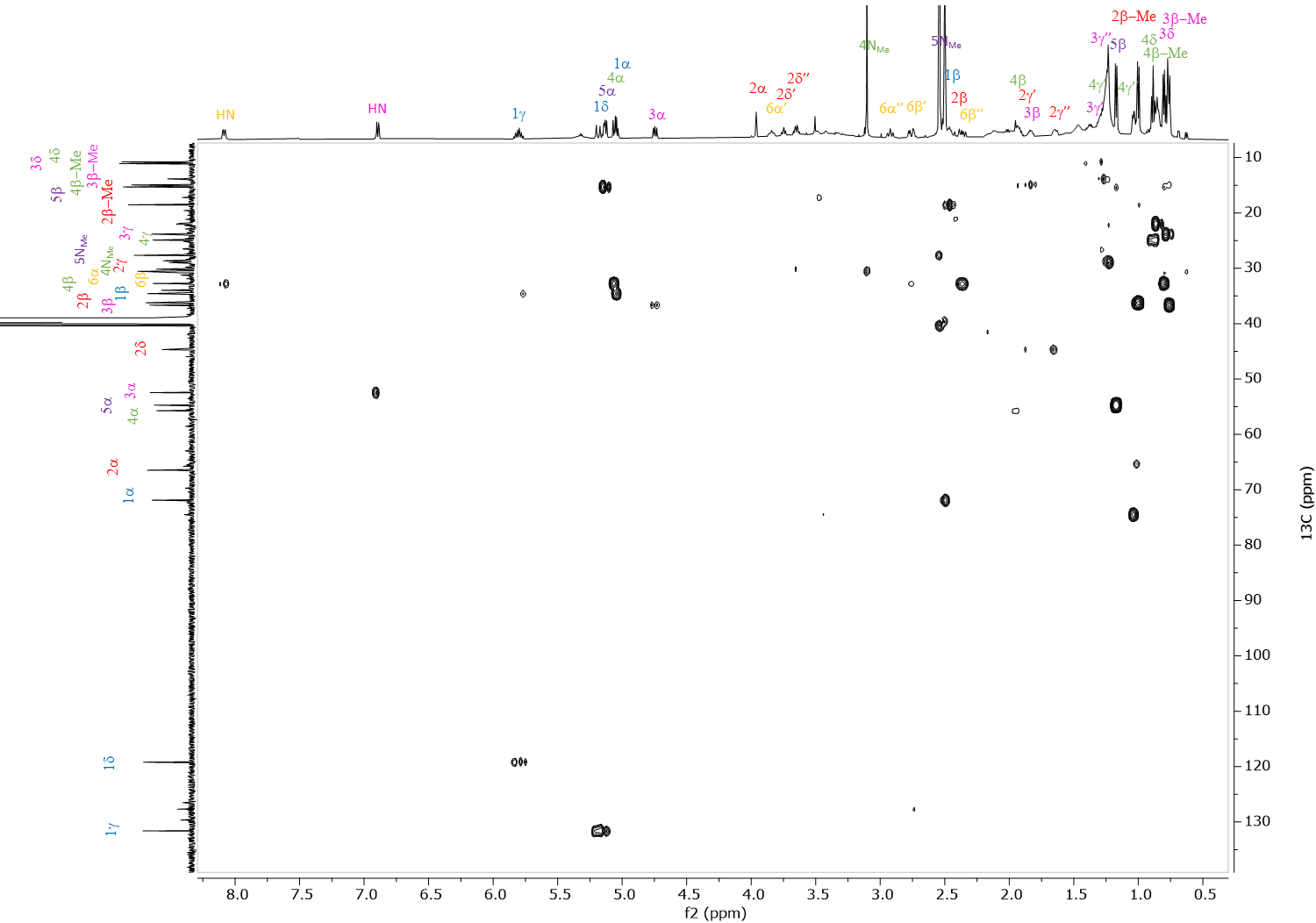


Figure S 51. H2BC spectrum of **3**.


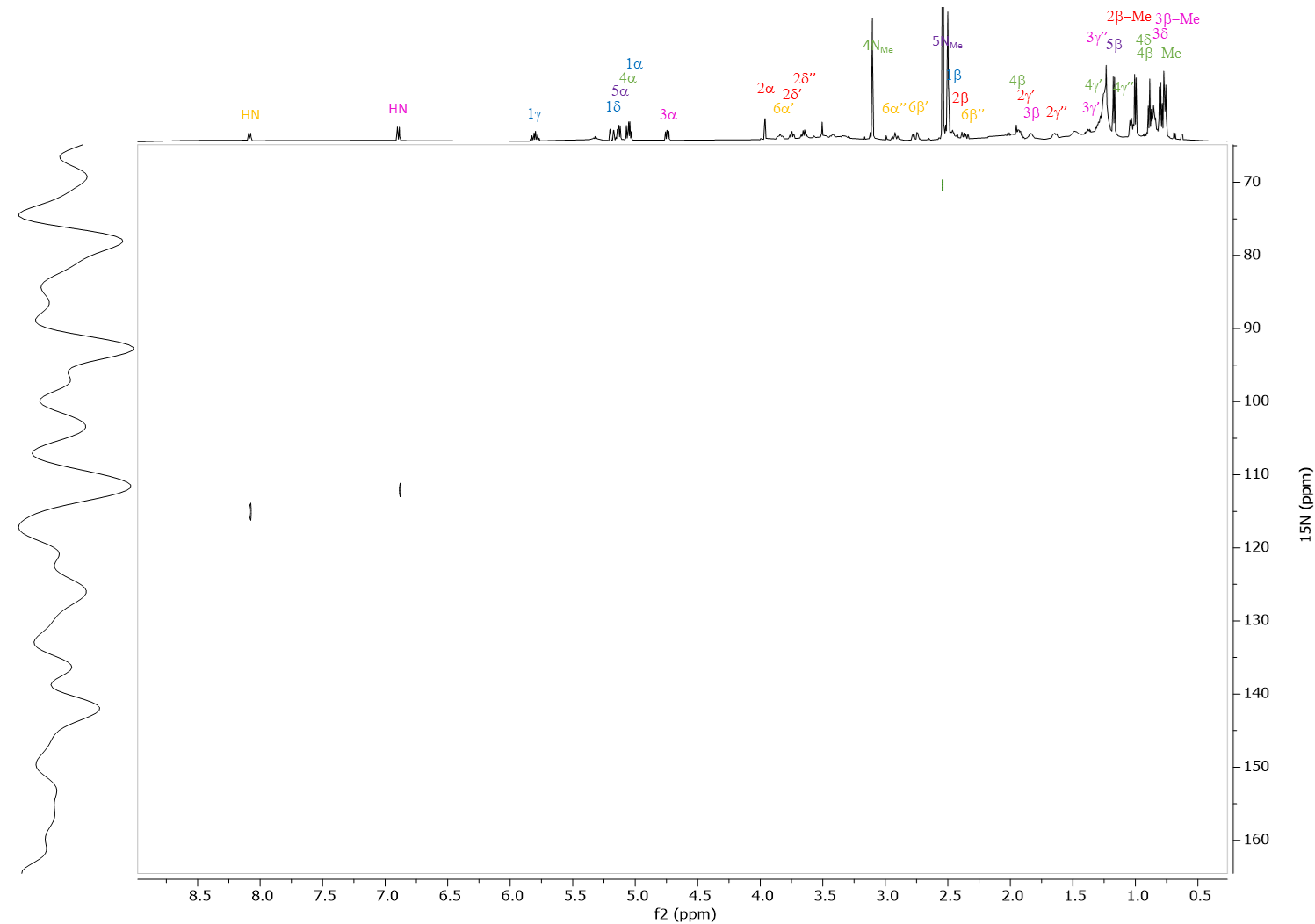


Figure S 52. ^1^H,^15^N-HSQC spectrum of **3**.


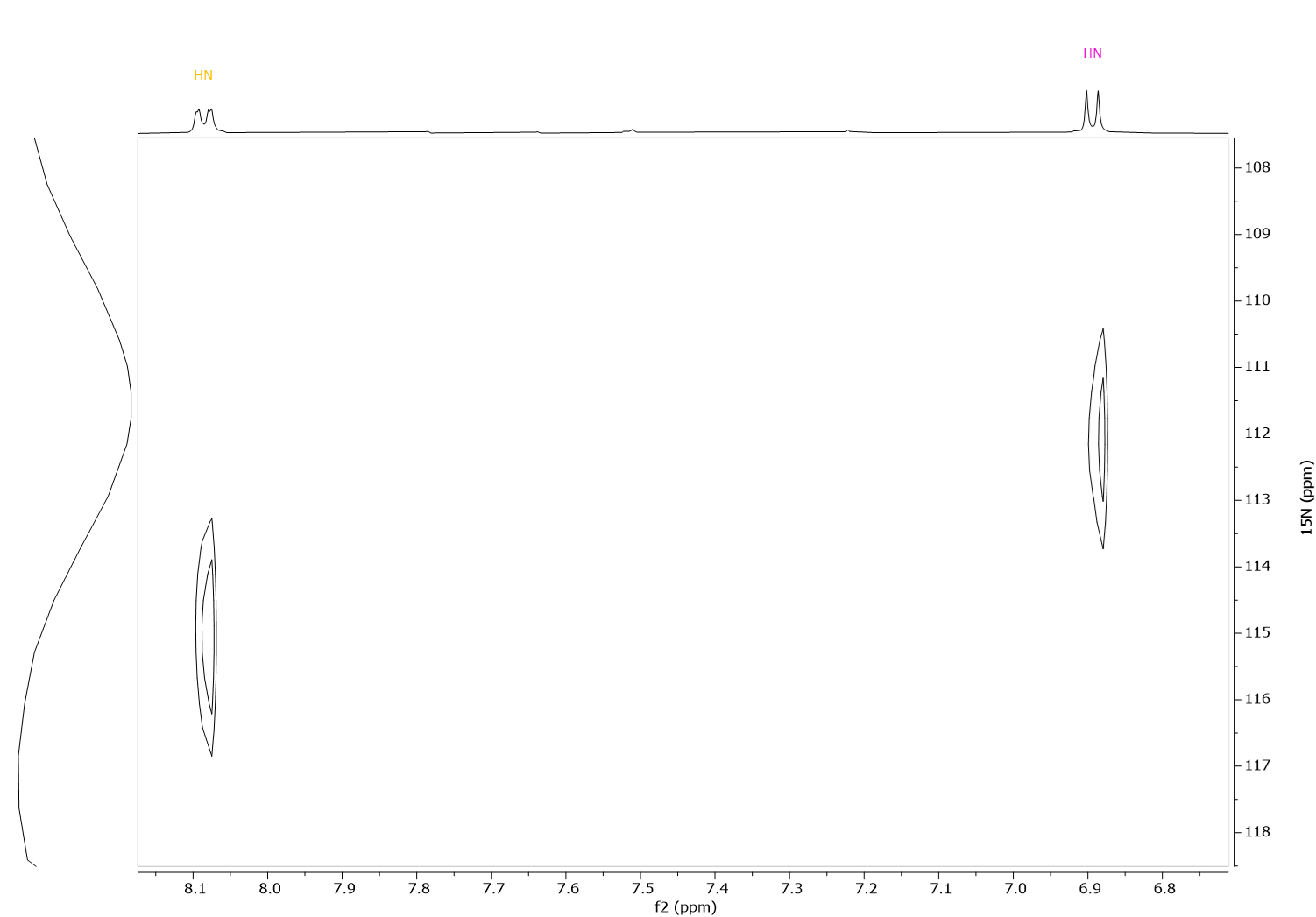


Figure S 53. ^1^H,^15^N-HSQC (Expanded) spectrum of **3**.


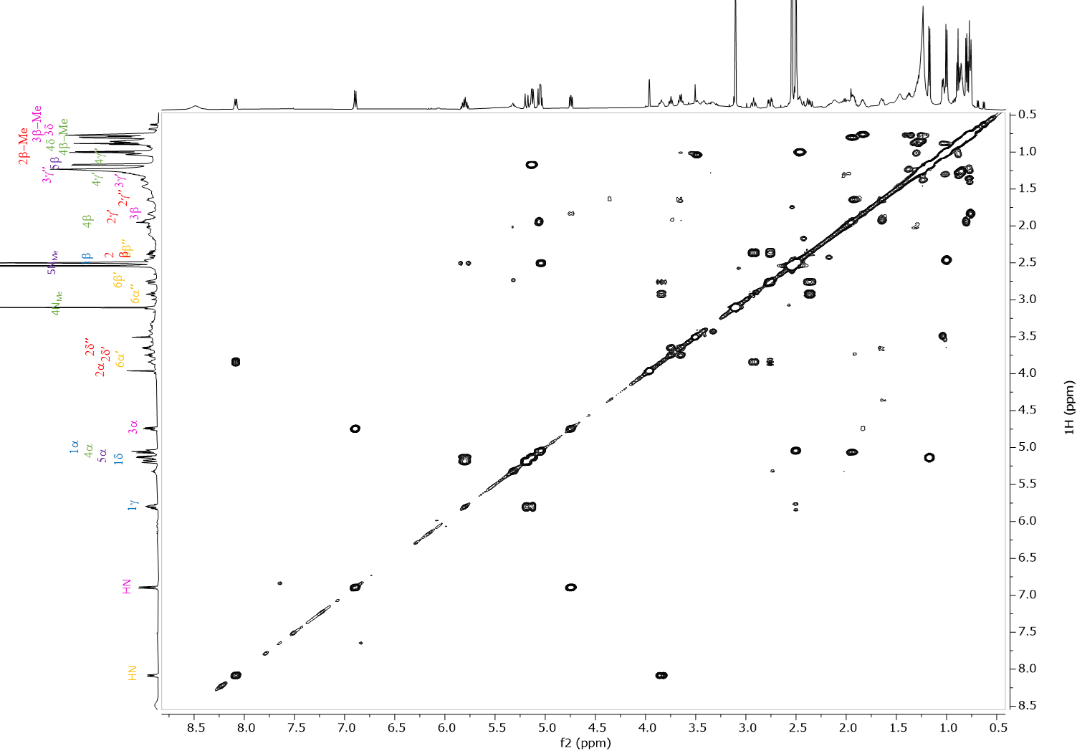


Figure S 54. COSY spectrum of **3**.


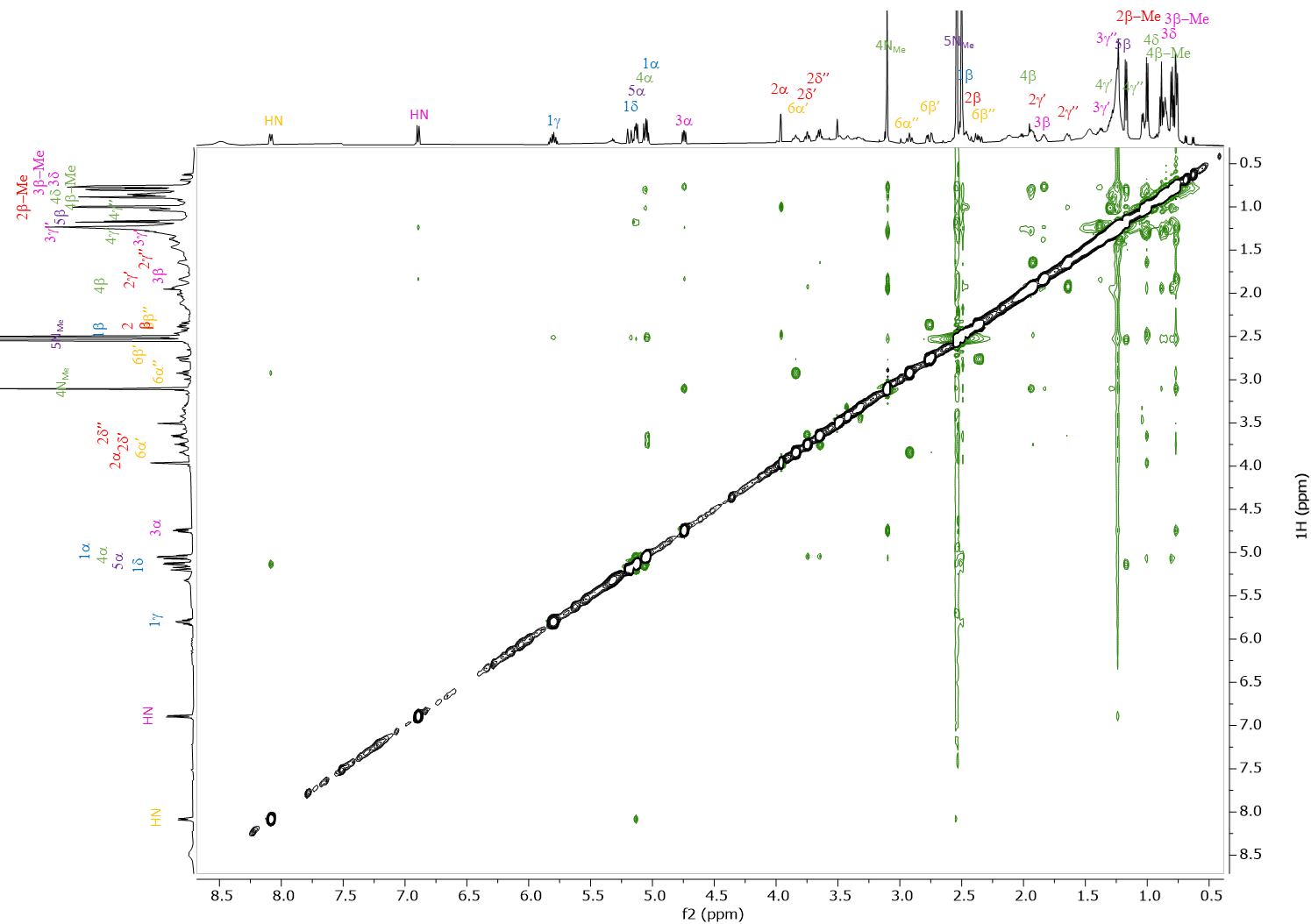


Figure S 55. ROESY (300 ms) spectrum of **3**.


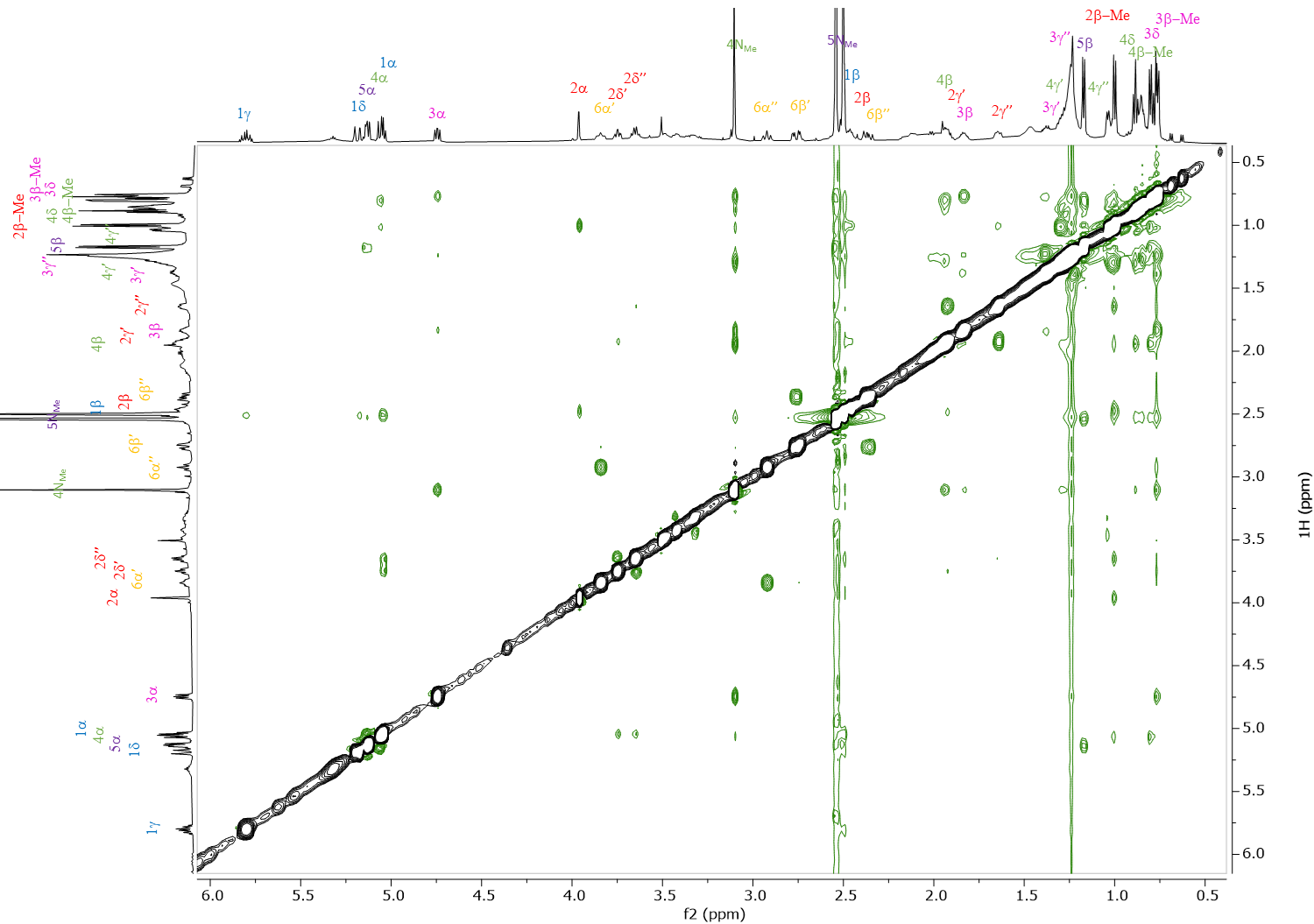


Figure S 56. ROESY (Expanded) spectrum of **3**.

**Compound 4**


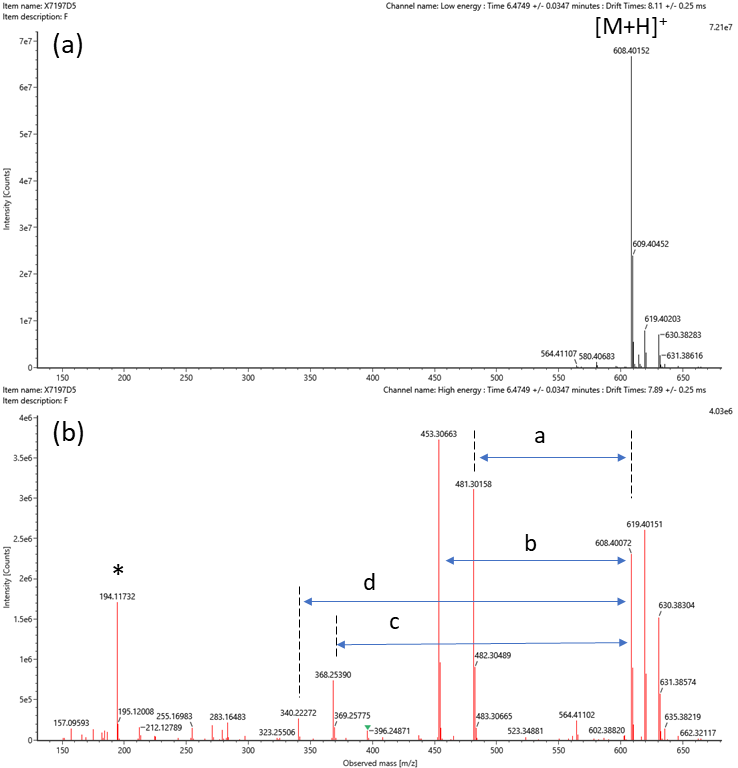


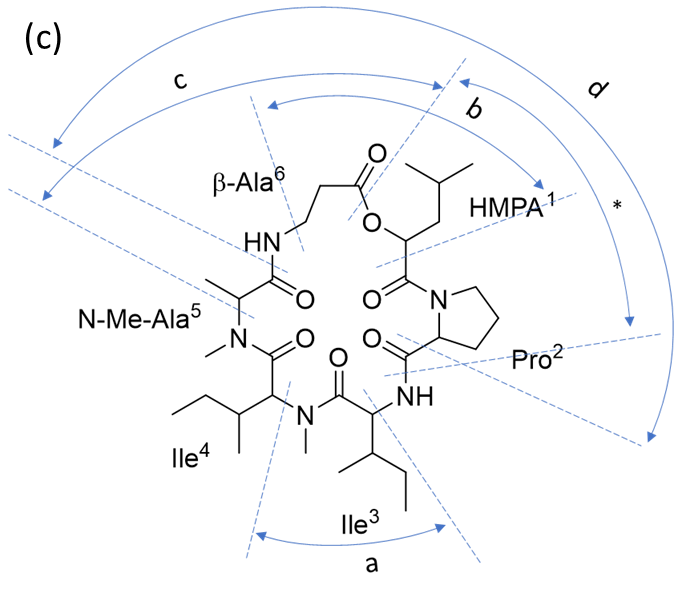


Figure S 57. HRESIMS of **4** at (a) low collision energy and (b) high collision energy in ESI+, HDMS^E^ mode. Fragmentation of **4**.


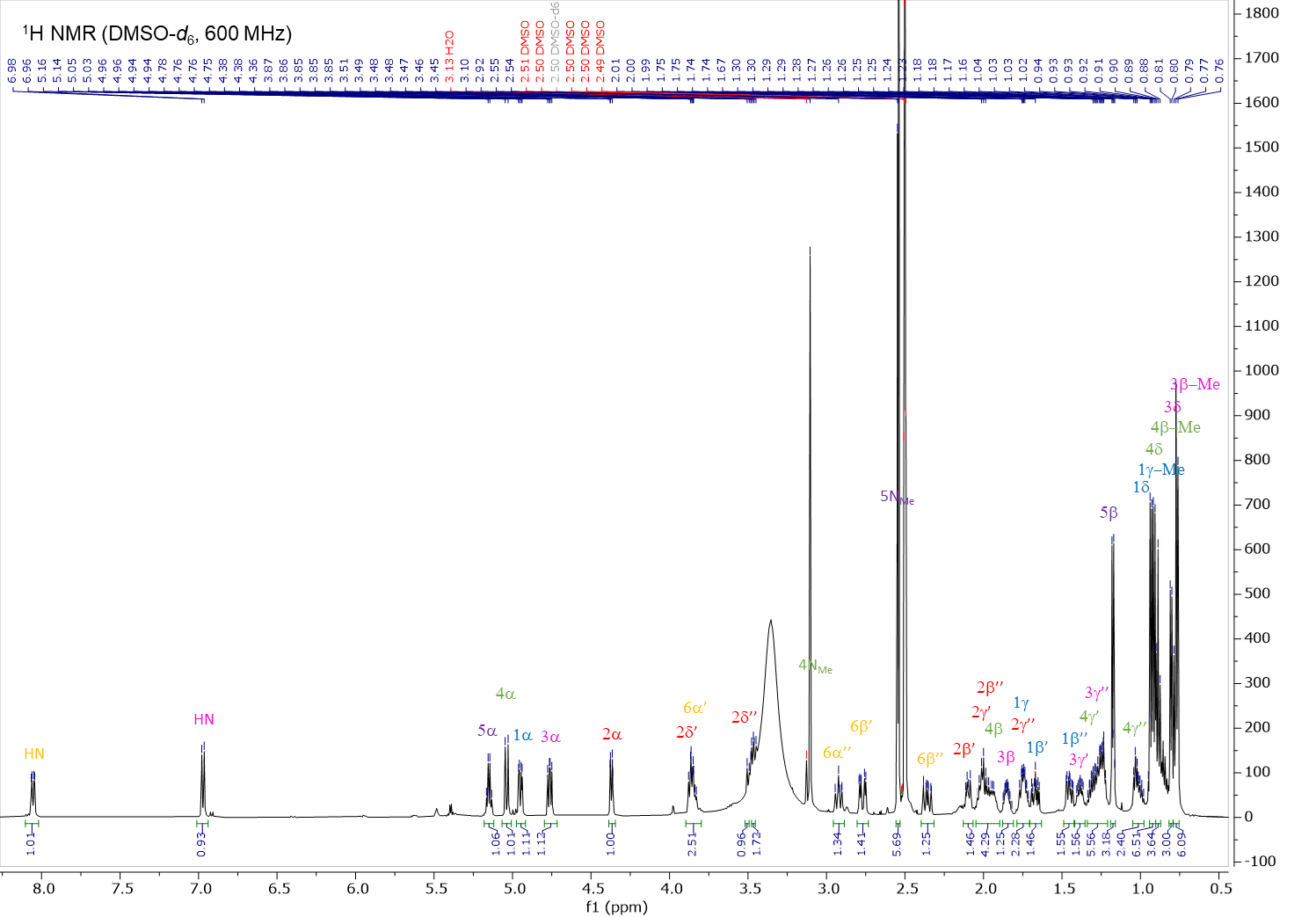


Figure S 58. ^1^H NMR spectrum of **4**.


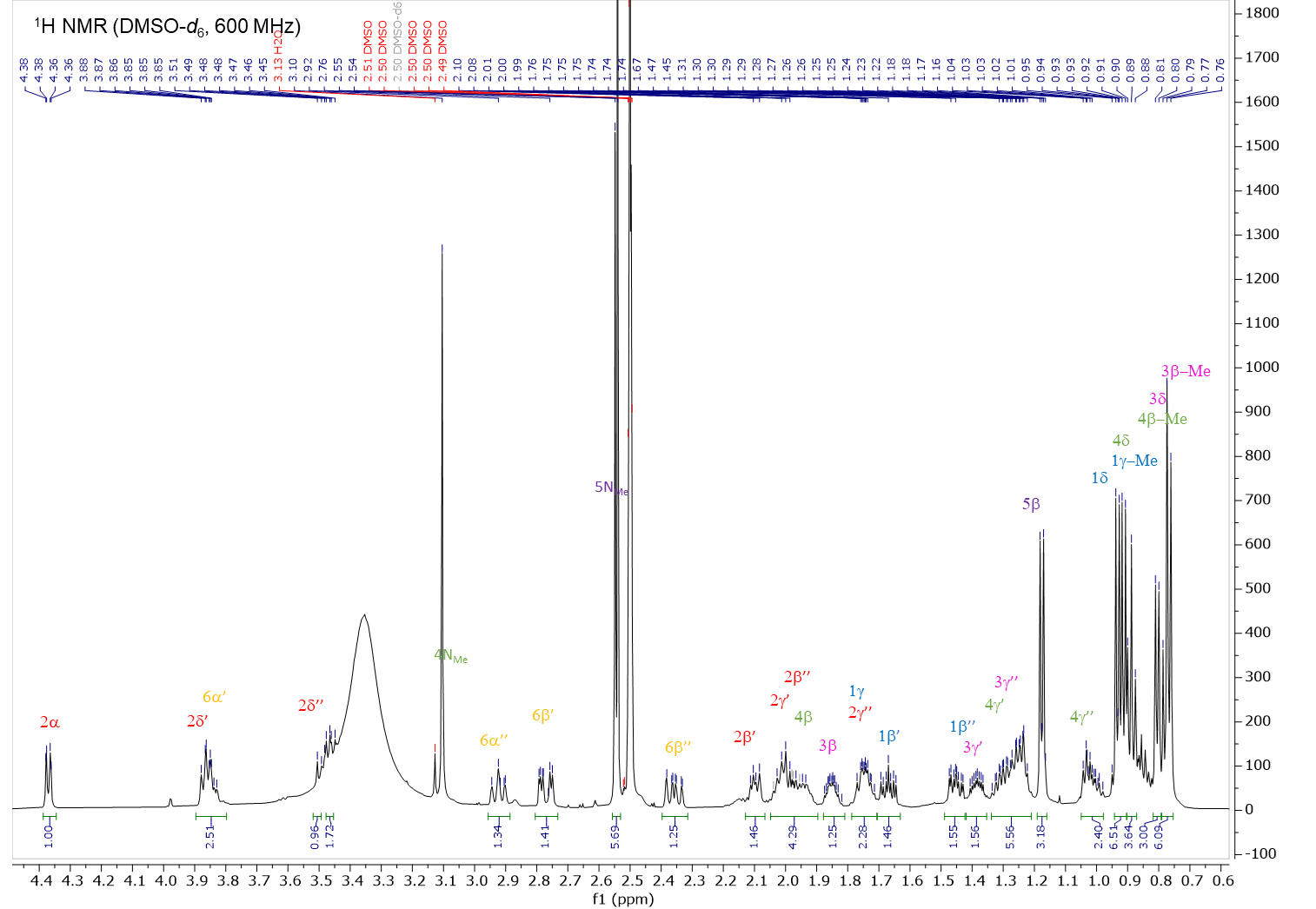


Figure S 59. ^1^H NMR (Expanded) spectrum of **4**.


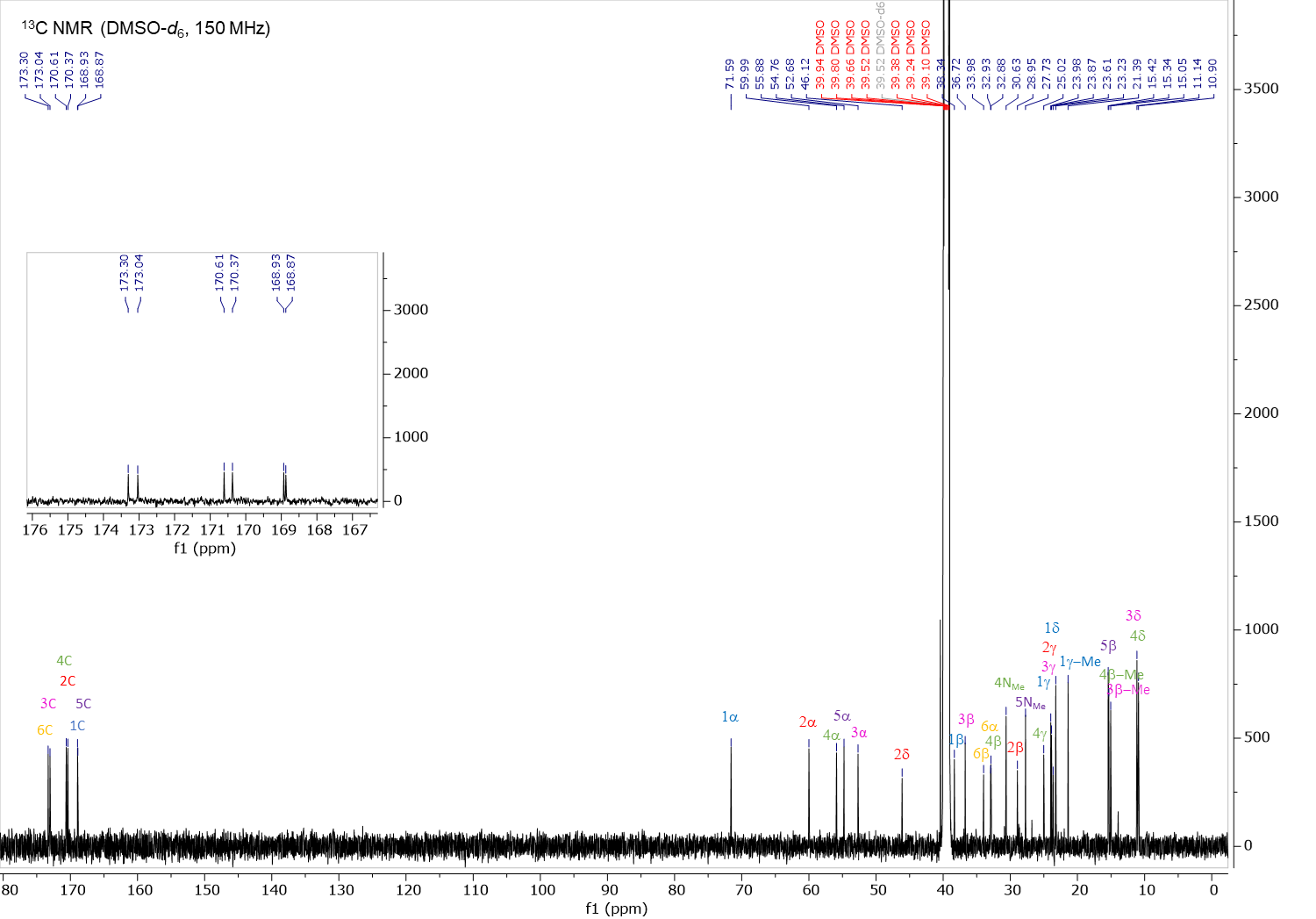


Figure S 60. ^13^C NMR spectrum of **4**.


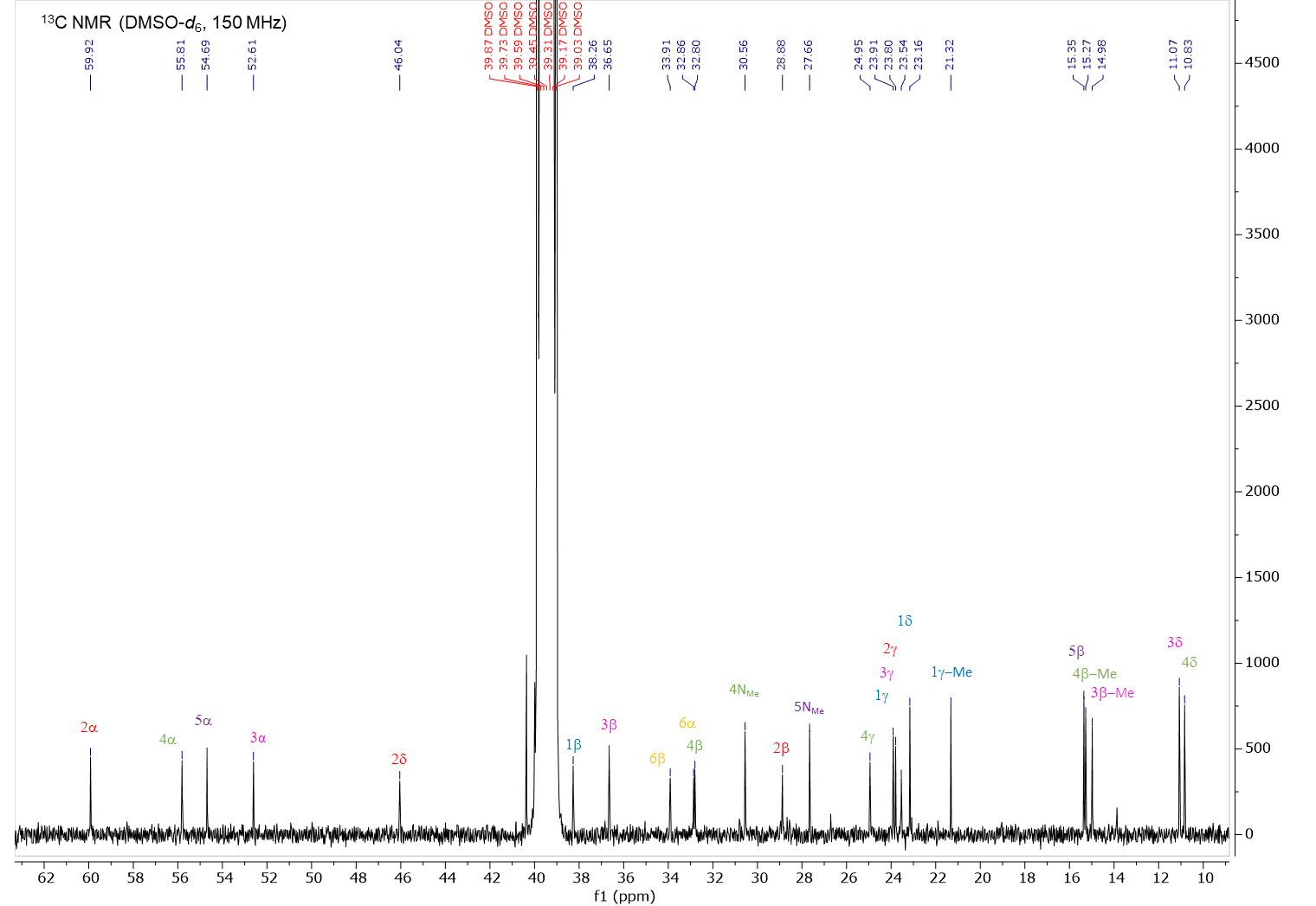


Figure S 61. ^13^C NMR (Expanded) spectrum of **4**.


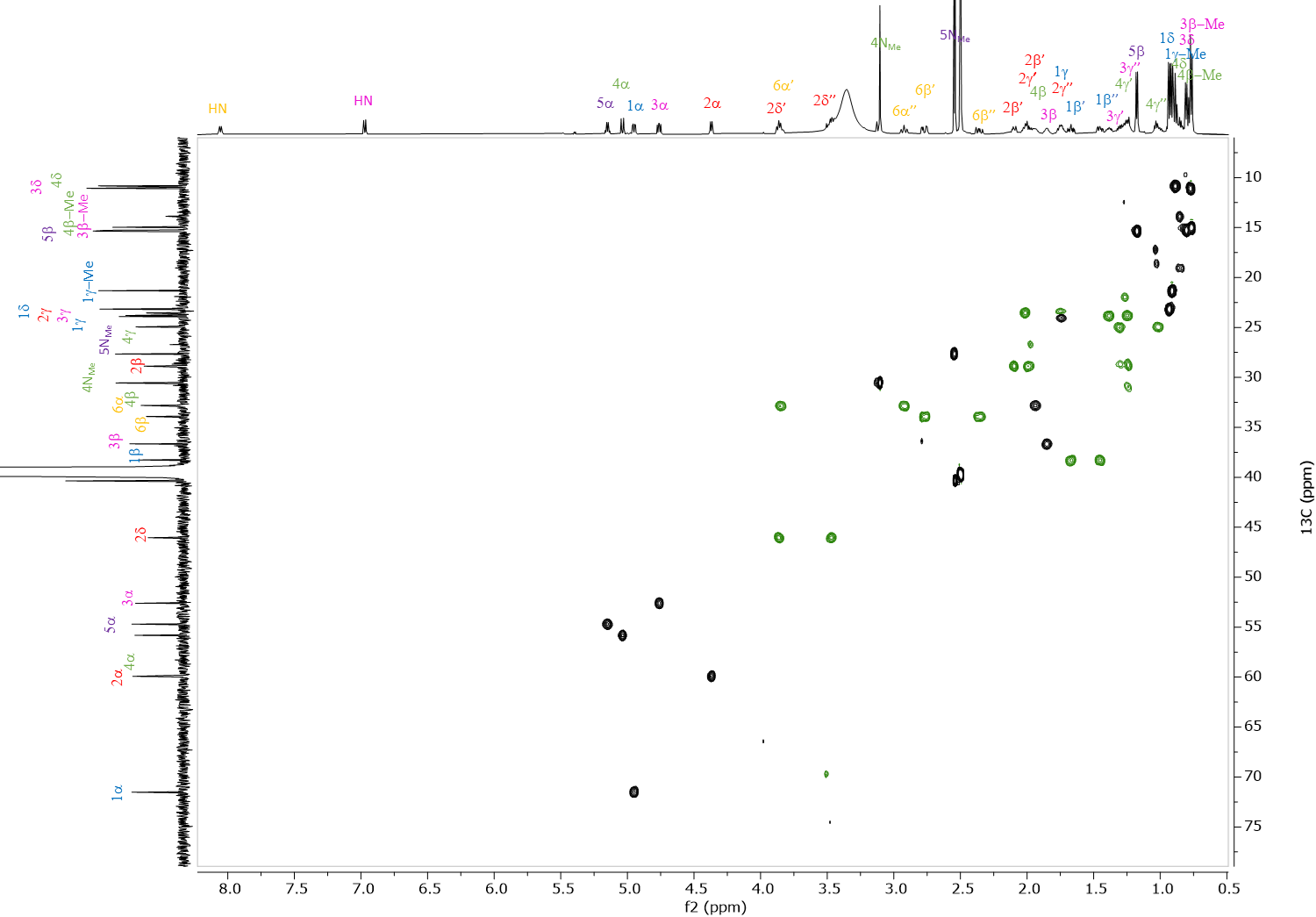


Figure S 62. HSQC spectrum of **4**.


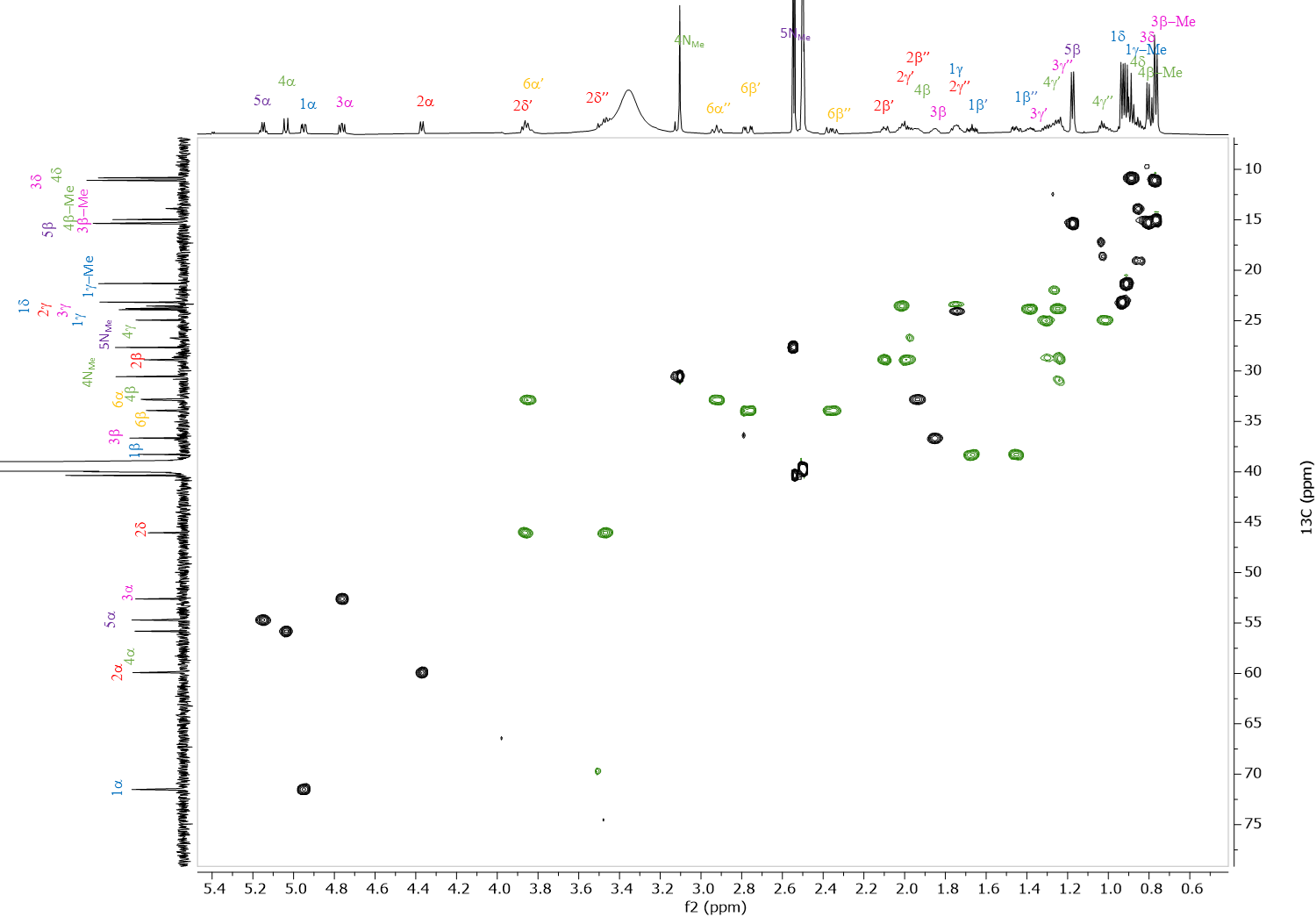


Figure S 63. HSQC (Expanded) spectrum of **4**.


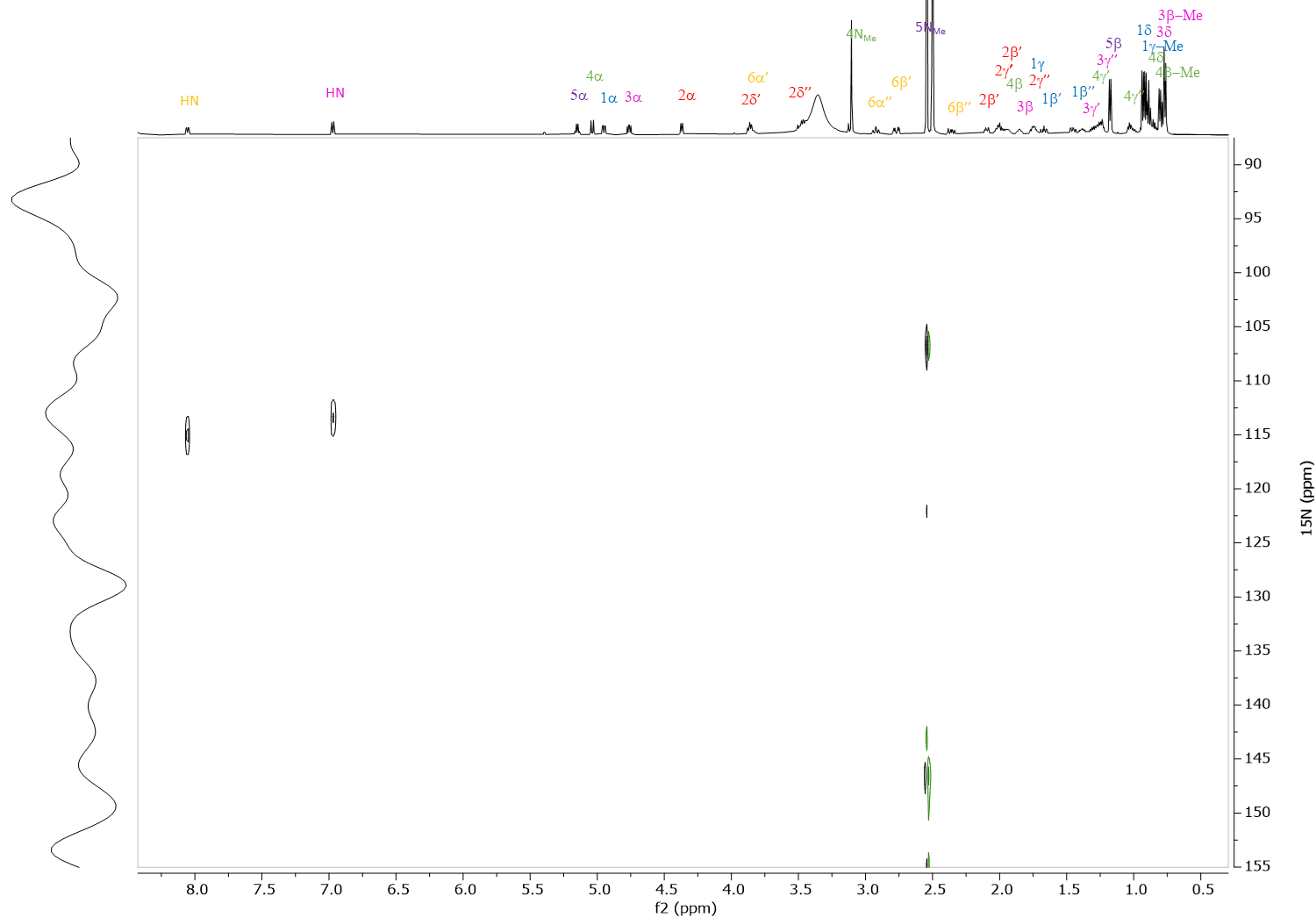


Figure S 64. ^1^H,^15^N-HSQC spectrum of **4**.


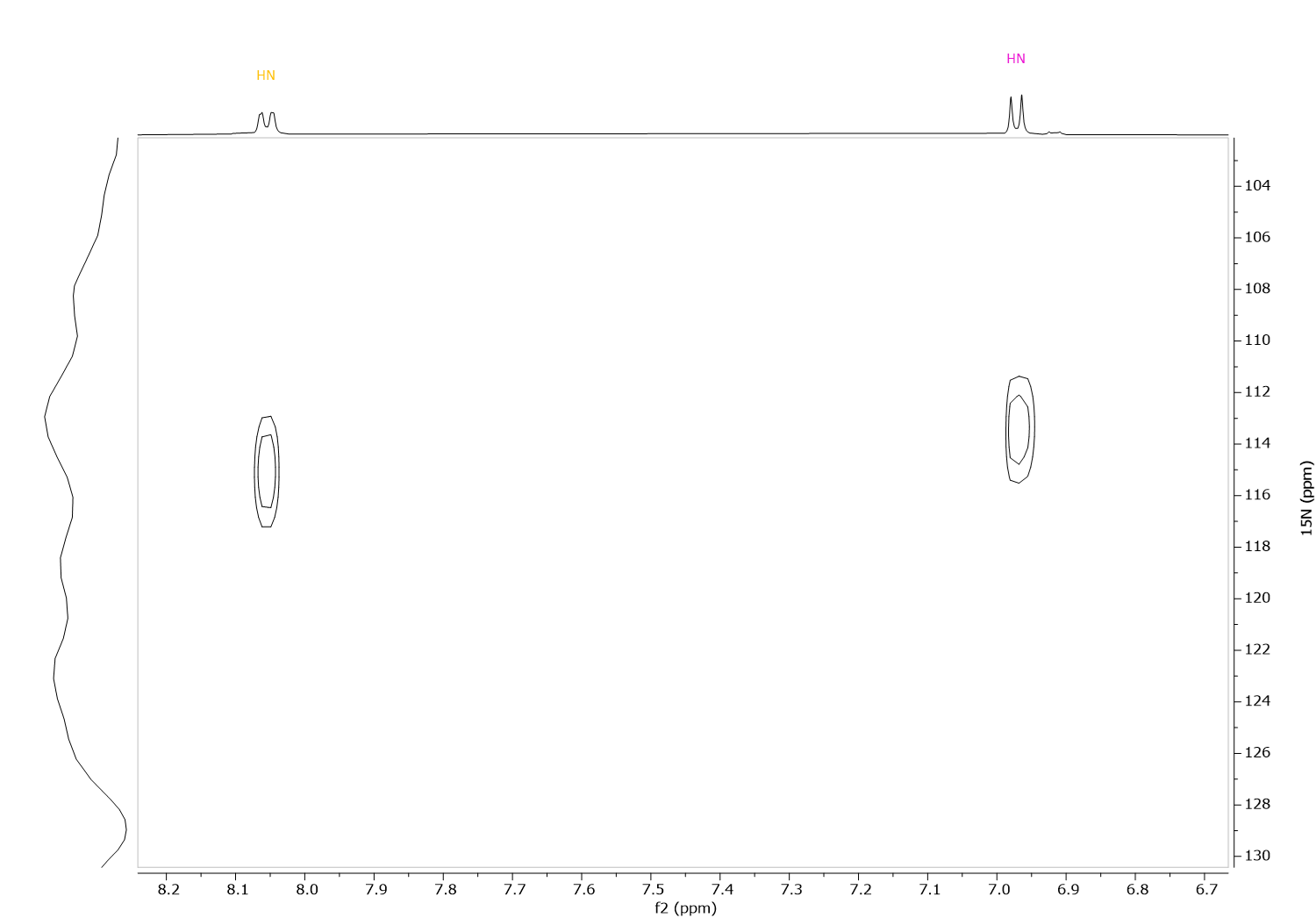


Figure S 65. ^1^H,^15^N-HSQC (Expanded) spectrum of **4**.


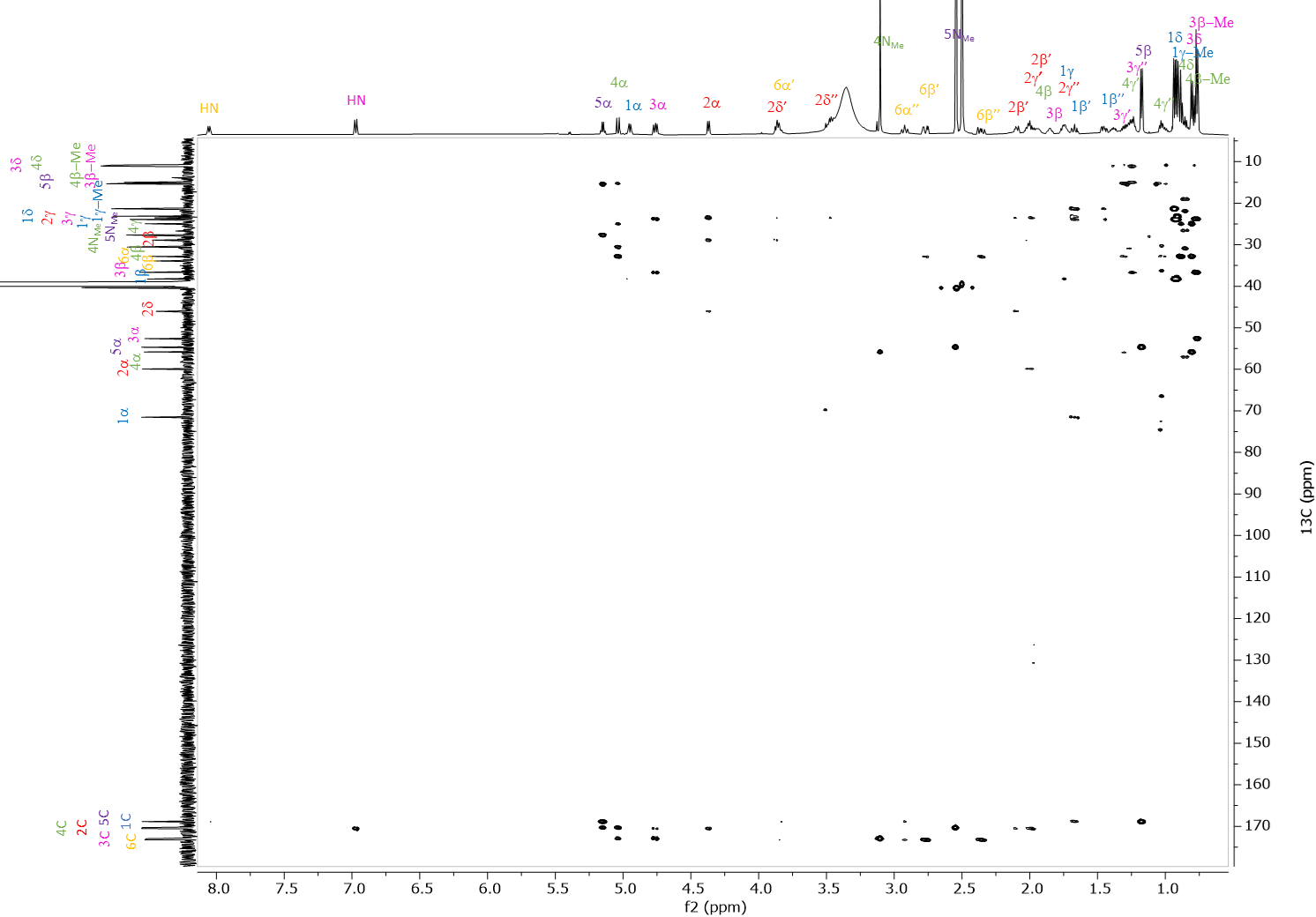


Figure S 66. HMBC spectrum of **4**.


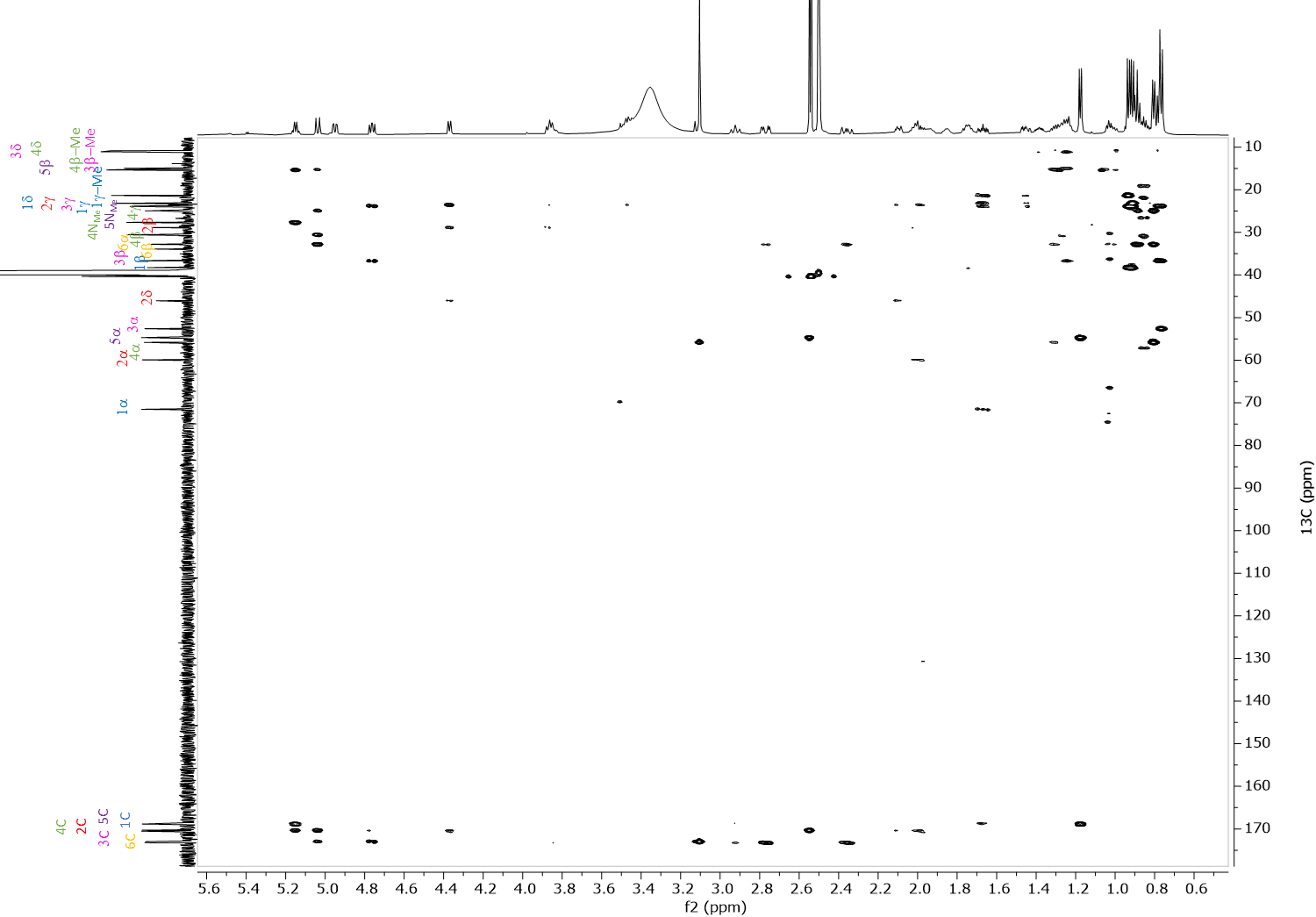


Figure S 67. HMBC (Expanded) spectrum of **4**.


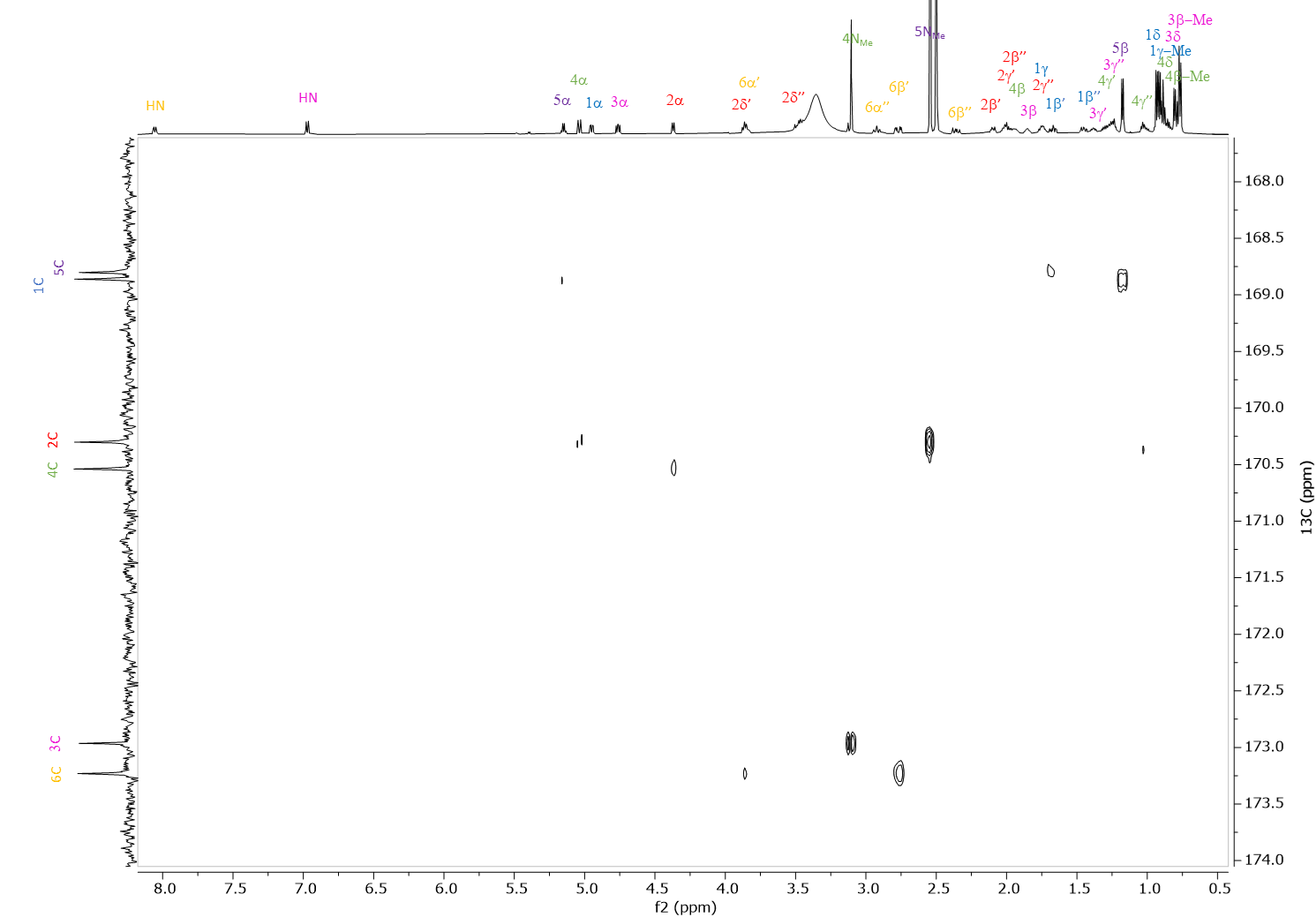


Figure S 68. Selective HMBC (8 Hz) spectrum of **4**.


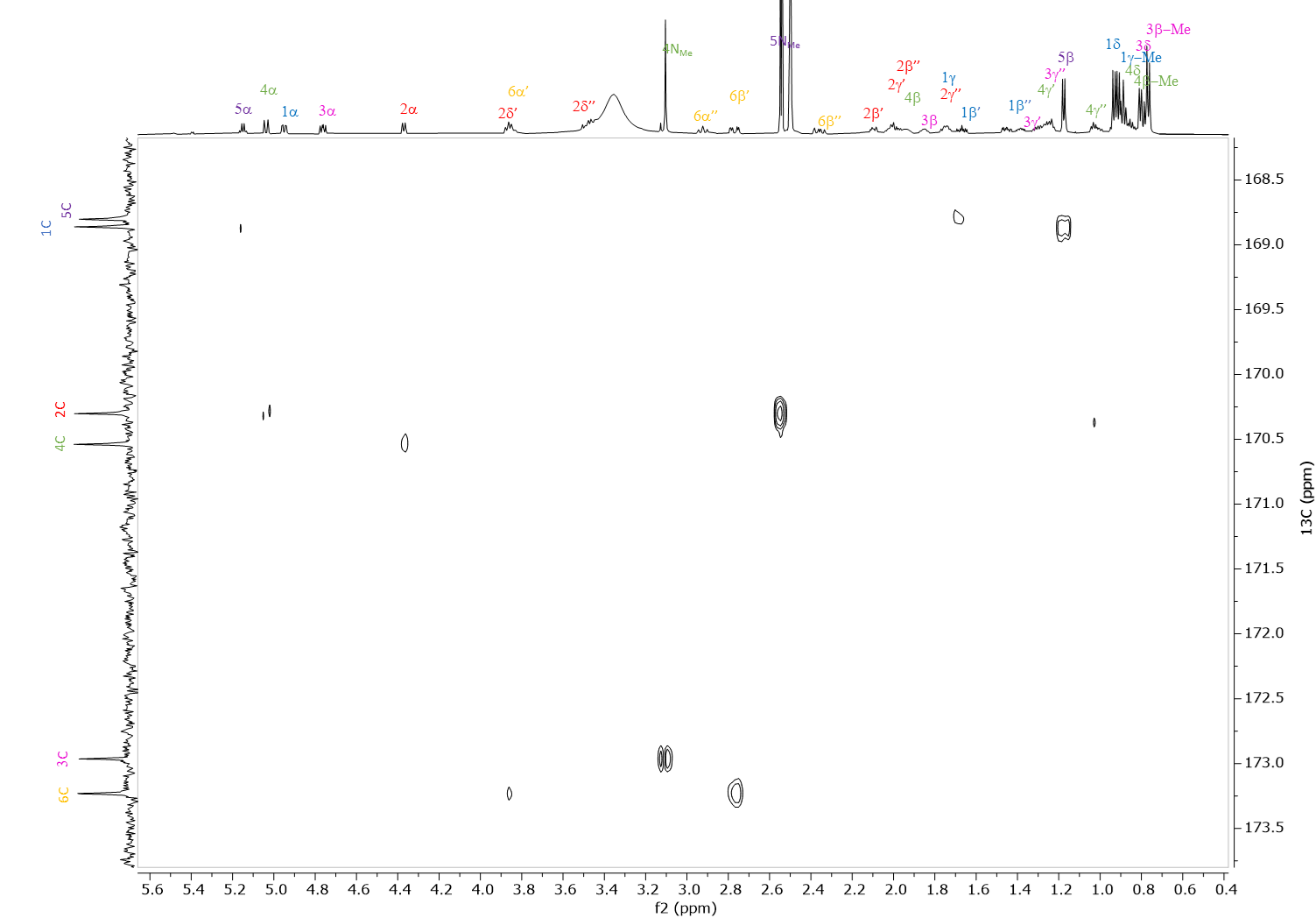


Figure S 69. Selective HMBC (Expanded) spectrum of **4**.


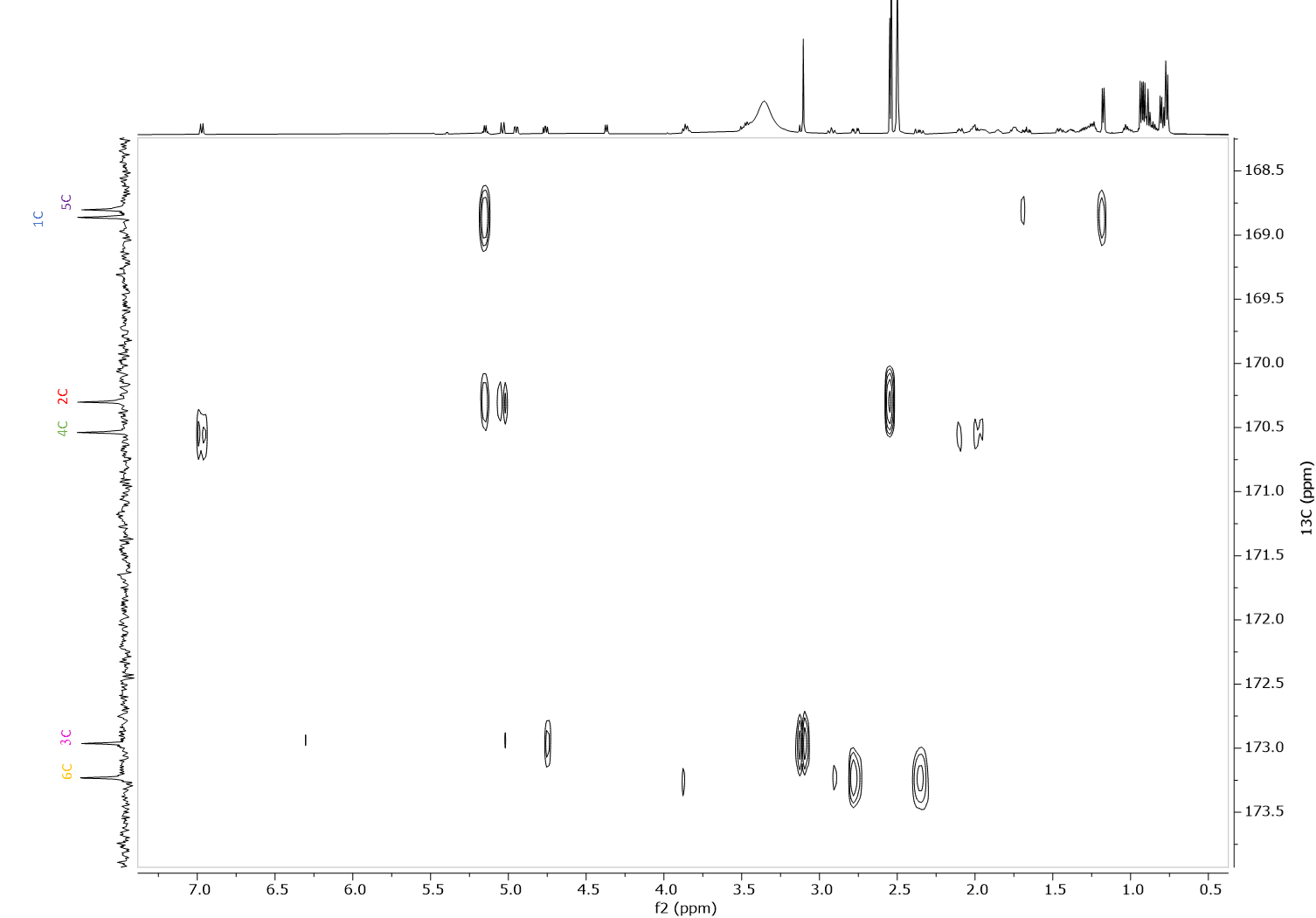


Figure S 70. Selective HMBC (8 Hz) spectrum of **4**.


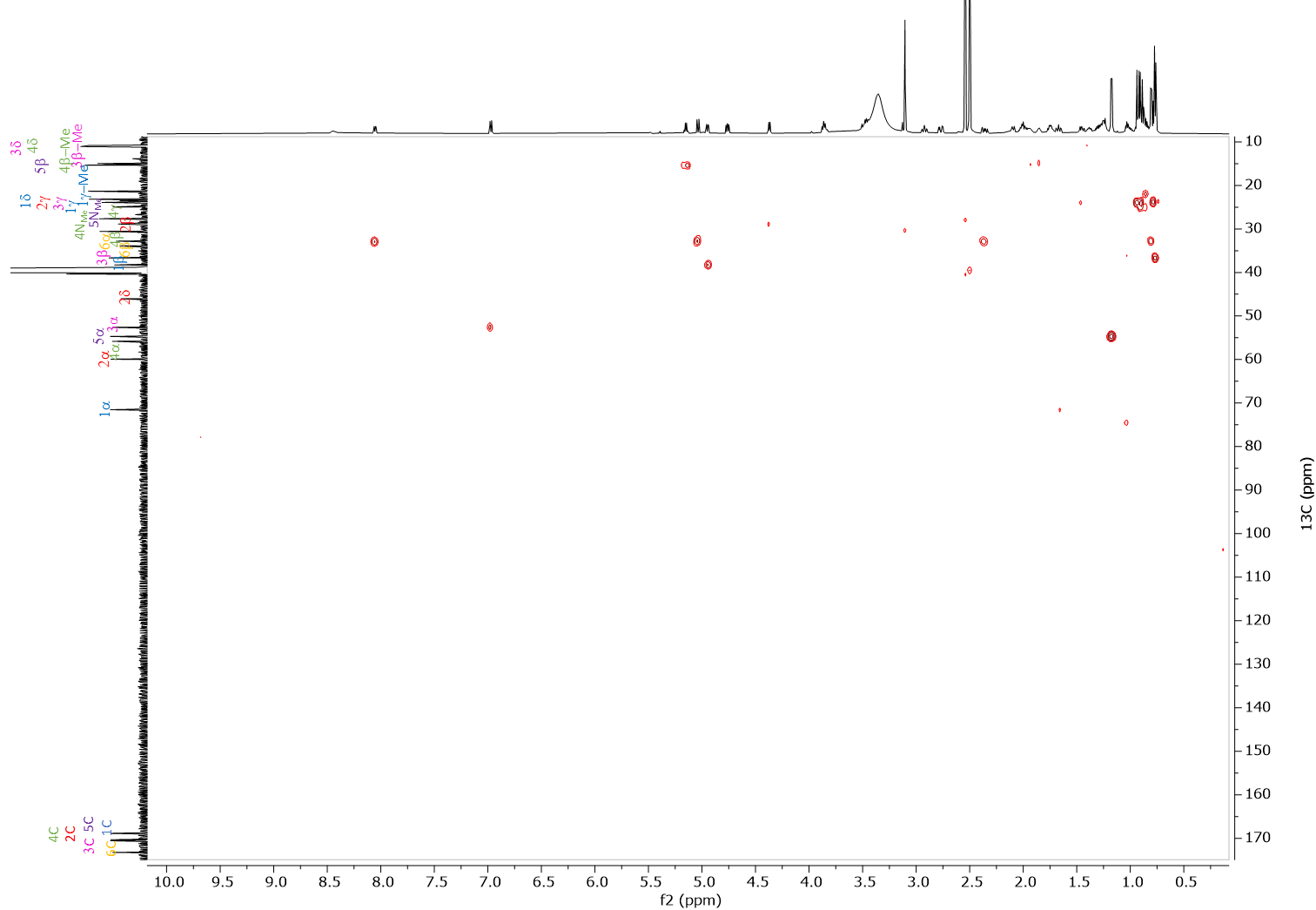


Figure S 71. H2BC spectrum of **4**.


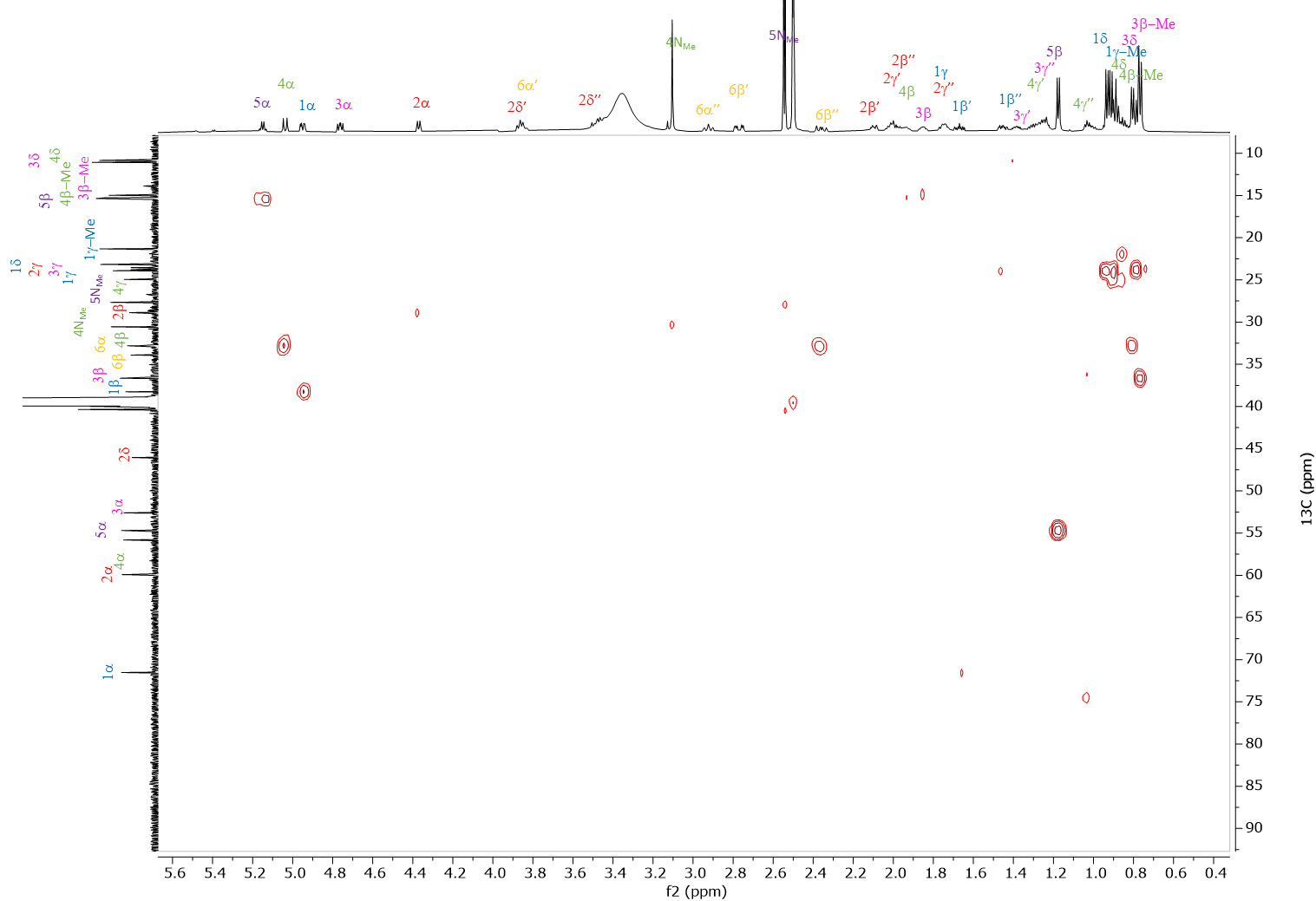


Figure S 72. H2BC (Expanded) spectrum of **4**.


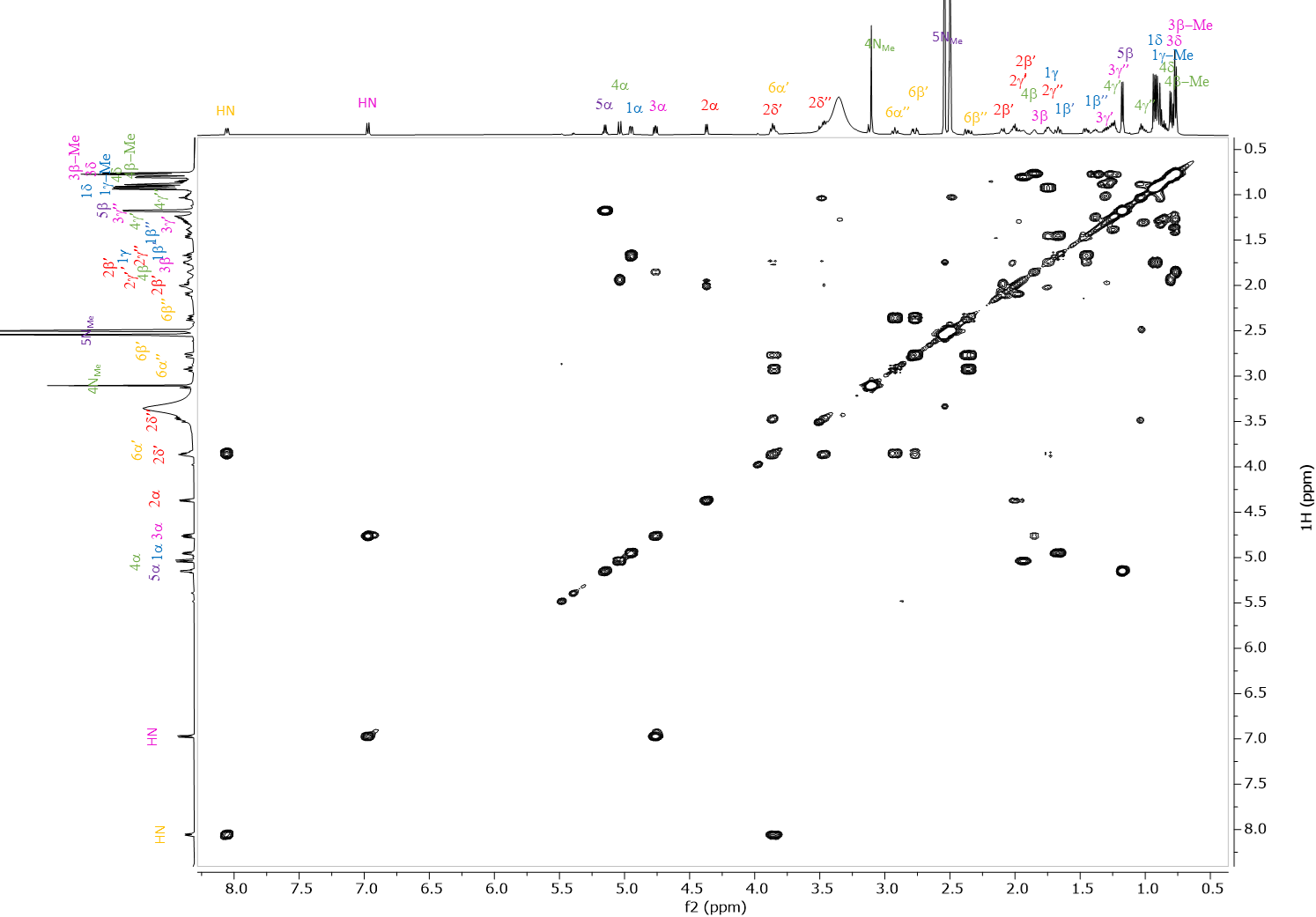


Figure S 73. COSY spectrum of **4**.


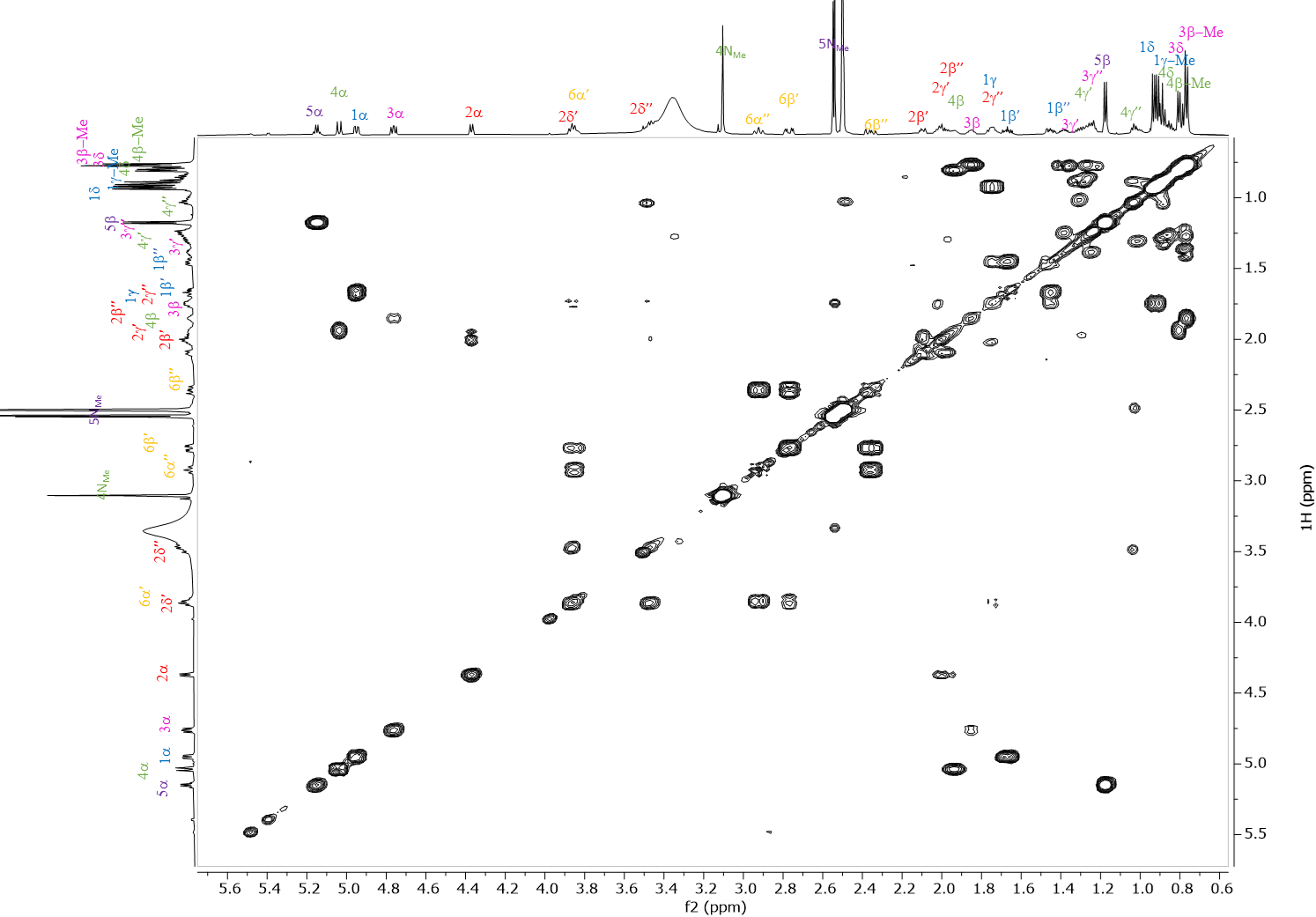


Figure S 74. COSY (Expanded) spectrum of **4**.


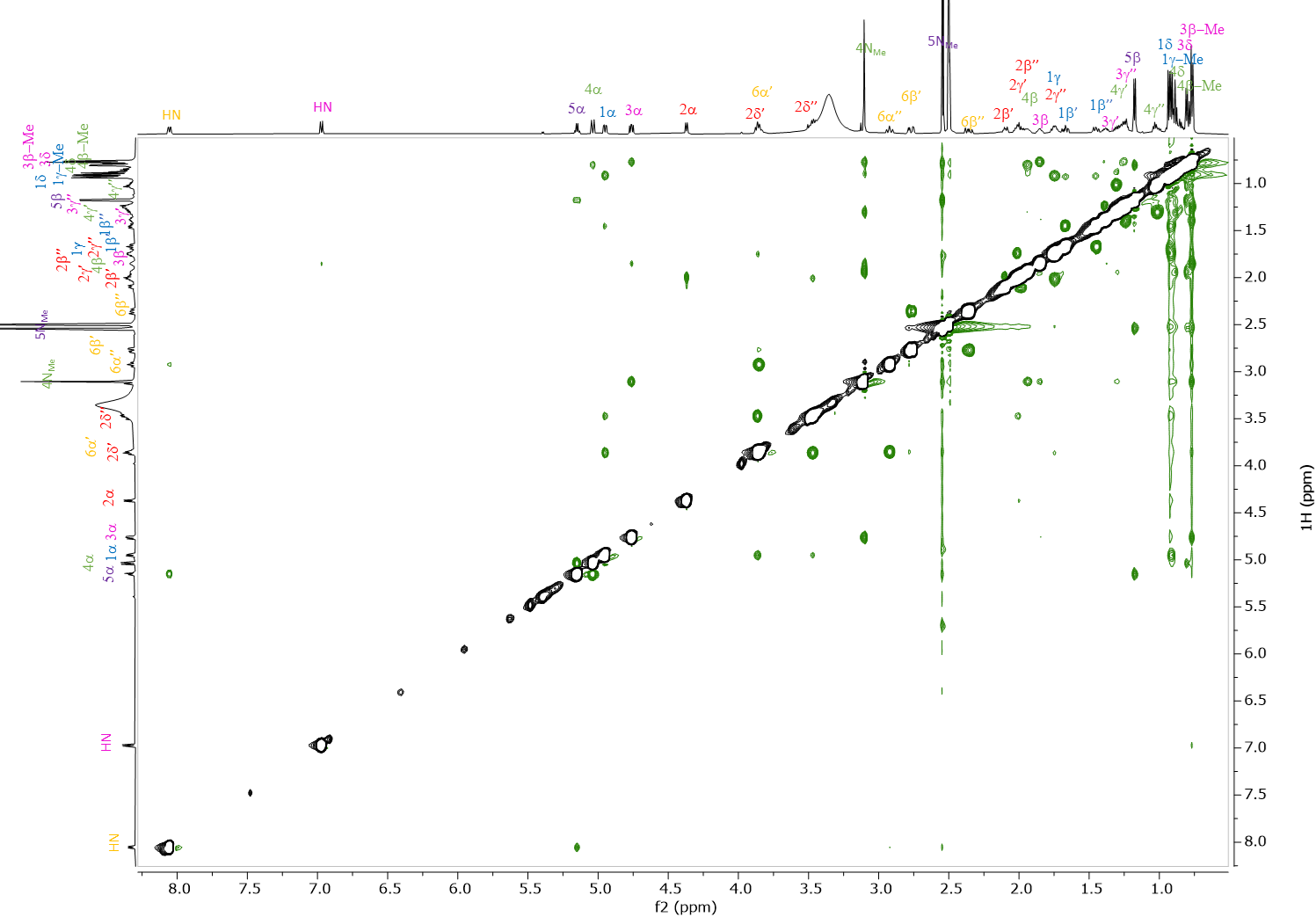


Figure S 75. ROESY spectrum of **4**.


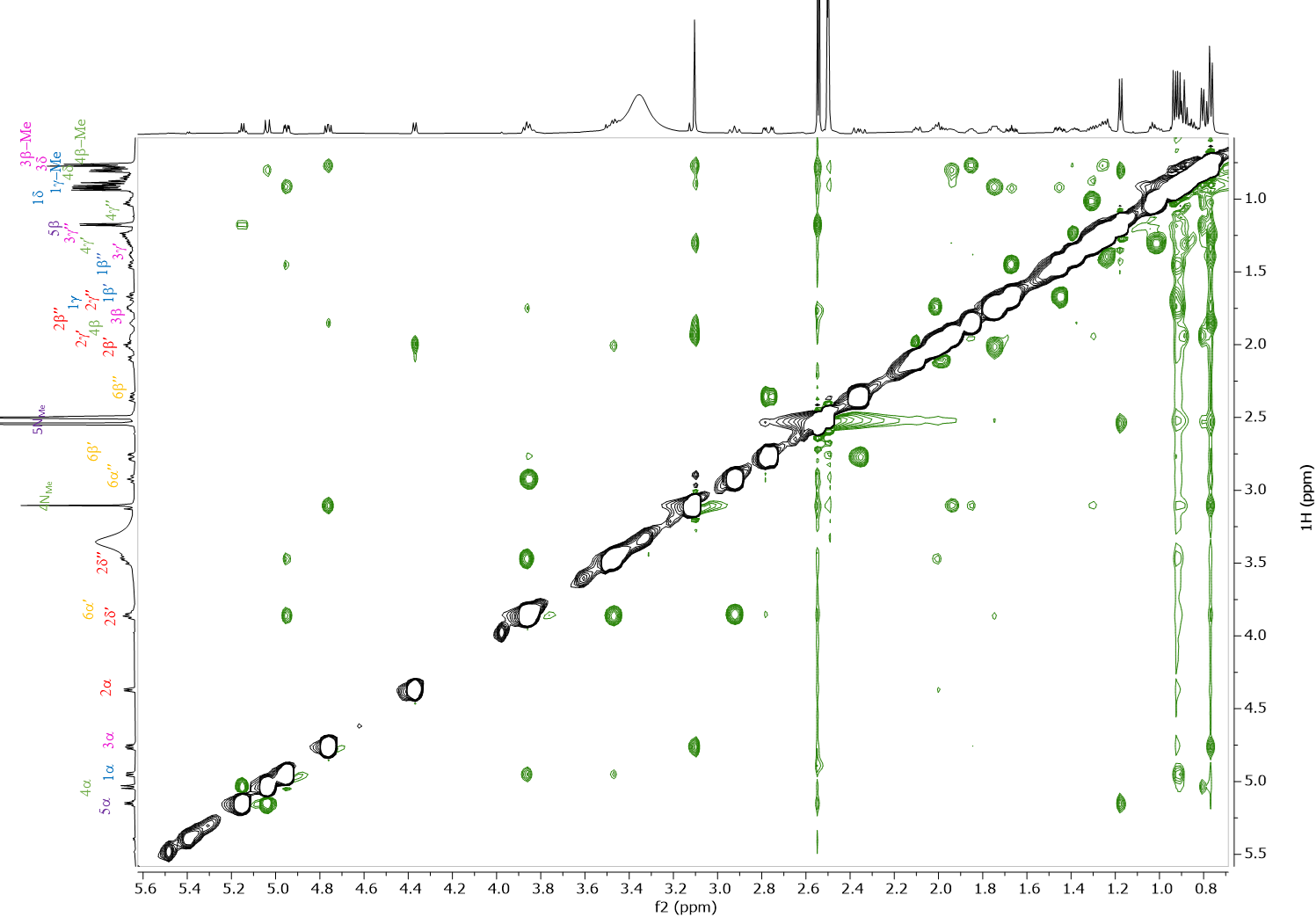


Figure S 76. ROESY spectrum of **4**.

**Compound 5**

**
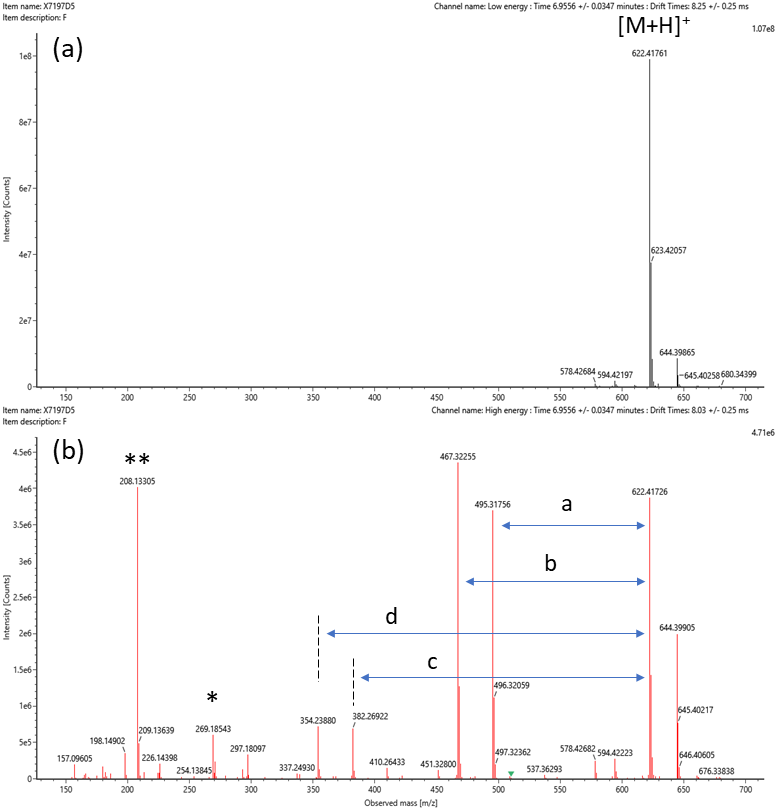
**

**
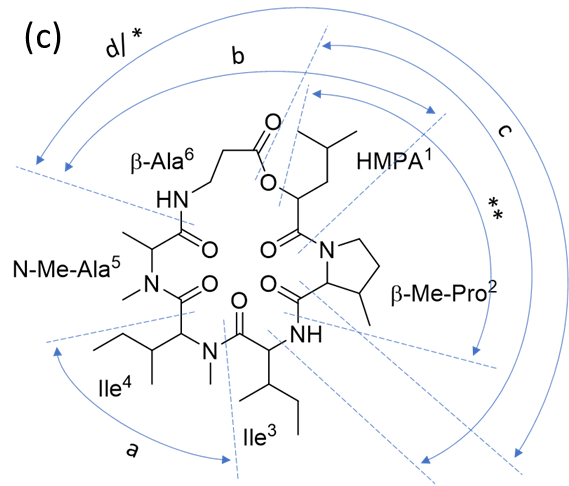
**

Figure S 77. HRESIMS of **5** at (a) low collision energy and (b) high collision energy in ESI+, HDMS^E^ mode. Fragmentation of **5**.


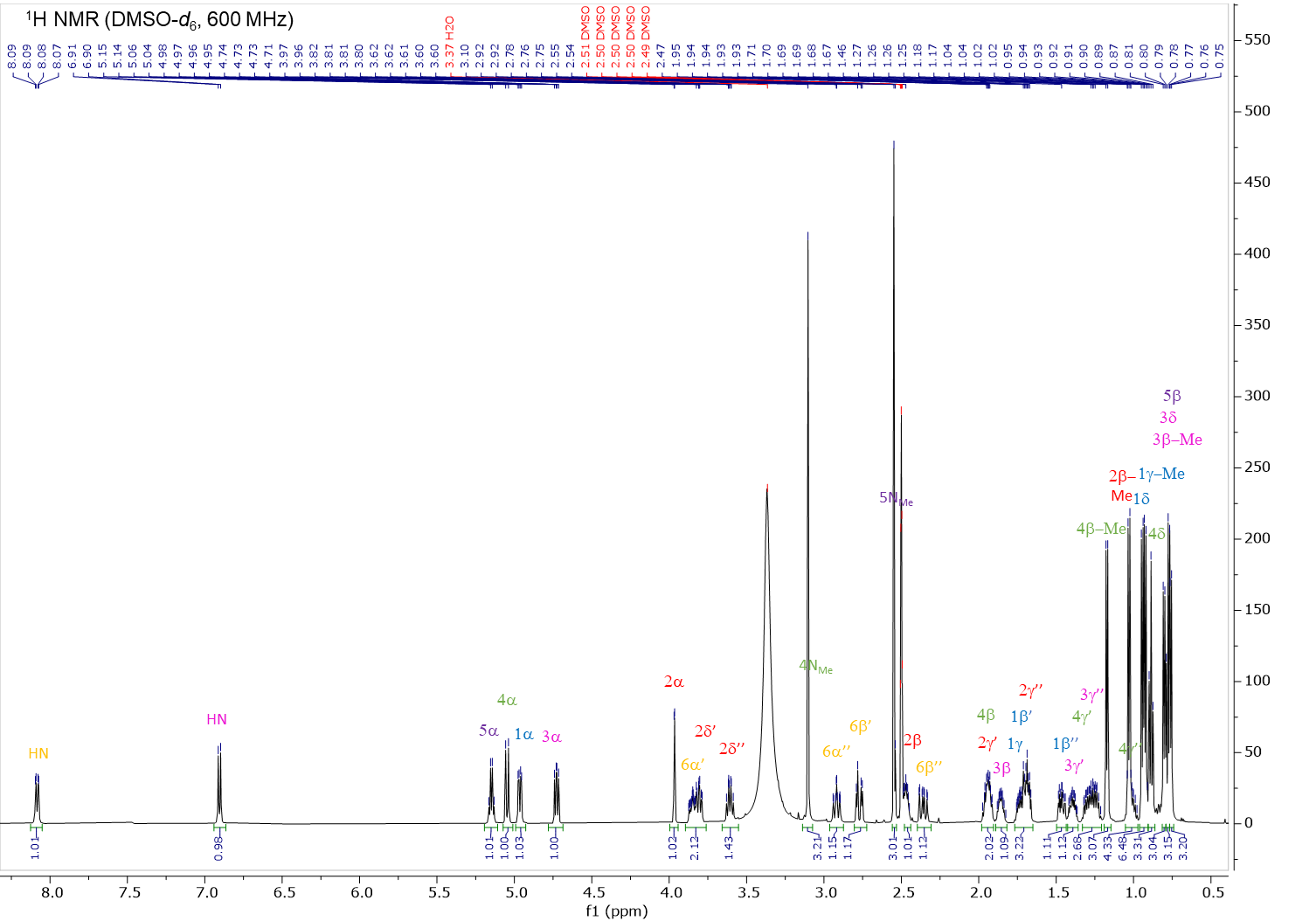


Figure S 78. ^1^H NMR spectrum of **5**.


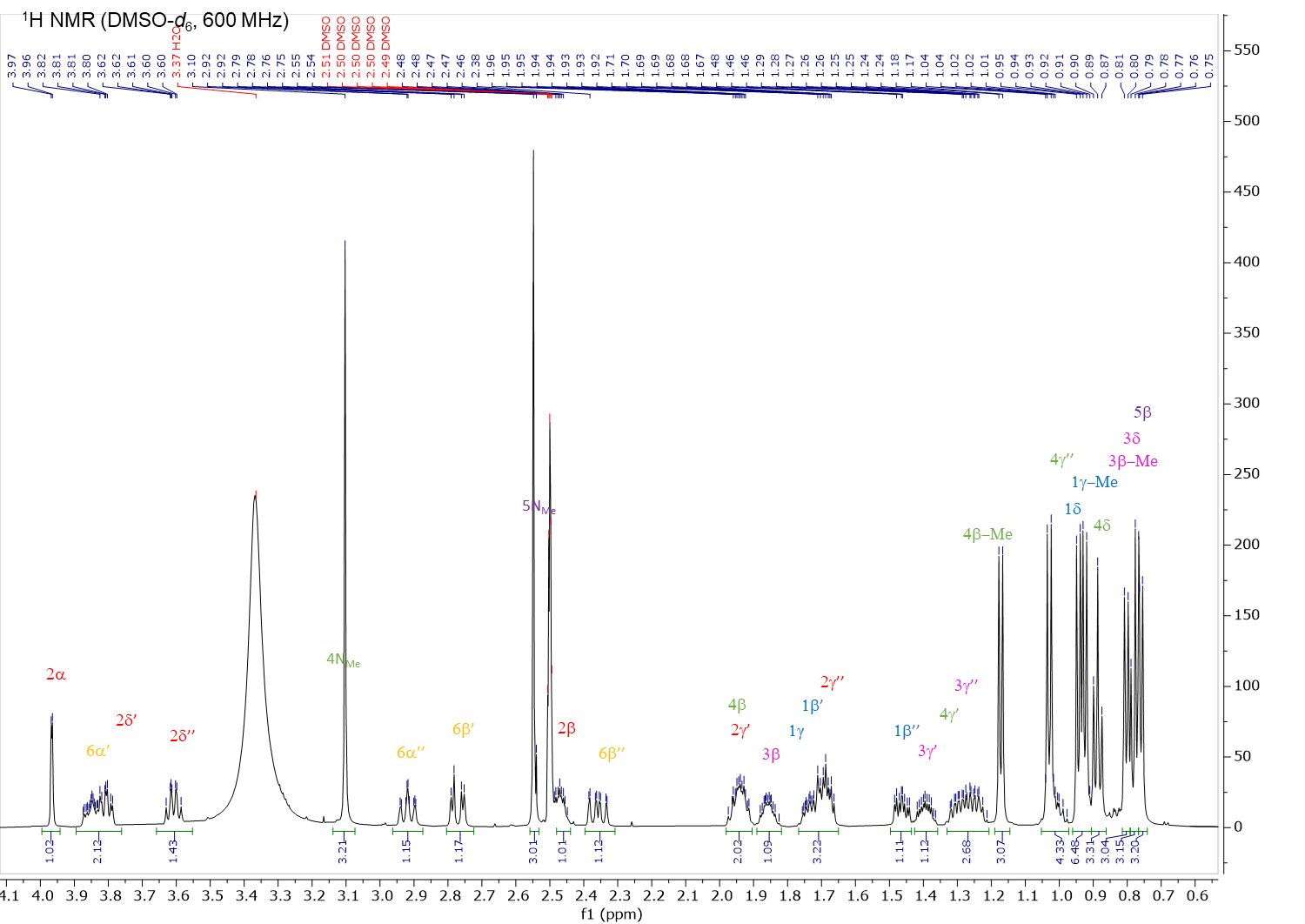


Figure S 79. ^1^H NMR (Expanded) spectrum of **5**.


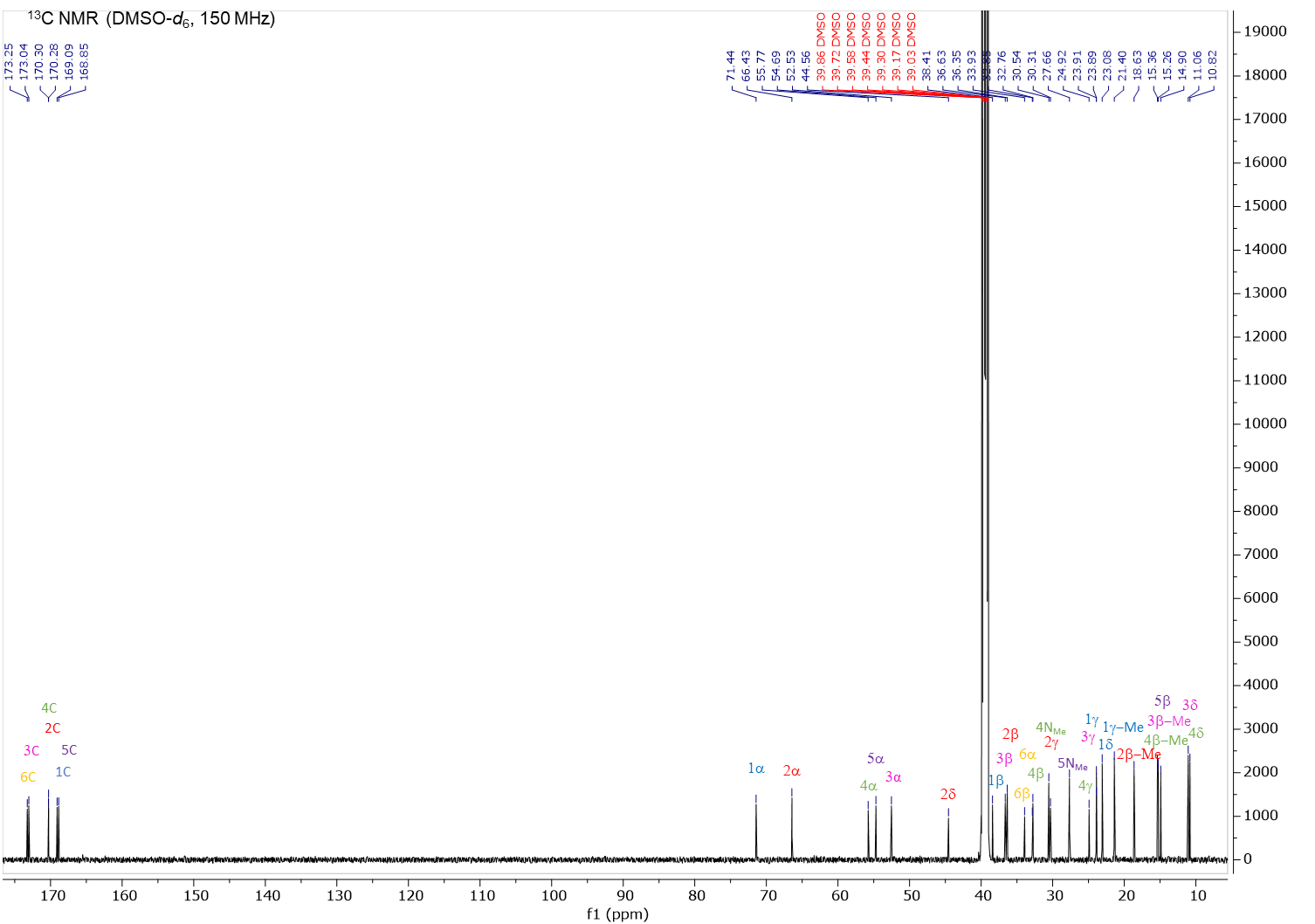


Figure S 80. ^13^C NMR spectrum of **5**.


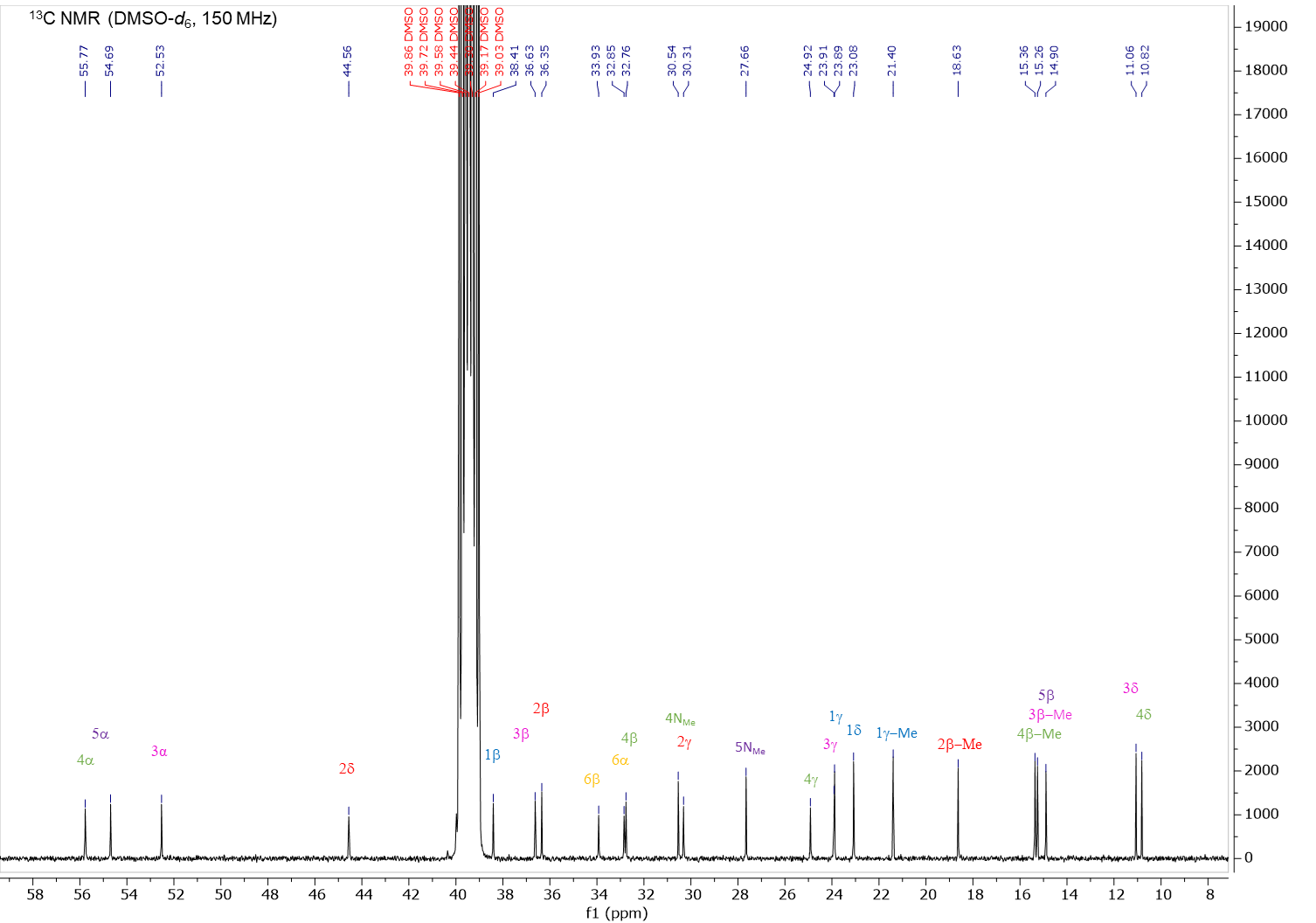


Figure S 81. ^13^C NMR (Expanded) spectrum of **5**.


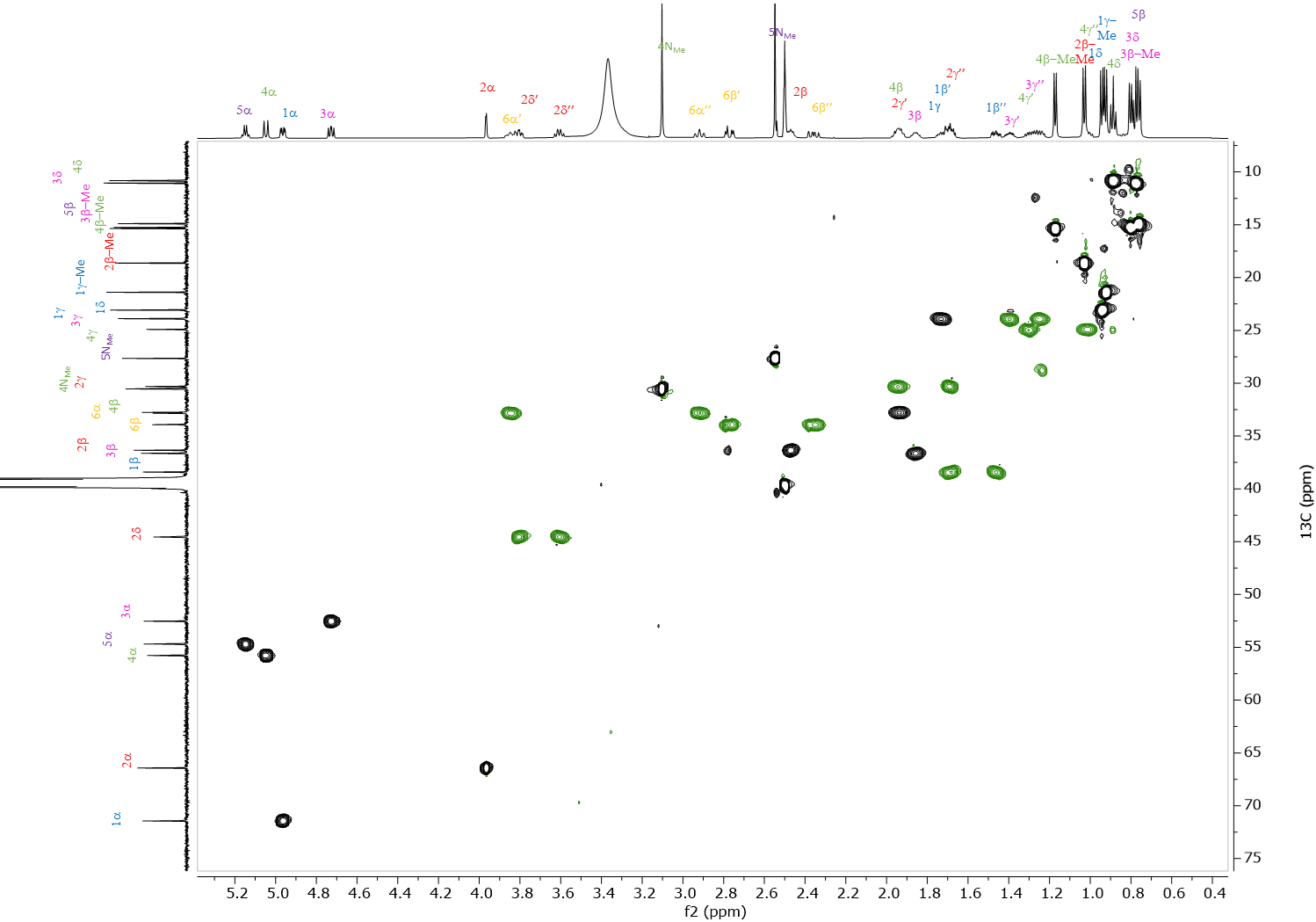


Figure S 82. HSQC spectrum of **5**.


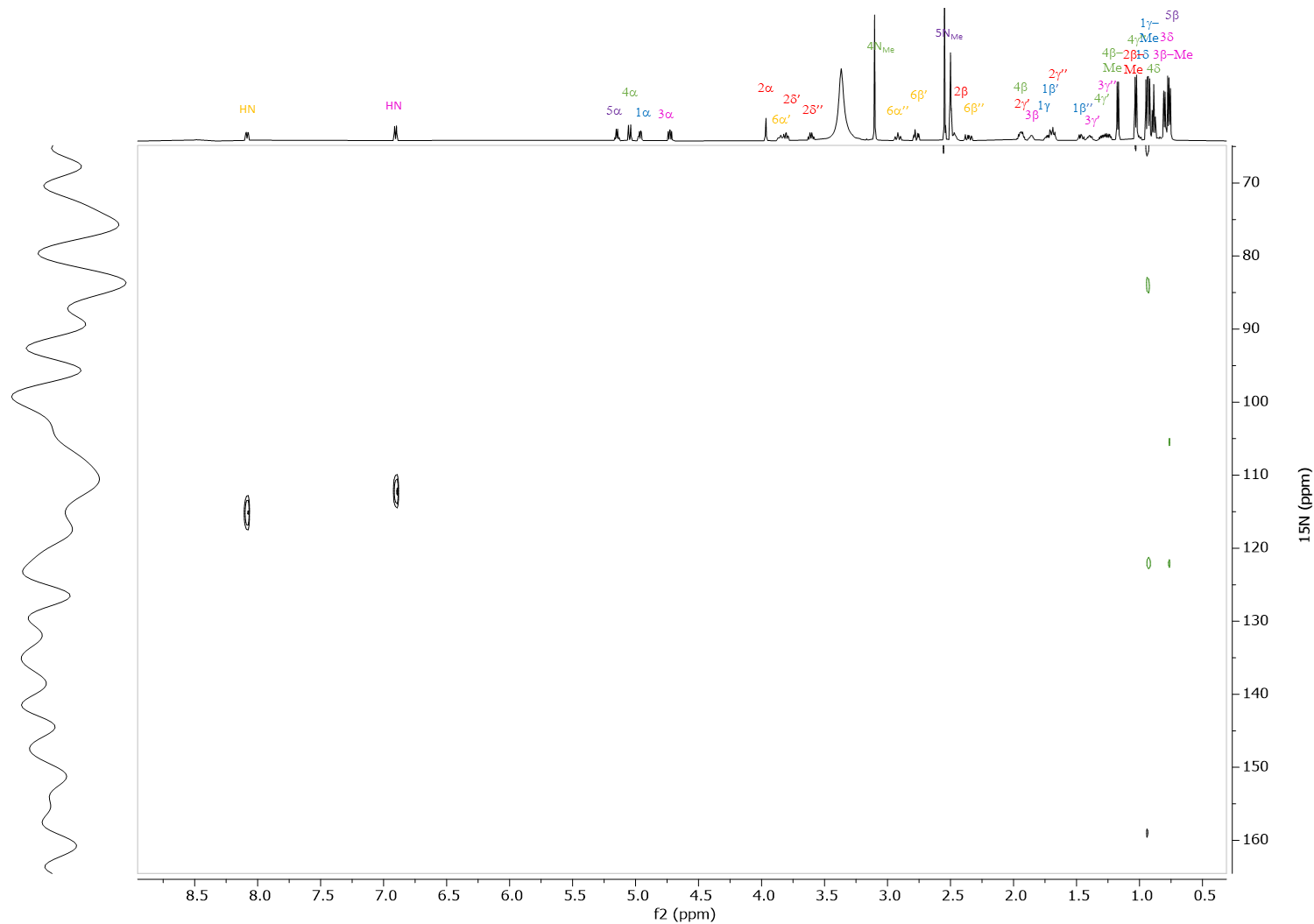


Figure S 83. ^1^H,^15^N-HSQC spectrum of **5**.


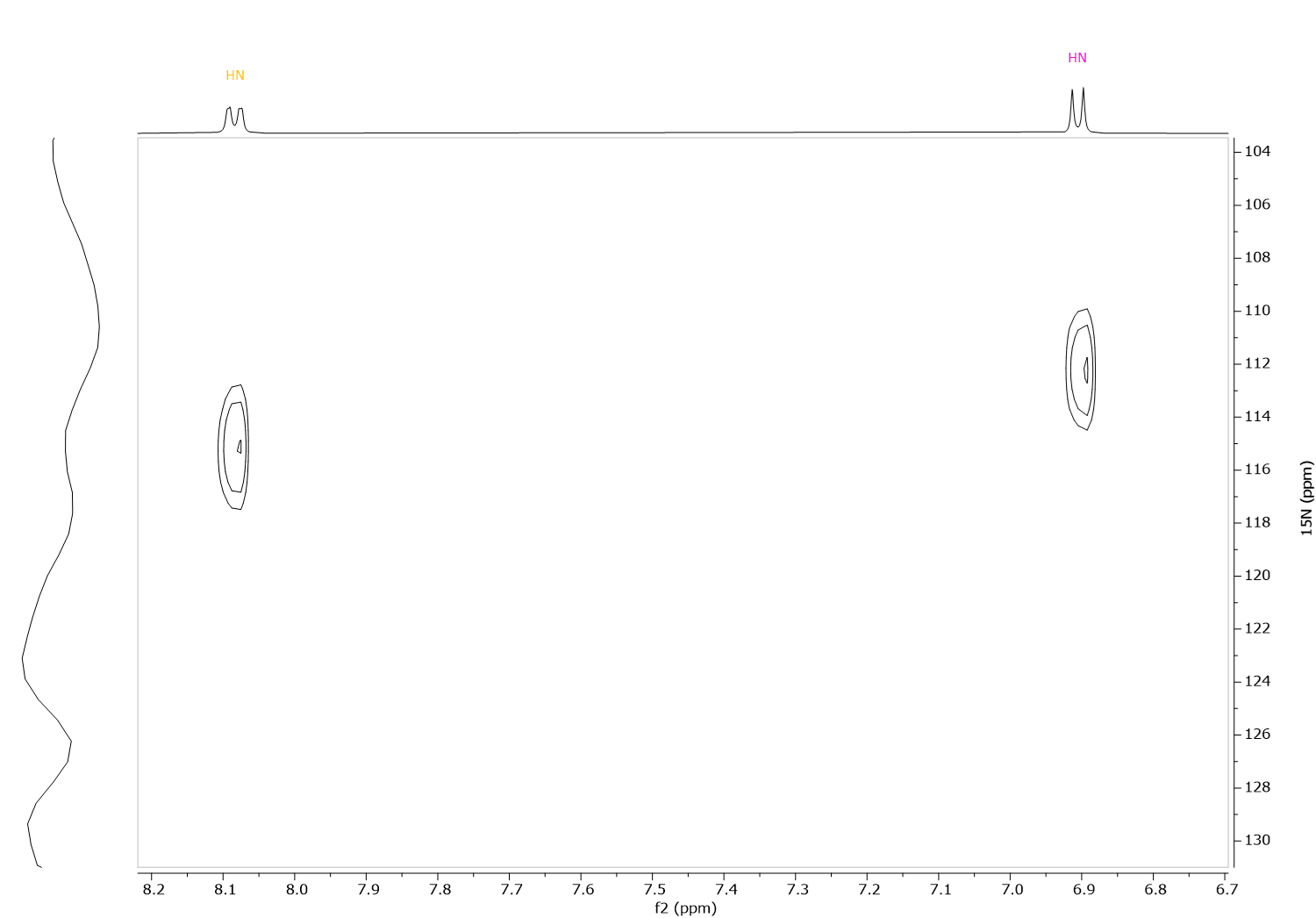


Figure S 84. ^1^H,^15^N-HSQC (Expanded) spectrum of **5**.


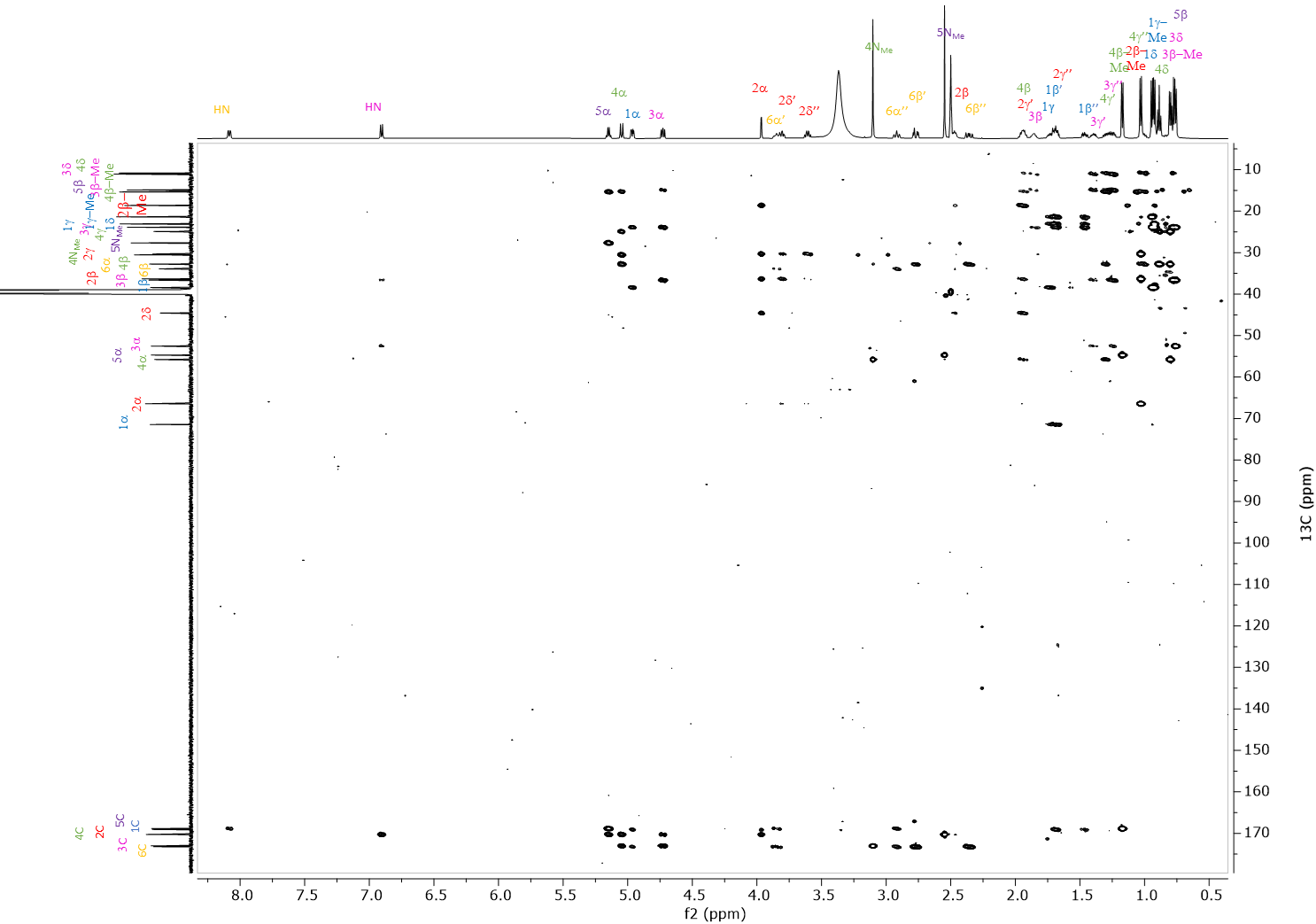


Figure S 85. HMBC spectrum of **5**.


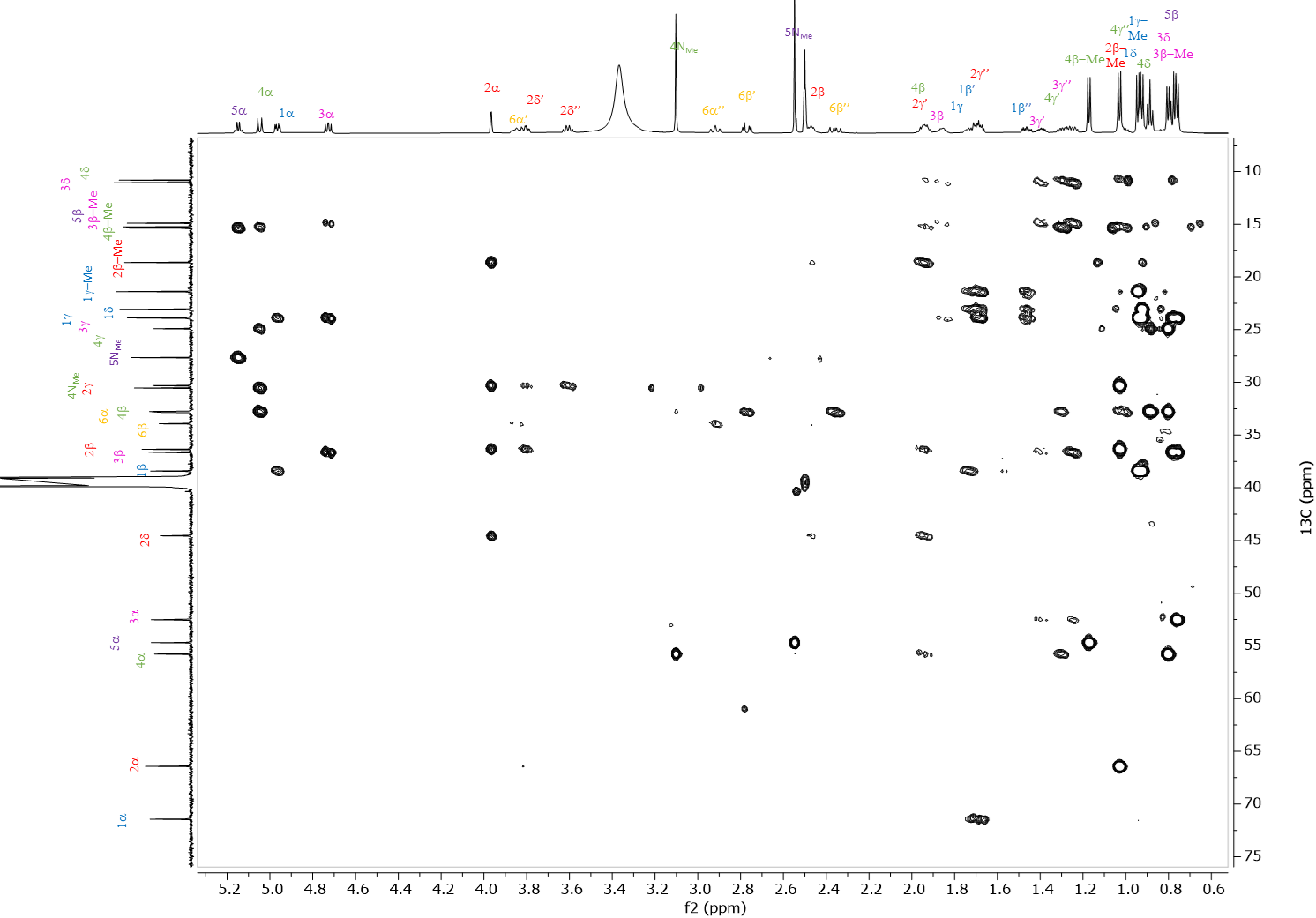


Figure S 86. HMBC (Expanded1) spectrum of **5**.


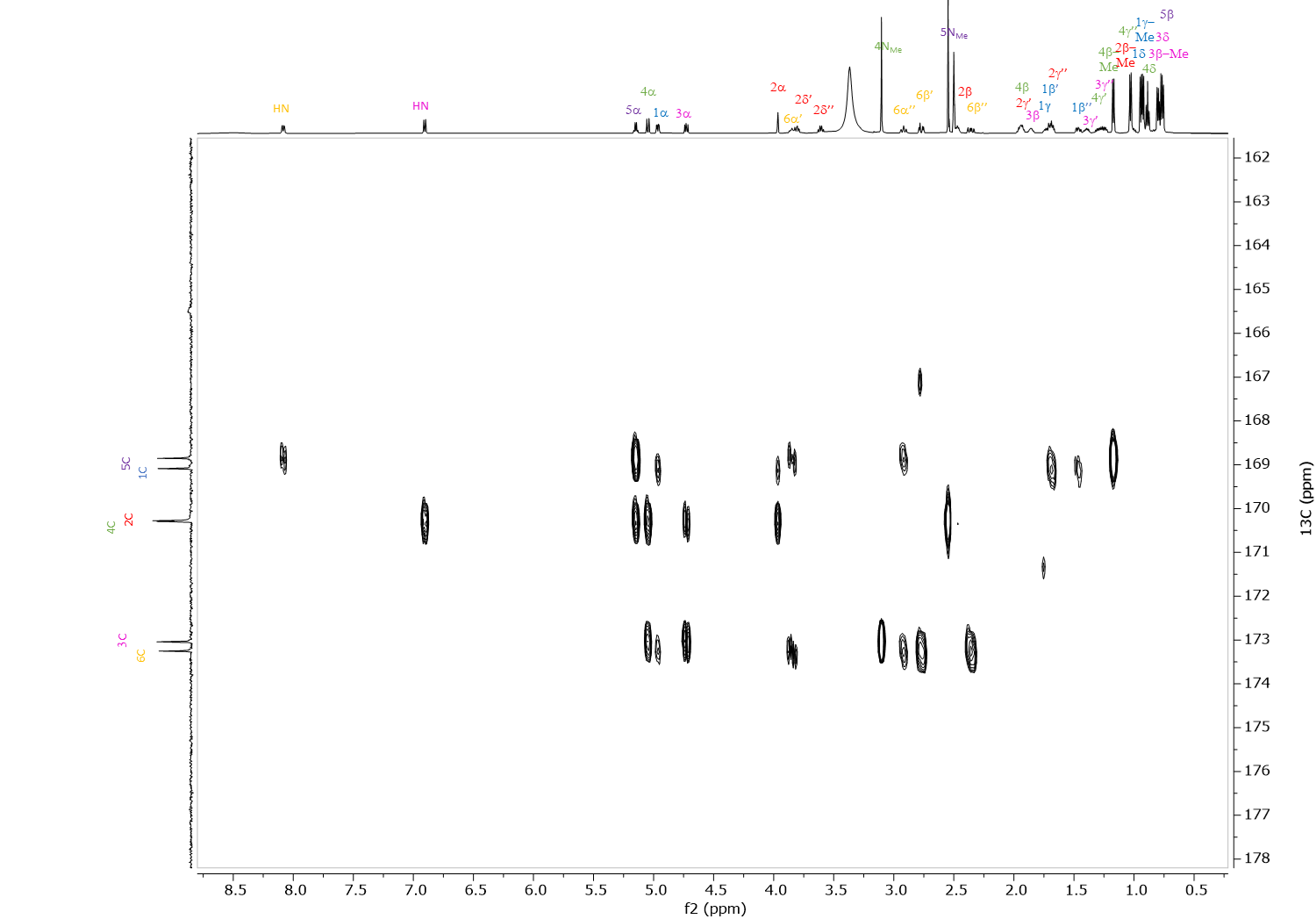


Figure S 87. HMBC (Expanded2) spectrum of **5**.


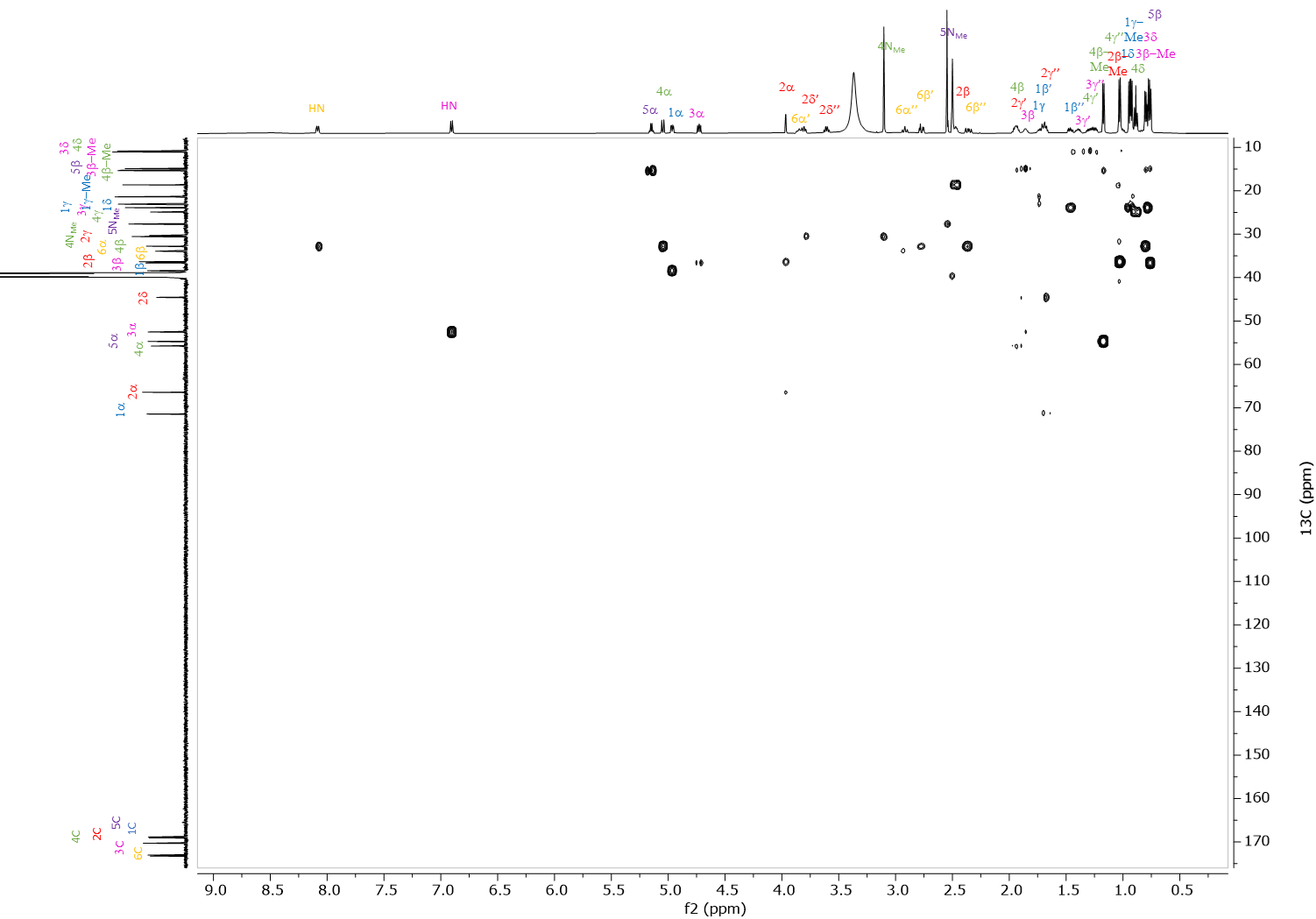


Figure S 88. H2BC spectrum of **5**.


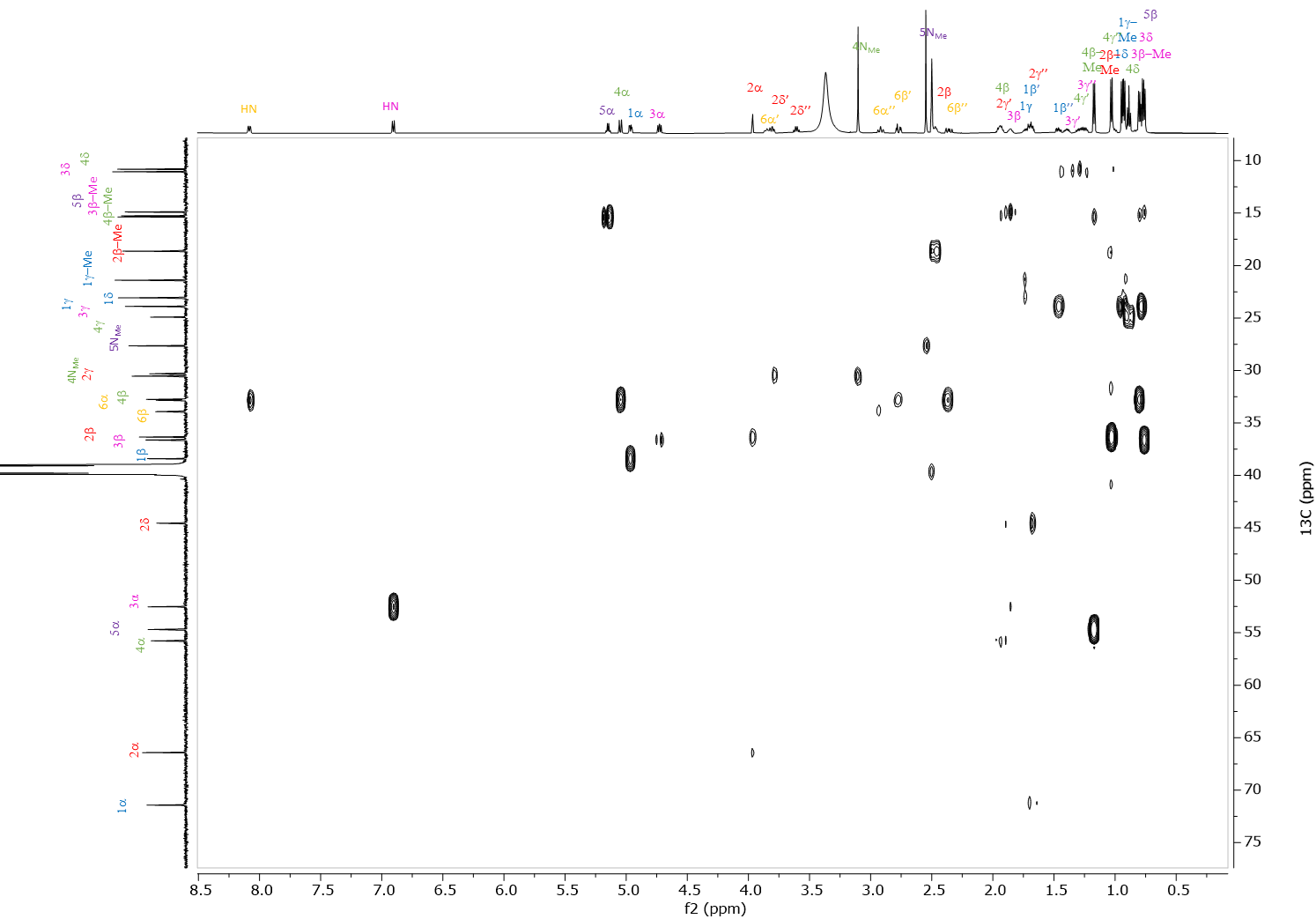


Figure S 89. H2BC (Expanded) spectrum of **5**.


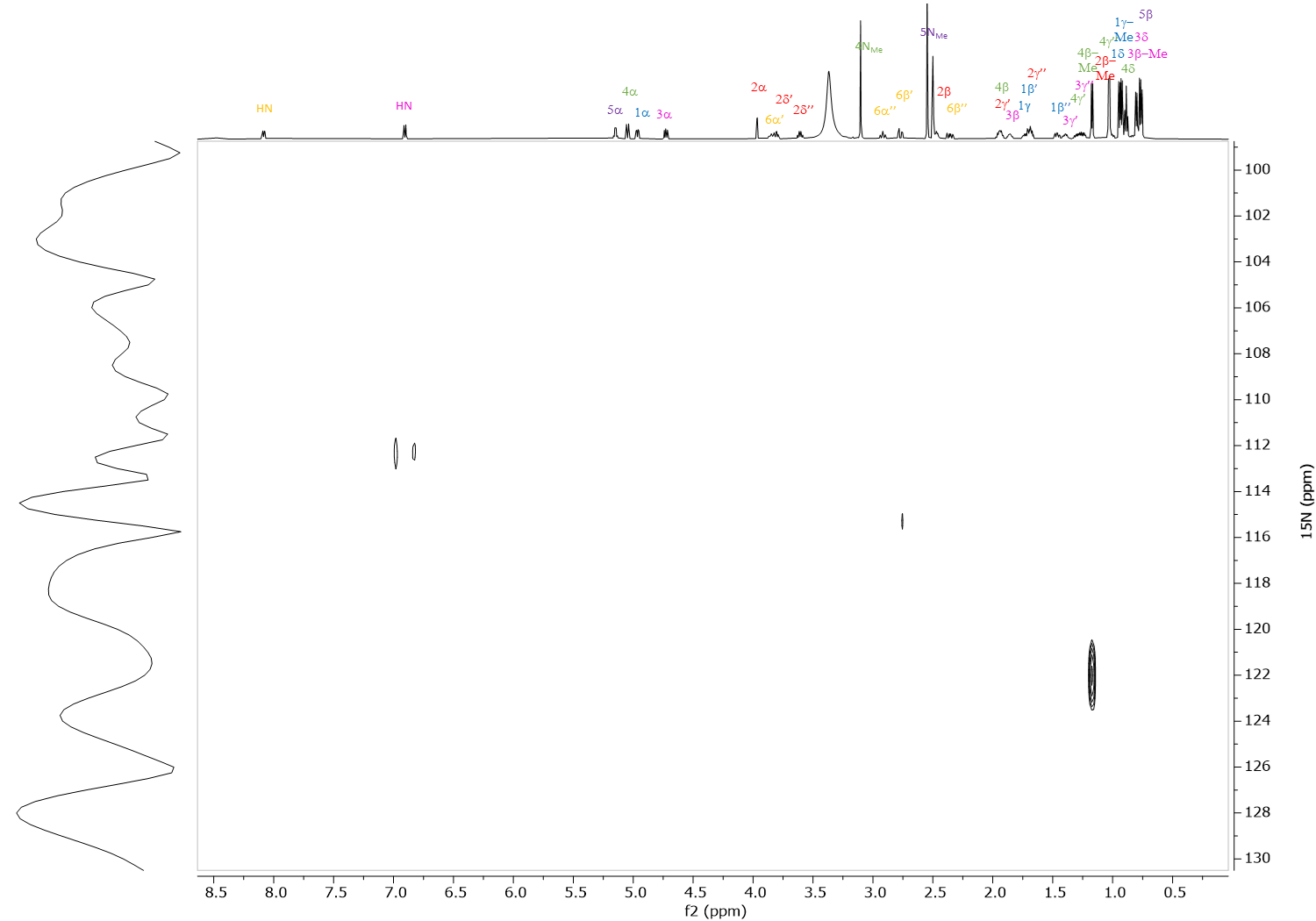


Figure S 90. ^1^H,^15^N-HMBC spectrum of **5**.


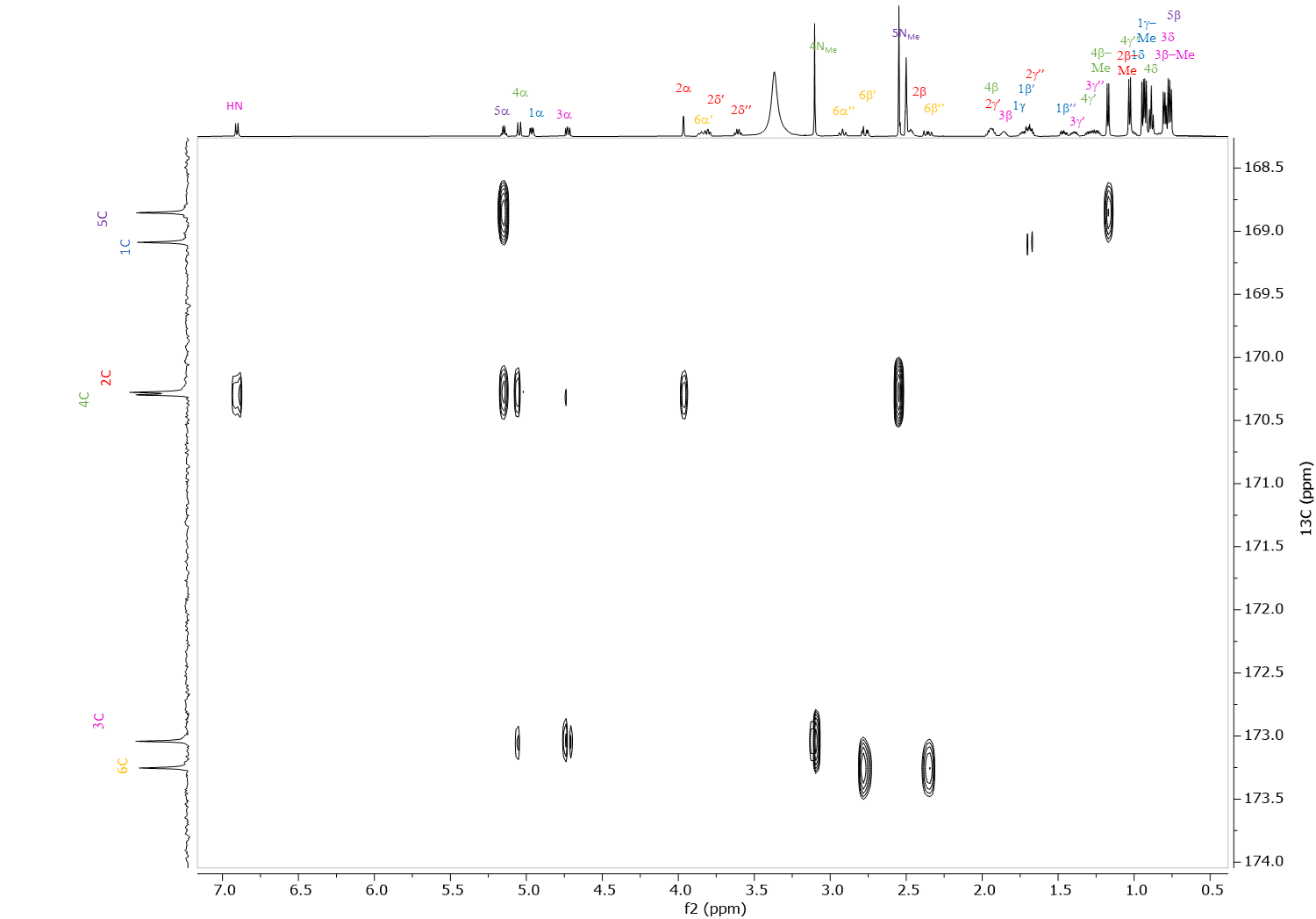


Figure S 91. Selective HMBC (8 Hz) spectrum of **5**.


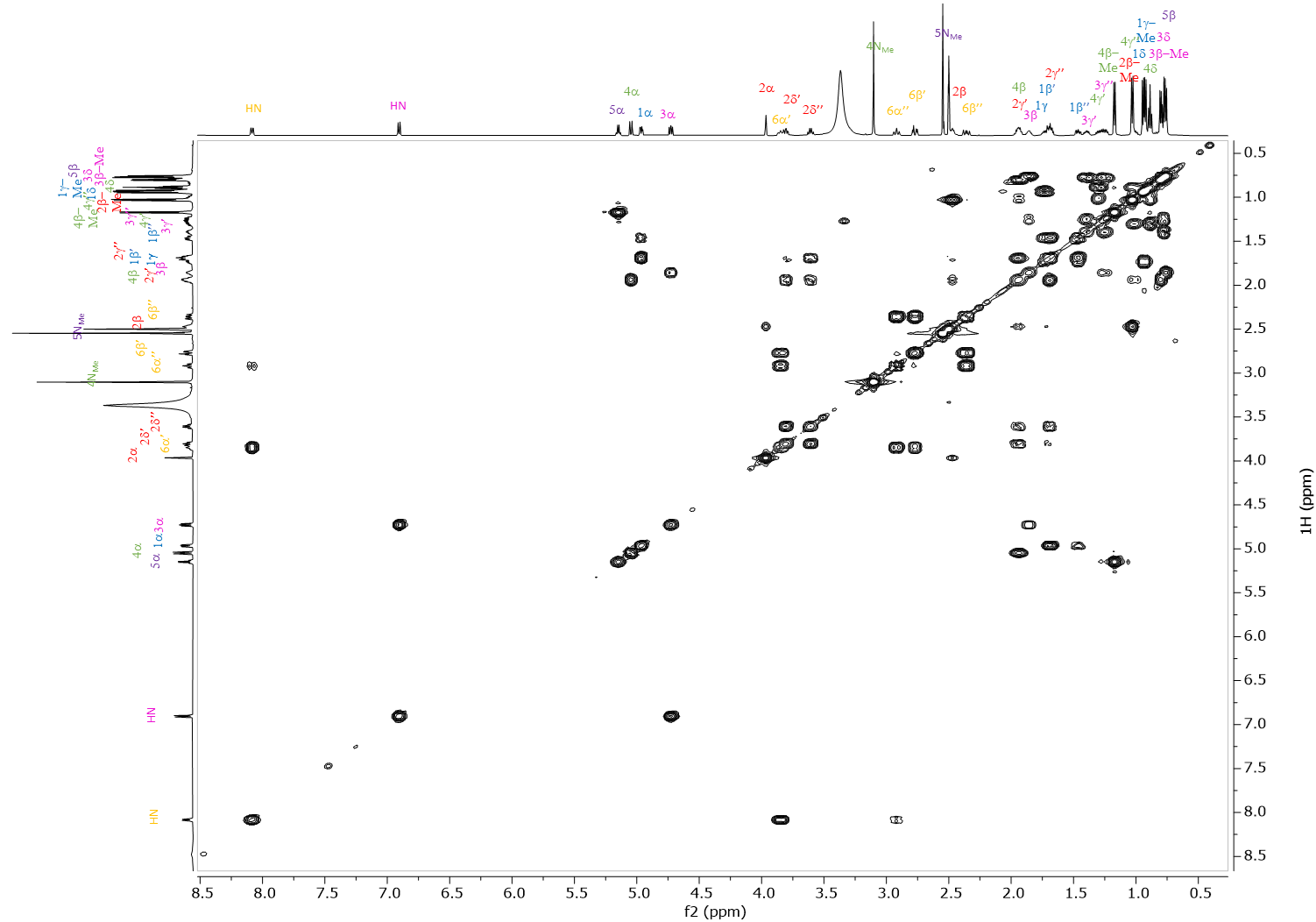


Figure S 92. COSY spectrum of **5**.


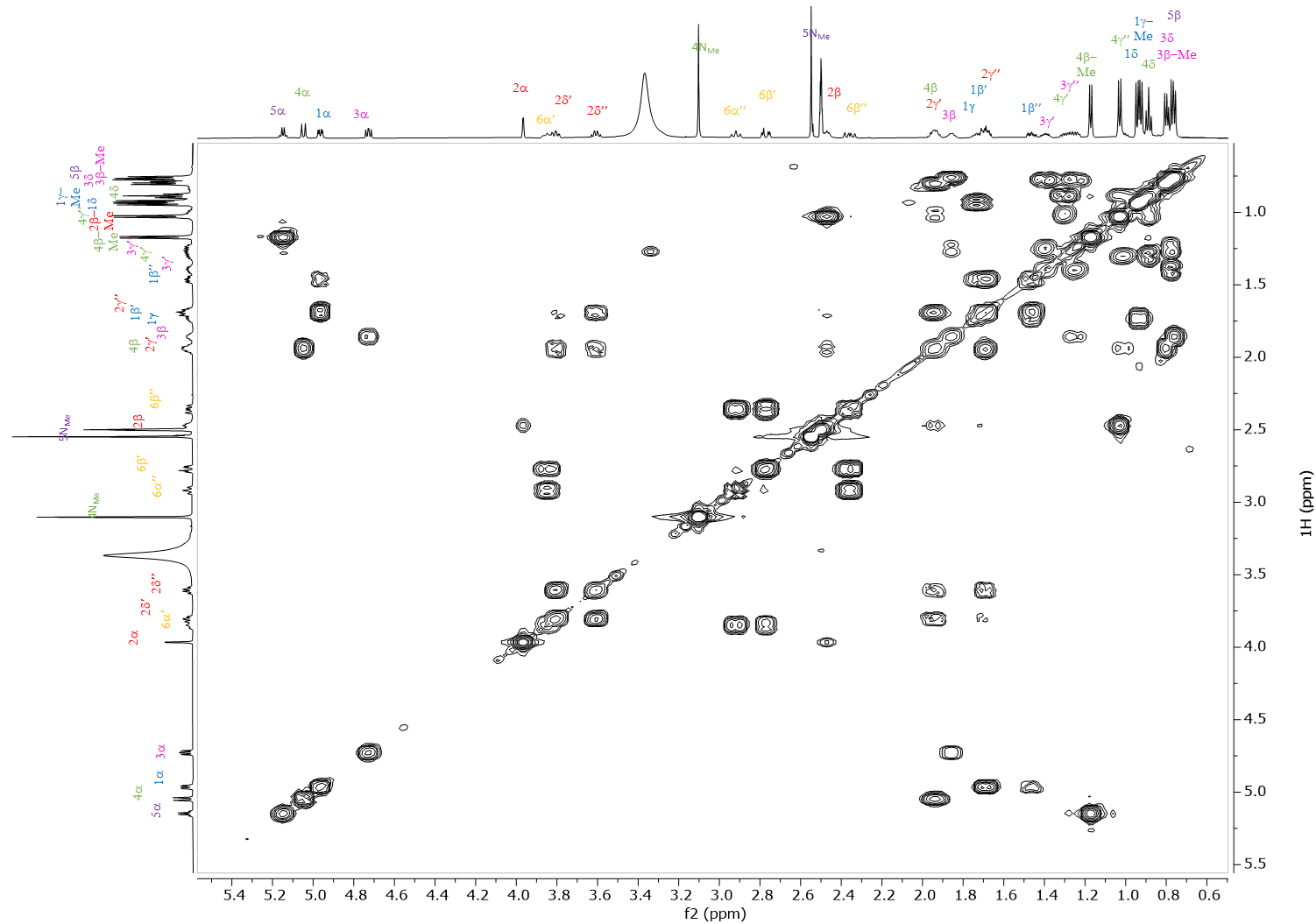


Figure S 93. COSY (Expanded) spectrum of **5**.


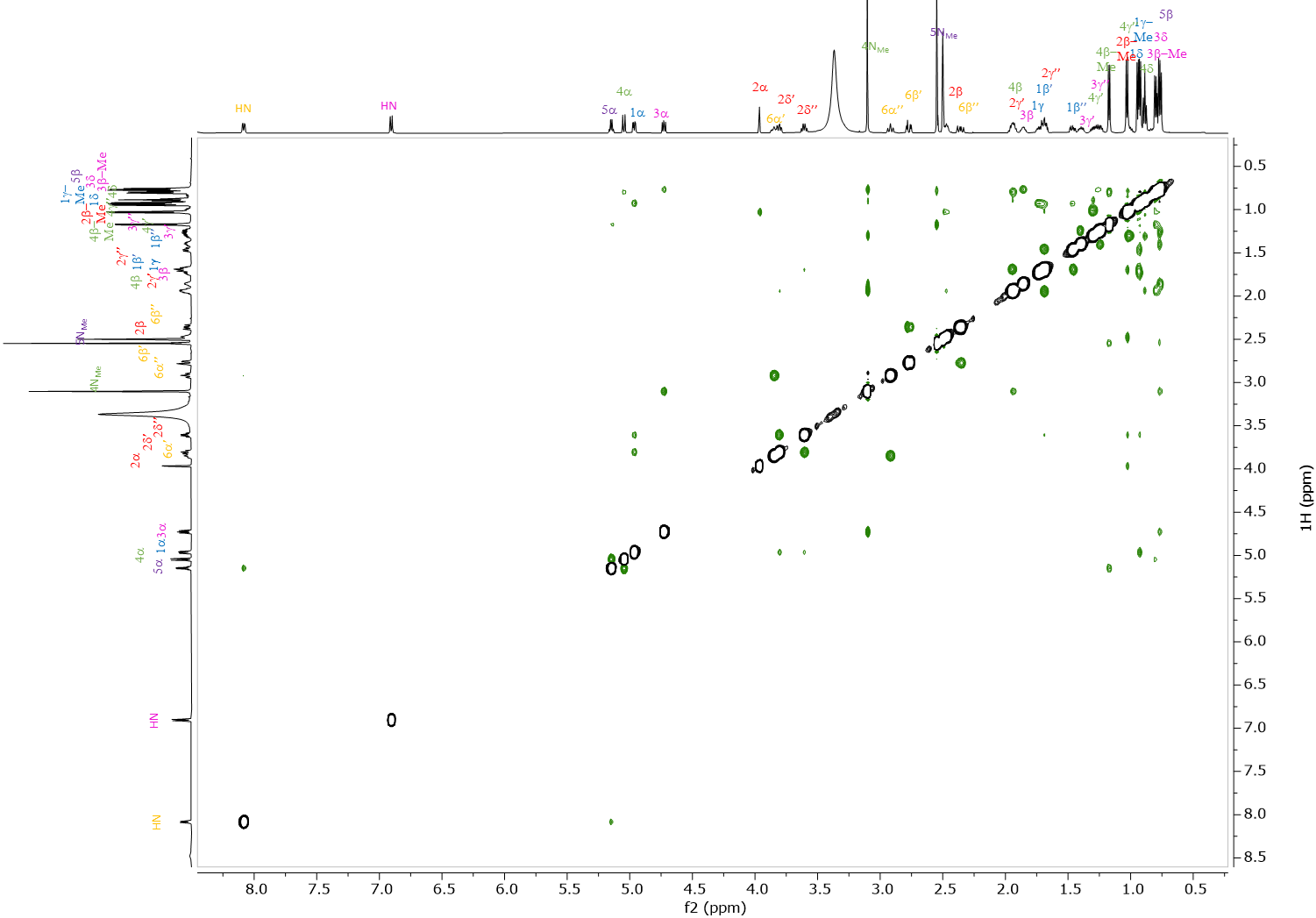


Figure S 94. ROESY spectrum of **5**.


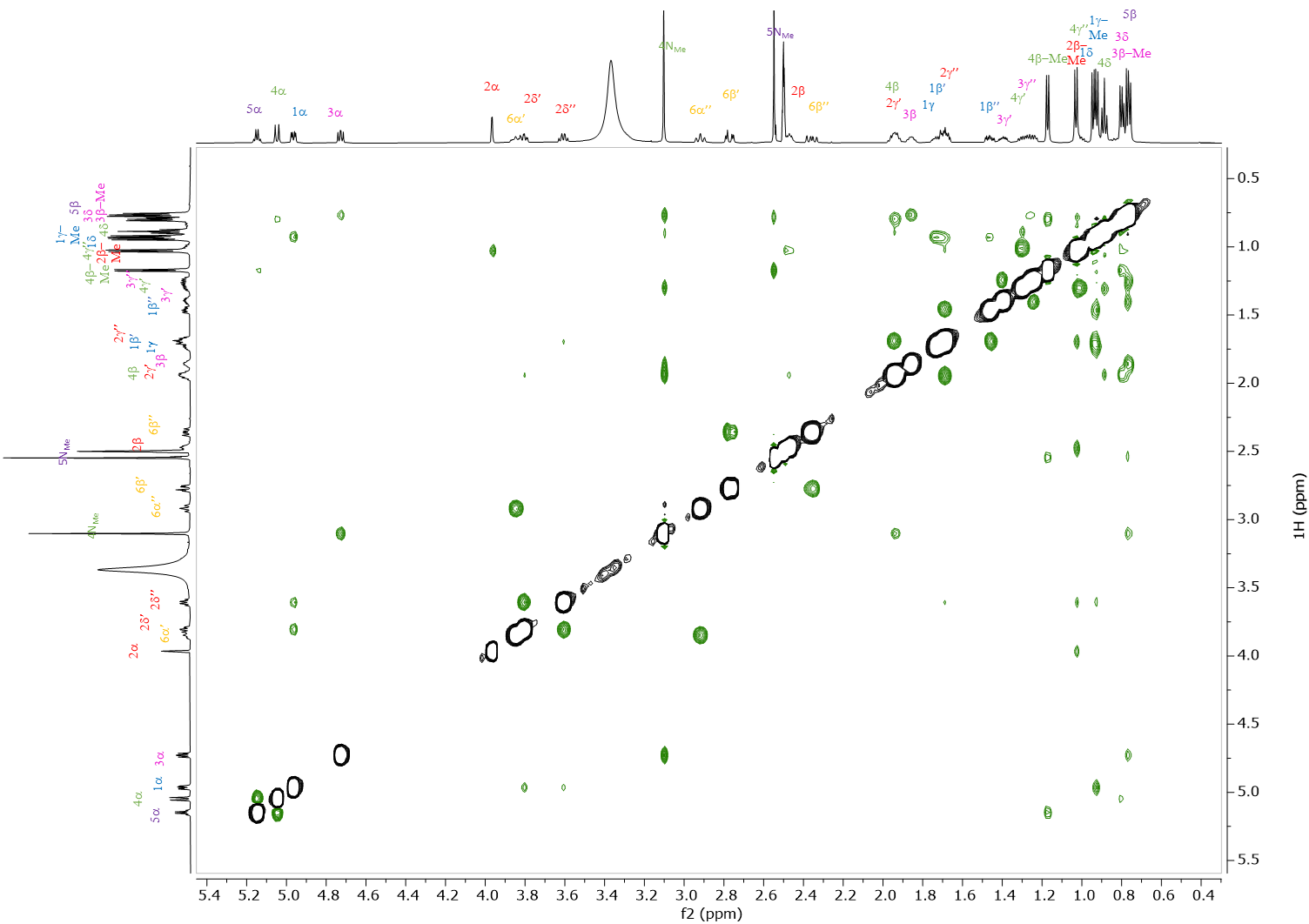


Figure S 95. ROESY (Expanded) spectrum of **5**.

Table S 1. ^1^H (600 MHz) and ^13^C (151 MHz) NMR data of **1** and **2** in DMSO-*d_6_*.

| Position | δ_C_, type | δ_H_, mult. (*J* in Hz) | Position | δ_C_, type | δ_H_, mult. (*J* in Hz) |
| --- | --- | --- | --- | --- | --- |
| **δ-Cl-DHPA^1^** |  |  | **δ-Cl-DHPA^1^** |  |  |
| 1C=O | 168.4 |  | 1C=O | 168.7 |  |
| 1α | 70.5, CH | 5.07, m* | 1α | 70.4, CH | 5.08, m* |
| 1β | 34.95, CH_2_ | 1.97, m* | 1β | 35.2, CH_2_ | 1.99, m* |
|  |  | 1.91, m* |  |  | 1.94, m* |
| 1γ | 66.3, CH | 3.71, m | 1γ | 66.6, CH | 3.64, m* |
| 1δ | 49.5, CH_2_ | 3.63, m* | 1δ | 49.4, CH_2_ | 3.64, m* |
| 1γ-OH |  | 5.42, s |  |  |  |
| **Pro^2^** |  |  | **β-Me-Pro^2^** |  |  |
| 2C=O | 170.4 |  | 2C=O | 170.3 |  |
| 2α | 60.1, CH | 4.40, d (7.9) | 2α | 66.6, CH | 4.00, d (2.1) |
| 2β | 23.5, CH_2_ | 2.00, m* | 2β | 36.5, CH | 2.47, m* |
|  |  | 1.76, m |  |  |  |
| 2γ | 29.2, CH_2_ | 2.08, m | 2γ | 30.3, CH_2_ | 1.94, m* |
|  |  | 2.00, m |  |  | 1.67, m |
| 2δ | 46.1, CH_2_ | 3.83, m* | 2δ | 44.6, CH_2_ | 3.88, m |
|  |  | 3.68, m* |  |  | 3.77, m |
|  |  |  | 2β-Me | 18.7 | 1.03, d (7.0) |
| **Ile^3^** |  |  | **Ile^3^** |  |  |
| 3C=O | 173.0 |  | 3C=O | 173.1 |  |
| 3α | 52.6, CH | 4.79, m | 3α | 52.5, CH | 4.77, dd (9.4, 6.6) |
| 3β | 36.8, CH | 1.82, m | 3β | 36.7, CH | 1.83, m |
|  |  |  |  |  |  |
| 3γ | 23.8, CH_2_ | 1.38, ddd (13.6, 7.5, 3.7) | 3γ | 23.8, CH_2_ | 1.38, m |
|  |  | 1.23, ddd* (13.4, 8.7, 7.0) |  |  | 1.23, m* |
| 3δ | 11.2, CH_3_ | 0.77, t* (7.4) | 3δ | 11.2, CH_3_ | 0.78, t* (7.4) |
| 3β-Me | 15.1, CH_3_ | 0.77, d* (6.9) | 3β-Me | 15.1, CH_3_ | 0.77, d* (7.2) |
| 3NH |  | 6.95, d (9.4) | 3NH |  | 6.91, d (9.4) |
| **Ile^4^** |  |  | **Ile^4^** |  |  |
| 4C=O | 170.6 |  | 4C=O | 170.4 |  |
| 4α | 55.8, CH | 5.06, d | 4α | 55.8, CH | 5.07, m* |
| 4β | 32.86, CH | 1.93, m* | 4β | 32.8, CH | 1.96, m* |
| 4γ | 25.0, CH_2_ | 1.30, ddd* (13.6, 7.6, 2.2) | 4γ | 25.0, CH_2_ | 1.29, m* |
|  |  | 1.02, m |  |  | 1.00, m |
| 4δ | 10.9, CH_3_ | 0.89, t (7.4) | 4δ | 10.9, CH_3_ | 0.88, t (7.4) |
| 4β-Me | 15.3, CH_3_ | 0.81, d (6.6) | 4β-Me | 15.3, CH_3_ | 0.80, d (6.6) |
| 4N-Me | 30.6, CH_3_ | 3.11, s | 4N-Me | 30.6, CH_3_ | 3.11, s |
| **N-Me-Ala^5^** |  |  | **N-Me-Ala^5^** |  |  |
| 5C=O | 168.9 |  | 5C=O | 168.9 |  |
| 5α | 54.8, CH | 5.14, q (6.6) | 5α | 54.8, CH | 5.13, q (6.7) |
| 5β | 15.4, CH_3_ | 1.17, d (6.7) | 5β | 15.4, CH_3_ | 1.17, d (6.6) |
| N-Me | 27.8, CH_3_ | 2.54, s | N-Me | 27.8, CH_3_ | 2.54, s |
| **β-Ala^6^** |  |  | **β-Ala^6^** |  |  |
| 6C=O | 173.2 |  | 6C=O | 173.3 |  |
| 6α | 32.9, CH_2_ | 3.84, m* | 6α | 32.9, CH_2_ | 3.83, m* |
|  |  | 2.93, t (12.9) |  |  | 2.93, m |
| 6β | 34.1, CH_2_ | 2.77, dd (18.3, 5.4) | 6β | 34.2, CH_2_ | 2.78, m |
|  |  | 2.36, m |  |  | 2.36, ddd (18.4, 11.5, 2.0) |
| 6NH |  | 8.06, dd (9.9, 2.2) | 6NH |  | 8.08, dd (10.0, 2.6) |

Table S 2. ^1^H (600 MHz) and ^13^C (151 MHz) NMR data of **3** in DMSO-*d_6_*.

| Position | δ_C_, type | δ_H_, mult. (*J* in Hz) |
| --- | --- | --- |
| **HP-e-A^1^** |  |  |
| 1C=O | 168.5 |  |
| 1α | 71.9, CH | 5.04, t* (7.3) |
| 1β | 34.6, CH2 | 2.5, m* |
|  |  |  |
| 1γ | 131.6, CH | 5.80, m* |
| 1δ | 119.2, CH2 | 5.15, m* |
|  |  |  |
| **β-Me-Pro^2^** |  |  |
| 2C=O | 170.2 |  |
| 2α | 66.5, CH | 3.95, d (2.1) |
| 2β | 36.2, CH | 2.46, m |
|  |  |  |
| 2γ | 30.2, CH2 | 1.92, m* |
|  |  | 1.64, m |
| 2δ | 44.7, CH2 | 3.75, td (9.6, 2.9) |
|  |  | 3.64, m |
| 2β-Me | 18.5, CH3 | 1.00, d (7.0) |
| **Ile^3^** |  |  |
| 3C=O | 173 |  |
| 3α | 52.5, CH | 4.74, dd (9.4, 6.8) |
| 3β | 36.7, CH | 1.83, m |
|  |  |  |
| 3γ | 23.8, CH2 | 1.38, m |
|  |  | 1.23, m* |
| 3δ | 11.1, CH3 | 0.77, t* (7.5) |
| 3β-Me | 15.0, CH3 | 0.76, d* (7.0) |
| 3NH |  | 6.89, d (9.4) |
| **Ile^4^** |  |  |
| 4C=O | 170.3 |  |
| 4α | 55.7, CH | 5.06, d* (11.0) |
| 4β | 32.7, CH | 1.94, m* |
| 4γ | 24.9, CH2 | 1.29, m* |
|  |  | 1.02, m |
| 4δ | 10.8, CH3 | 0.88, t (7.4) |
| 4β-Me | 15.2, CH3 | 0.80, d (6.6) |
| 4N-Me | 30.5, CH3 | 3.11, s |
| **N-Me-Ala^5^** |  |  |
| 5C=O | 168.9 |  |
| 5α | 54.7, CH | 5.13, m* |
| 5β | 15.3, CH3 | 1.17, d (6.6) |
| N-Me | 27.7, CH3 | 2.54, s |
| **β-Ala^6^** |  |  |
| 6C=O | 173.1 |  |
| 6α | 32.8, CH2 | 3.84, m |
|  |  | 2.92, m |
| 6β | 34.0, CH2 | 2.76, m |
|  |  | 2.37, m |
| 6NH |  | 8.08, dd (10.0, 2.6) |

Table S 3. ^1^H (600 MHz) and ^13^C (151 MHz) NMR data of **4** and **5** in DMSO-*d_6_*.

| Position | δ_C_, type | δ_H_, mult. (*J* in Hz) | Position | δ_C_, type | δ_H_, mult. (*J* in Hz) |
| --- | --- | --- | --- | --- | --- |
| **α-HMPA^1^** |  |  | **α-HMPA^1^** |  |  |
| 1C=O | 168.93 |  | 1C=O | 169.1 |  |
| 1α | 71.6, CH | 4.95,dd (10.2, 3.3) | 1α | 71.4, CH | 4.96,dd (9.9, 3.7) |
| 1β | 38.3, CH2 | 1.67, ddd (14.6, 10.2, 4.6) | 1β | 38.4, CH2 | 1.69, m |
|  |  | 1.45, ddd (14.3, 9.0, 3.3) |  |  | 1.46, ddd (14.1, 8.5, 3.6) |
| 1γ | 23.9, CH | 1.74, m* | 1γ | 23.8, CH | 1.74, m* |
| 1δ | 23.2, CH3 | 0.93, d (6.7) | 1δ | 23.1, CH3 | 0.94, d (6.6) |
| 1γ-Me | 21.4, CH3 | 0.91, d (6.6) | 1γ-Me | 21.4, CH3 | 0.92, d (6.5) |
| **Pro^2^** |  |  | **β-Me-Pro2** |  |  |
| 2C=O | 170.4 |  | 2C=O | 170.2 |  |
| 2α | 60.0, CH | 4.37, m | 2α | 66.4, CH | 3.97, d (2.2) |
| 2β | 29.0, CH | 2.10, dd (11.2, 6.2) | 2β | 36.4, CH | 2.47, m |
|  |  | 1.99, m* |  |  |  |
| 2γ | 23.6, CH2 | 2.01, m* | 2γ | 30.3, CH2 | 1.94, m* |
|  |  | 1.75, m* |  |  | 1.68, m* |
| 2δ | 46.1, CH2 | 3.86, m* | 2δ | 44.6, CH2 | 3.81, m* |
|  |  | 3.46, m* |  |  | 3.61, m |
|  |  |  | 2β-Me | 18.6, CH3 | 1.03, d (7.0) |
| **Ile^3^** |  |  | **Ile^3^** |  |  |
| 3C=O | 173 |  | 3C=O | 173.0 |  |
| 3α | 52.7, CH | 4.76, dd (9.3, 6.7) | 3α | 52.5, CH | 4.73, dd (9.3, 6.9) |
| 3β | 36.7, CH | 1.85, m | 3β | 36.6, CH | 1.86, m |
|  |  |  |  |  |  |
| 3γ | 23.87, CH2 | 1.39, ddd (13.6, 7.4, 3.7) | 3γ | 23.9, CH2 | 1.40, ddd (13.7, 7.5, 3.7) |
|  |  | 1.24, m* |  |  | 1.24, m* |
| 3δ | 11.1, CH3 | 0.77, t* (7.5) | 3δ | 11.1, CH3 | 0.78, t (7.5) |
| 3β-Me | 15.1, CH3 | 0.76, d* (7.7) | 3β-Me | 15.3, CH3 | 0.80, d (6.5) |
| 3NH |  | 6.97, d (9.3) | 3NH |  | 6.91, d (9.3) |
| **Ile^4^** |  |  | **Ile^4^** |  |  |
| 4C=O | 170.6 |  | 4C=O | 170.3 |  |
| 4α | 55.9, CH | 5.04, d (11.0) | 4α | 55.8, CH | 5.05, d (11.0) |
| 4β | 32.8, CH | 1.94, m* | 4β | 32.8, CH | 1.94, m |
| 4γ | 25.0, CH2 | 1.30, m* | 4γ | 24.9, CH2 | 1.30, m* |
|  |  | 1.02, m |  |  | 1.01, m |
| 4δ | 10.9, CH3 | 0.89, t (7.4) | 4δ | 10.8, CH3 | 0.89, t (7.4) |
| 4β-Me | 15.3, CH3 | 0.81, d (6.5) | 4β-Me | 15.4, CH3 | 1.17, d (6.6) |
| 4N-Me | 30.6, CH3 | 3.11, s | 4N-Me | 30.5, CH3 | 3.10, s |
| **N-Me-Ala^5^** |  |  | **N-Me-Ala^5^** |  |  |
| 5C=O | 168.9 |  | 5C=O | 168.9 |  |
| 5α | 54.8, CH | 5.15, q (6.7) | 5α | 54.7, CH | 5.15, q (6.7) |
| 5β | 15.4, CH3 | 1.18, d (6.7) | 5β | 14.9, CH3 | 0.76, d (7.0) |
| N-Me | 27.7, CH3 | 2.54, s | N-Me | 27.7, CH3 | 2.54, s |
| **β-Ala^6^** |  |  | **β-Ala^6^** |  |  |
| 6C=O | 173.3 |  | 6C=O | 173.3 |  |
| 6α | 32.9, CH2 | 3.84, m* | 6α | 32.9, CH2 | 3.85, m* |
|  |  | 2.92, m |  |  | 2.92, m |
| 6β | 34.0, CH2 | 2.76, m | 6β | 33.9, CH2 | 2.77, dd (18.1, 5.1) |
|  |  | 2.36, ddd (18.6, 11.4, 1.9) |  | | 2.36, ddd (18.5, 11.6, 2.0) |
| 6NH |  | 8.06, dd (10.0, 2.5) | 6NH |  | 8.08, dd (10.1, 2.5) |

Figure S 96. The key HMBC (blue arrows) and ^1^H-^1^H COSY (blue bond lines) correlations of **1**–**5**.

**Bioactivity Data**

**Cytotoxic activity and calculation of IC_50_ values of 1‒5**

Compounds **1**‒**5** were tested for their cytotoxic effects against several human cancer cell lines (MOLM-13, MV-4-11, THP-1, A2058, MCF7, and MRC-5) using an MTS assay at concentrations ranging between 0.002 to 80 µM, depending upon potency. The results were plotted in a dose-response curve as shown in Figure S97 and the half-maximum inhibitory concentration (IC_50_) values were calculated using R. A 4-parameter logistic (4PL) dose-response model was applied to analyse the relationship between the concentration and the observed response (absorbance at 490 nm). This resulted in a sigmoidal (log-transformed) concentration-response curve. The model was fitted to the experimental data using the *drm* function from the *drc_3.0-1* package in R and IC_50_ values were determined as the concentration (μM) corresponding to 50% cell survival [1,2].

**References**

1. R Core Team. R: A language and environment for statistical computing. In*.*: R Foundation for Statistical Computing, Vienna, Austria; 2025.

2. Ritz, C.; Baty, F.; Streibig, J. C.; Gerhard, D. Dose-Response Analysis Using R. PLoS One 2015, 10 (12), e0146021. DOI: https://doi.org/10.1371/journal.pone.0146021.

Figure S 97. Dose-response curve showing the survival percentage of (a) THP-1, (b) MOLM-13, (c) MV-4-11, (d) MCF7, and (e) A2058 cells treated with compounds **1**‒**5** at different concentrations. The survival % were measured using MTS assay. Data represent the mean values from three technical replicates.

**FLT3 Inhibitory Activity of 1‒5**

Figure S 98. Dose-response (DR) curves for the FLT3 WT enzyme inhibitory activity of quizartinib and compounds **1**‒**5**. The Curves were obtained by plotting the graph between log_10_ concentrations of the compounds (nM) and emission ratios (Y-axis). Error bars represent the standard deviation of three technical replicates. Compounds **1**‒**5** did not inhibit FLT3 WT at the concentration range tested (12 nM‒50 µM).

**PTP1B Activity of 1‒5.**

PTP1B enzyme inhibition of **1**‒**5** was assessed by measuring the residual activity of PTP1B enzyme. The equation for calculating residual activity is as follows.

Residual enzyme activity (%) $=\frac{Fc-Fi}{Fn-Fi} 100\%$

Where:

Fc: average fluorescence of test compound

Fi: average fluorescence of PTP1B inhibitor

Fn: average fluorescence of negative control

Figure S 99. Residual PTP1B enzyme activity of **1**‒**5**. The compounds were tested at 100 µM with two technical replicates. None of the compounds were active, as PTP1B enzyme activity of all the compounds was above 75%. The enzyme activity should be below 30% to be considered active.
